# Ezh2 harnesses the intranuclear actin cytoskeleton to remodel chromatin in differentiating Th cells

**DOI:** 10.1016/j.isci.2021.103093

**Published:** 2021-09-09

**Authors:** Moran Titelbaum, Boris Brant, Daniel Baumel, Alina Burstein-Willensky, Shira Perez, Yiftah Barsheshet, Orly Avni

**Affiliations:** 1 Azrieli Faculty of Medicine, Bar-Ilan University, Safed, Israel

**Keywords:** Immunology, Biological sciences, Cell biology

## Abstract

Following their first interaction with the antigen, quiescent naive T-helper (Th; CD4^+^) cells enlarge, differentiate, and proliferate; these processes are accompanied by substantial epigenetic alterations. We showed previously that the epigenetic regulators the polycomb-group (PcG) proteins have a dual function as both positive and negative transcriptional regulators; however, the underlying mechanisms remain poorly understood. Here, we demonstrate that during Th cell differentiation the methyltransferase activity of the PcG protein Ezh2 regulates post-transcriptionally inducible assembly of intranuclear actin filaments. These filaments are colocalized with the actin regulators Vav1 and WASp, vertically oriented to the T cell receptor, and intermingle with the chromatin fibers. Ezh2 and Vav1 are observed together at chromatin-actin intersections. Furthermore, the inducible assembly of nuclear actin filaments is required for chromatin spreading and nuclear growth. Altogether these findings delineate a model in which the epigenetic machinery orchestrates the dynamic mechanical force of the intranuclear cytoskeleton to reorganize chromatin during differentiation.

## Highlights

• Ezh2 regulates post-transcriptionally the inducible assembly of intranuclear F-actin
• F-actin is oriented toward the TCR and intermingled with the chromatin fibers
• F-actin is required for chromatin spreading and nuclear growth
• The epigenetic machinery harnesses intranuclear cytoskeleton to reorganize chromatin

## Graphical abstract

**Figure:**
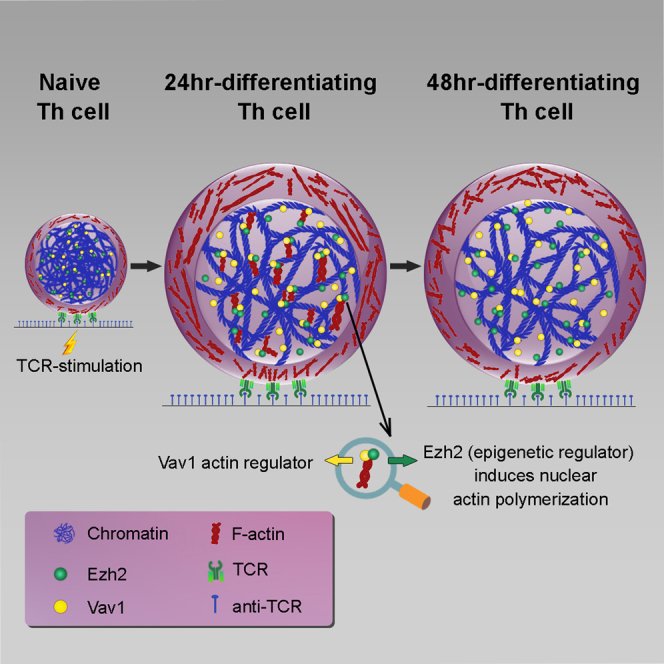

## Introduction

The immune system distinguishes between self and non-self but also between different types of non-self, such as viruses, bacteria, and worms. Th cells have a critical role in instructing the strategy of the immune response to these diverse challenges (Meningher et al., 2020; Werbner et al., 2019). Naive Th cells are actively maintained quiescent in a hypo-responsiveness state that is characterized by small cell size, low proliferative rate, and minimal basal metabolism (Chapman et al., 2019; Wolf et al., 2020). When a naive Th cell encounters the appropriate antigen for the first time ^_^ which is presented usually by dendritic cell in a lymph node ^_^ depending on the context, it differentiates down to either regulatory or effector lineage (Ansel et al., 2006; Avni and Rao, 2000; Lee et al., 2006; Sallusto, 2016; Wilson et al., 2009; Zhou and Littman, 2009). The effector Th1 and Th2 lineages are characterized by the expression of the signature cytokines interferon-γ (IFNγ) and interleukin-4 (IL-4), respectively. IFNγ exerts protective functions during intracellular infections and is involved clinically in cases of autoimmune diseases, whereas IL-4 is effective during parasitic infection but is also implicated in allergic reaction (Avni and Avni, 2021; Avni and Koren, 2018; Shamriz et al., 2016).

During early stages of Th cell differentiation, the naive cells become epigenetically competent for subsequent expression of the lineage-specific transcriptional programs in response to antigen stimulation. This process is associated with major changes in the chromatin structure (Avni et al., 2002; Hakim et al., 2013). In previous studies, we demonstrated that the polycomb-group (PcG) proteins, which were known as transcriptional repressors, function unconventionally in Th cells also as transcriptional activators (Hod-Dvorai et al., 2011; Jacob et al., 2008, 2011). The PcG proteins form two major complexes, the PcG repressive complex 1 (PRC1) and PRC2. PRC2 contains the core proteins Ezh2 (or Ezh1), Suz12, and Eed (Chan and Morey, 2019; Schuettengruber et al., 2017; Yu et al., 2019). Ezh2 is histone methyltransferase that preferentially tri-methylates histone H3 on lysine 27 (H3K27me3), but additional targets were demonstrated (Laugesen et al., 2019; Margueron and Reinberg, 2010; Schuettengruber et al., 2007), and PcG proteins entail non-catalytic activities as well (Muller and Verrijzer, 2009; Pietersen and van Lohuizen, 2008; Schwartz and Pirrotta, 2008; Simon and Kingston, 2009). The PcG proteins are involved in the regulation of higher order chromatin structures, although the underlying machinery is unclear yet (Bantignies et al., 2011; Loubiere et al., 2019; Muller and Kassis, 2006; Schwartz and Pirrotta, 2007). Most of the studies on the PcG proteins were focused on their nuclear functions as epigenetic regulators. PcG proteins are found also at the cytoplasm, and the cytoplasmic Ezh2 contributes to T cell receptor (TCR)-driven actin polymerization (Su et al., 2005) and signaling (Dobenecker et al., 2018). It interacts with cytoplasmic Vav1 (Hobert et al., 1996; Venkatesan et al., 2018), a GDP/GTP nucleotide exchange factor that regulates actin polymerization downstream to various receptors (Katzav, 2015). In dendritic cells, Vav1 regulates migration through Ezh2-dependent talin-methylation (Gunawan et al., 2015; Venkatesan et al., 2018).

In the recent years, several studies demonstrated nuclear formation of actin filaments (F-actin). Nuclear F-actin involved in the regulation of gene expression, including of certain cytokines in differentiated Th cells (Plessner et al., 2015; Tsopoulidis et al., 2019; Wei et al., 2020), re-localization of DNA breaks for homology-directed repair (Caridi et al., 2018; Evdokimova et al., 2018; Schrank et al., 2018), DNA replication (Lamm et al., 2020; Parisis et al., 2017), and chromatin organization and nuclear expansion during mitotic exit (Baarlink et al., 2017). However, the interaction between the F-actin and chromatin is unexplored yet. Here we demonstrate that the epigenetic machinery engages the intranuclear actin skeleton to chromatin remodeling, which characterizes the transition of a naive Th cell into an effector differentiated Th cell.

## Results

### Transiently induced-actin filaments are colocalized with chromatin fibers in differentiating Th cells

To explore potential appearance of nuclear F-actin in differentiating Th cells, naive CD4^+^ Th cells were purified from spleen and lymph nodes of young mice, and stimulated with anti-CD3ε- and anti-CD28 antibodies, mimicking TCR stimulation, for 24 h and 48 h in the presence of either Th1- or Th2-polarizing cytokines. This skewed stimulation potentiates the naive Th cells to exit the quiescence state and differentiate, a process which is associated with extensive epigenetic, morphologic, and metabolic remodeling. Completion of the first cell division occurs within ∼30 h of stimulation (Chapman et al., 2019; Chapman and Chi, 2018), and is followed by a high proliferative rate. The 48 h-differentiating Th cells already possess either the Th1 or the Th2 phenotype but require re-TCR-stimulation for robust expression of cytokine and other effector genes.

**Figure 1:**
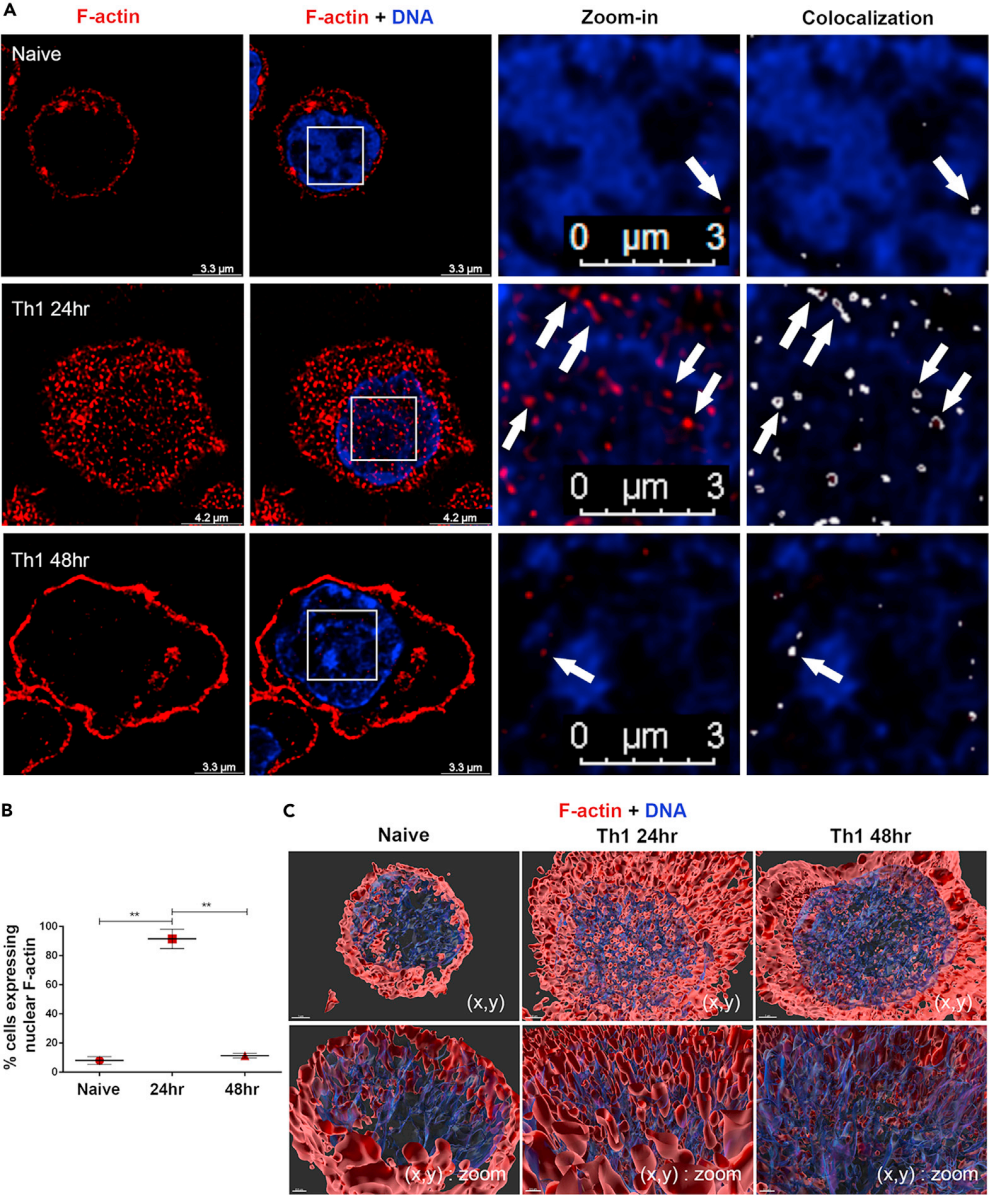

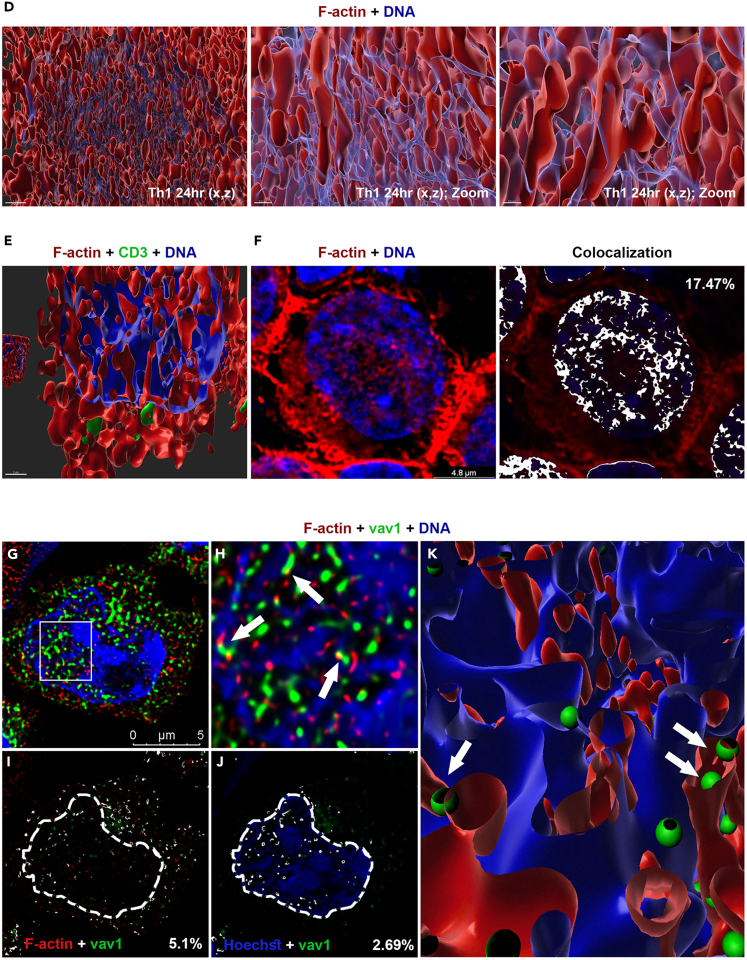
Inducible nuclear F-actin in the 24 h differentiating Th cells (A) Immunofluorescence staining of naive, 24 h- and 48 h-differentiating Th1 cells using DAPI (blue) and Phalloidin conjugated to biotin followed by cy3-streptavidin (red). Colocalization rate of the merged channels of nuclear F-actin and chromatin (overlapping signal in white). Images of wider fields and of the Th2 staining are presented in Figure S1A. (B) Comparison of the percentage of the visible nuclear F-actin-harbored cells between naive (n = 230), 24 h (n = 185)- and 48 h (n = 176)- differentiating Th1 cells. Data are represented as mean ± SEM. p values were computed with proportion Z-tests and exact Fischer’s test ∗p < 0.05, ∗∗p < 0.01, ∗∗∗p < 0.001. (C) Z stacks of representative images of naive and Th1 cells from (A) were combined into a computational 3D-structures using the IMARIS 9.5 software (chromatin in transparent blue to facilitate F-actin visualization, and F-actin in red). The images are presented at the XY direction. The Th2 computational images are shown in Figure S1B. (D) Computational 3D-structure of 24 h-differentiating Th1 cells are presented at the XZ direction. (E) The differentiating Th cells were stimulated directly on the slide with anti-CD3 and anti-CD28 abs to maintain the orientation toward the TCR. The staining procedure was performed directly on the slide. Computational 3D-structure at XZ direction presenting F-actin (red), CD3ε (green), and chromatin (blue). Wider fields and zoom-in images are presented in Figures S1C. The experiments were performed in two independent biological replicates with similar results. (F) (left) Immunofluorescence staining of 24 h-differentiating Th1 cells applying the cytospin protocol, using Phalloidin conjugated to biotin followed by cy3-streptavidin (red) and Hoechst (blue). (Right) Colocalization rate of nuclear F-actin and chromatin (overlapping signal in white). Images of Wider fields are presented in Figures S1C and S1D. The experiments were performed in two independent biological replicates with similar results. (G**–**K) (G) Immunofluorescence staining of the 24 h-differentiating Th1 cells using Phalloidin (red) and anti-Vav1 Ab (green). DNA was stained with Hoechst (blue). (H) Magnification of the white square in (I). The Th2 staining and images of wider fields are presented in Figure S1H. (I) Colocalization rate of nuclear F-actin and Vav1 (overlapping signal in white). The white dashed line, which was determined by Hoechst staining, defines the nuclear periphery (ROI) for nuclear colocalization assessment. (J) Colocalization rate of nuclear Vav1 and chromatin (overlapping signal in white). (K) Computational 3D-structure of Vav1, F-actin and chromatin. The Th2 images are presented in Figure S1K. Colocalization of Vav1, F-actin and chromatin in naive and 48 h differentiating Th cells are presented in Figures S1I and S1J. The experiments were performed in three independent biological replicates with similar results. Secondary Ab only was used as Control (Figure S1N). Images were acquired by super resolution (SR) Hyvolution microscope.

The freshly purified naive Th cells and the 24 h- and 48 h-differentiating Th1 and Th2 cells were co-stained with Phalloidin conjugated to biotin (for F-actin visualization) and DAPI/Hoechst (as indicated in the Figure legends for DNA probing). Super resolution (SR) microscopy (HyVolution) demonstrated an emergence of nuclear F-actin in the enlarging 24 h-differentiating Th1 and Th2 cells, which was colocalized with the chromatin fibers (Figures 1A and S1A). The naive and 48 h-differentiating Th cells scarcely presented visible nuclear actin filaments (the 48 h differentiating Th cells possess the characteristic dense actin ring structure at the cell periphery). The selective presence of nuclear F-actin in the 24 h-differentiating Th cells was significant (Figure 1B). LasX software confirmed the colocalization (white spots) of the nuclear F-actin with the chromatin fibers (Figure 1A). The circular pattern of the colocalized areas (white arrows) raised the idea that the chromatin fibers are wound around the nuclear actin filaments in the 24 h-differentiating Th cells. The z stack images of the naive, 24 h-, and 48 h-differentiating Th1 and Th2 cells were combined into 3D-computational structures using the IMARIS software (Figures 1C, 1D, and S1B). The F-actin filaments appeared mostly in clusters (with a rough estimate of 100 nm width, based on fluorescence measurement only; Figures 1C, 1D, and S1R) vertically oriented to the TCRs (that were mainly concentrated on the cellular side that was attached to the anti-TCR-associated surface during stimulation; Figures 1E, S1C, S1D, and S1E), and intermingled with the chromatin fibers (Figures 1C and 1D). Cytocentrifugation (cytospin) procedure during sample preparation was also applied, although this process distorts the original cellular 3D architecture, however, facilitates a wider overview (Figures 1F, S1E, and S1F). The nuclear F-actin was alternatively observed with the fluorogenic permeable probe sir-actin, applying super resolution microscopy (Stimulated Emission Depletion; STED) (Figure S1G).

Several studies have shown previously the presence of the actin regulator Vav1 in the nucleus (Houlard et al., 2002; Romero et al., 1996, 1998; Turner and Billadeau, 2002). SR microscopy demonstrated cytoplasmic and nuclear Vav1 in the naive, 24 h-, and 48 h-differentiating Th1 and Th2 cells (Figures 1G, 1H, and S1H). LasX software analysis (Figure 1I), and computational 3D-imaging (Figures 1K and S1K) revealed partial colocalization of Vav1 and F-actin in the nucleus of the 24 h-differentiating Th cells. Vav1 was also intensely colocalized with the chromatin in the 24 h-differentiating Th cells (Figures 1J and 1K), as well as in the naive and 48 h-differentiating Th cells (Figures S1I and S1J).

Cytoplasmic WASp regulates the induction of branched actin filaments by the actin polymerization-nucleating (Arp)2/3 complex, which was found recently as an important factor for the assembly of nuclear actin during double-strand break clearance (Caridi et al., 2018; Schrank et al., 2018). There is also evidence connecting WASp to transcriptional regulation (Taylor et al., 2010). SR microscopy followed by computational 3D-imaging indicated that WASp was mostly cytoplasmic, and less ubiquitous in the nucleus (Figures S1M and S1O–S1Q), but the nuclear WASp was colocalized in part with F-actin, Vav1, and chromatin in the 24 h-differentiating Th cells Altogether these findings reveal transiently inducible oriented nuclear actin filaments that are associated with actin regulators and interact with the chromatin fibers in the 24 h-differentiating Th1 and Th2 cells in correlation with nuclear growth.

### Nuclear Ezh2 is colocalized with F-actin and chromatin-associated Vav1

**Figure 2:**
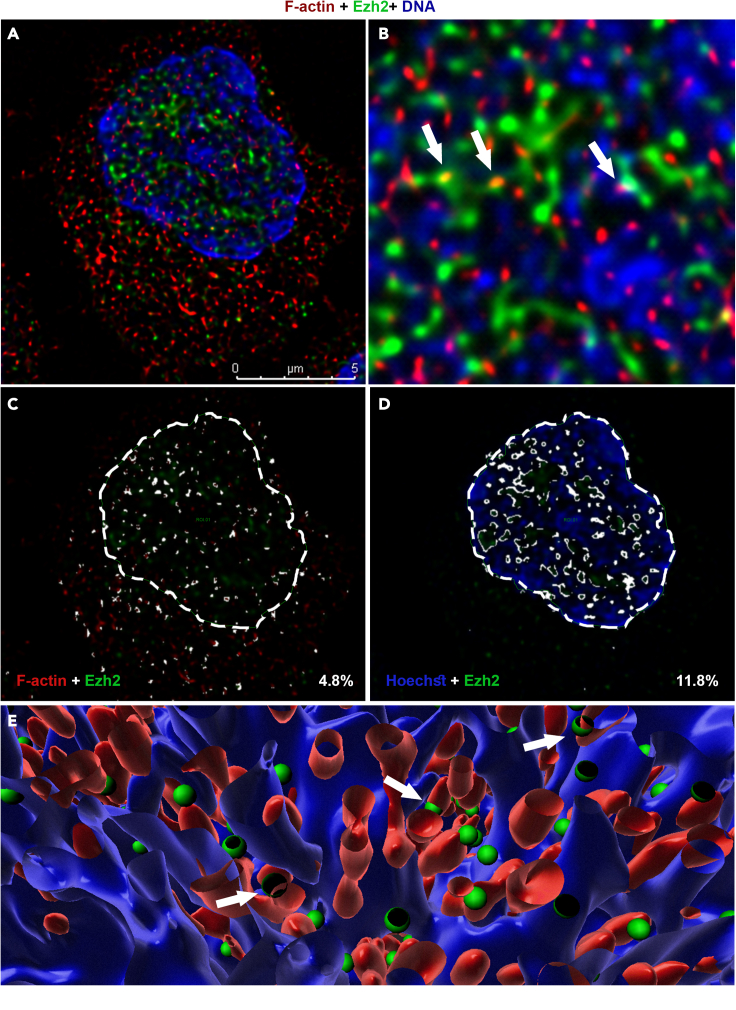
Ezh2 is associated with nuclear F-actin and actin machinery (A) Immunofluorescence staining of the 24 h-differentiating Th1 cells using Phalloidin (red) and anti-Ezh2 Abs (green). DNA was stained with Hoechst (blue). (B) Magnification of the white square in (A). (C) Colocalization rate of nuclear Ezh2 and F-actin (overlapping signal in white). The white dashed line, which was determined by Hoechst staining, defines the nuclear periphery (ROI) for nuclear colocalization assessment. (D) Colocalization rate of nuclear Ezh2 and chromatin. (E) Computational 3D-structure of F-actin, Ezh2 and chromatin. The images of wider Th2 fields are presented in Figures S2A and S2B. Association of Ezh2 with the chromatin in naive and 48 h-differentiating Th cells are presented in Figures S2C and S2D. The experiments were performed in three independent biological replicates with similar results.

**Figure 3:**
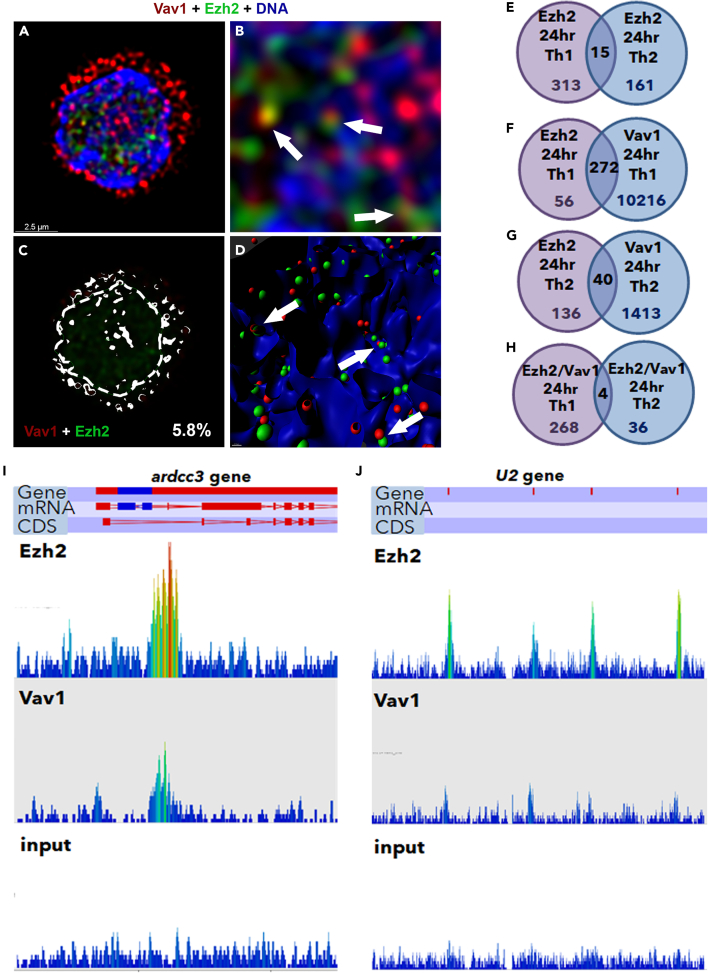
Nuclear Ezh2 is colocalized with chromatin-associated Vav1 (A–D) (A) Immunofluorescence staining of the 24 h-differentiating Th1 cells using anti-Ezh2 mouse monoclonal Ab followed by 488 anti-mouse (green), and anti-Vav1 Ab (red). DNA was stained with Hoechst. (B) Magnification of the white square in (I). (C) Colocalization rate of nuclear Ezh2 and Vav1 (overlapping signal in white). The white dashed line, which was determined by Hoechst staining, defines the nuclear periphery (ROI) for nuclear colocalization assessment. (D) Computational 3D-structure of Vav1 and Ezh2. The full images are presented in Figure S3A. The experiments were performed in three independent biological replicates with similar results. (E–H) ChIP assay assessing the chromatin binding activity of Ezh2 and/or Vav1 in the 24 h-differentiating Th cells (E) ChIP-seq assessing the binding activity of Ezh2 in Th1 and Th2 cells (Table S1). (F) ChIP-seq assessing proximal binding activity of Ezh2 and Vav1 around the same gene in Th1 cells (Table S2a). (G) ChIP-seq assessing proximal binding activity of Ezh2 and Vav1 around the same gene in Th2 cells (Table S2b). (H) ChIP-seq assessing the binding activity of Ezh2/Vav1 in both Th1 and Th2 cells. (I and J) Illustration of the binding activity of Ezh2 and Vav1 at the *ardcc3* and *U2* genes. The peaks were plotted using wiggle plot in Seqmonk free software. The illustration of the binding activity in the Th2 cells is presented in Figures S3I and S3J. The experiments were performed twice (FDR < 0.05).

To explore potential interactions between Ezh2 and F-actin in the nucleus, naive, 24 h- and 48 h-differentiating Th1 and Th2 cells were co-stained with anti-Ezh2 antibody, phalloidin, and Hoechst (Figures 2 and S2A). As previously demonstrated (Figure 1), visible nuclear F-actin appeared restrictedly in the 24 h-differentiating Th cells. Ezh2 was observed both in the nucleus and cytoplasm of naive and differentiating Th cells (Figures S2A and S2B). In the 24 h-differentiating Th1 and Th2 cells, nuclear Ezh2 was partially colocalized with the actin filaments (Figures 2B, 2C, 2E, S2A, and S2B), Vav1 (Figures 3A–3D and S3A), Wasp (Figures S3C, S3D and S3E), and, as expected, chromatin fibers (Figures 2A, 2B, 2D, and 2E). Ezh2 was not colocalized with nuclear DDB1, which was used as a negative control (Figure S3B).

Ezh2 was colocalized with the chromatin also in the naive and 48 h-differentiating Th cells (Figure S2C and S2D). ChIP-Seq analysis (chromatin immunoprecipitation followed by sequencing), widely mapping the genomic binding pattern of Ezh2 in the naive, 24 h- and 48 h-differentiating Th cells, demonstrated a developmental- and lineage-specific binding pattern (Figure S3F). In the 24 h-differentiating Th cells, the binding activity of Ezh2 possesses mostly Th1- and Th2-distinct peaks, but overlapping peaks were also observed (Figure 3E). To further characterize the interaction between Ezh2 and the actin machinery at the chromatin context in the 24 h-differentiating Th cells, a ChIP-seq assessing putative common binding pattern of Ezh2 and Vav1 (Ezh2/Vav1 peaks) was performed. Ezh2/Vav1 exactly overlapping peaks appeared mostly at promoters (using galaxy chipseeker algorithm; Figure S3G). In Th1 cells, 208 Ezh2/Vav1 peaks were proximal mainly to genes function in replication (such as histones), transcription (such as transcription factors), and splicing (such as *U1*), however some of these genes are associated with early promotion (*Cx3cr1*) or inhibition (*Prdm1*) of the Th1 fate (Figure S3H; Table S1). More generally, binding sites of Ezh2 and Vav1 were proximal around 272 genes (in a distance smaller than 5kb from Transcription Start Site; Figure 3F; Table S2a). In Th2 cells, 13 exact overlapping peaks of Ezh2/Vav1 were found (Table S1), and the binding sites of Ezh2 and Vav1 were proximal around 40 genes that cannot be categorized, possibly since the binding activity in Th2 is underestimated in our intersected ChIP assays (Figure 3G; Table S2b). However, genes that their function is associated with proliferation (*Kat8*) and early Th2 cell differentiation (*Stat3*) are included. Almost all the Ezh2/Vav1 peaks (exactly or proximally overlapped) were Th-lineage specific (Figure 3H; Table S1. Proximal genes (in distance smaller than 5kb from Transcription Start Site) to the Ezh2/Vav1 (exact overlapping) peaks in 24 h-differentiating Th1 and Th2 cells (FDR<0.05). Traces ChIP-seq, related to STAR Methods, Table S2. (a) Genes that are bound by both Ezh2 and Vav1 (in proximity) in the 24 h-differentiating Th1 cells (with FDR<0.05). Traces ChIP-seq, related to STAR Methods. (b) Genes that are bound by both Ezh2 and Vav1 (in proximity) in the 24 h-differentiating Th2 cells (with FDR<0.05). Traces ChIP-seq, related to STAR Methodsa and S2b), except at the *Arrdc3* that was bound exactly on the same sites by Ezh2/Vav1 in both Th1 and Th2 cells (Figures 3I and S3I), as well as three genes such as *U2* in which the binding sites of Ezh2 and Vav1 were in closed proximity at the gene locus (Figures 3J and S3J; Tables S2a and S2b). Since the majority of the Ezh2/Vav1 binding sites are lineage-restricted, although we cannot exclude underestimation of the binding activity due to either technical limitations or elusive dynamics, a differentiation-specific functional role is strongly suggested for the common binding sites of Ezh2 and Vav1.

To discover over-represented motifs in DNA sequences around the Ezh2/Vav1 binding sites, based on the assumption that enrichment of specific sequences may indicate biological functions, the computational program MEME motif-suite (MEME-ChIP and Tomtom (Gupta et al., 2007; Machanick and Bailey, 2011)) was employed. Sequences surrounded the Ezh2/Vav1 peaks in Th1 cells were enriched significantly with binding motifs of transcription factors such as the estrogen receptor, Blimp1, Myb, RAR, IRF, and STAT, and in Th2 cells with the binding motifs of ETS, RAR, and AP-1 (in Th2, at least in one of the experiments). However, since the common binding activity of Ezh2/Vav1 at the DNA was mostly around promoters, the existence of binding sites for transcription factors is expected, and their actual contribution to the recruitment of Ezh2 and Vav1 should be further studied.

**Figure 4:**
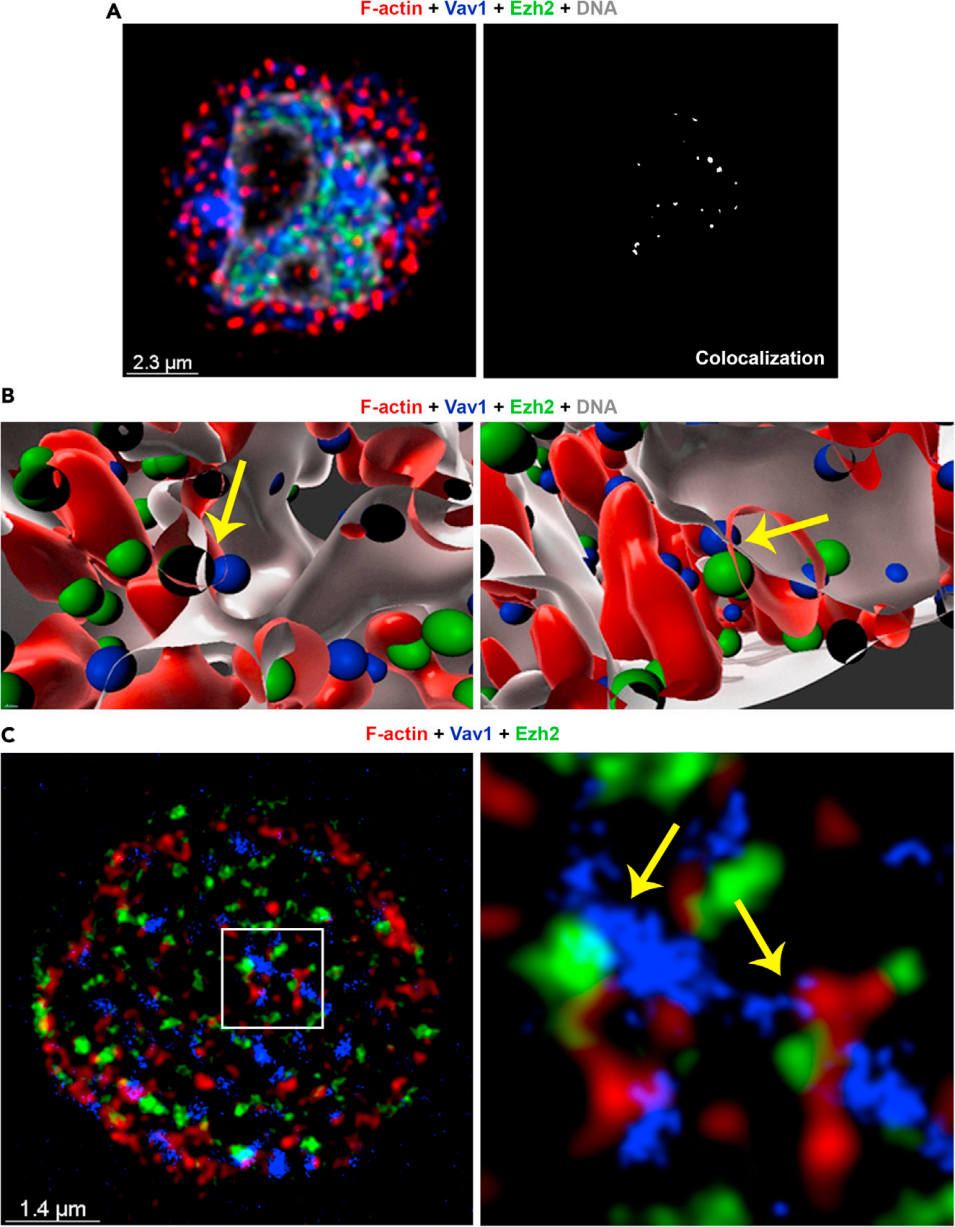
Nuclear Ezh2/Vav1 are colocalized with F-actin at the chromatin context (A) Immunofluorescence staining of the 24 h-differentiating Th1 cells using Phalloidin (red), anti-Ezh2 mouse monoclonal Ab (green) and anti-Vav1 Ab (blue), followed by colocalization (white spots). DNA was stained with Hoechst (gray). (B) Computational 3D-structures of the images in (A). (C) Immunofluorescence staining of the 24 h-differentiating Th1 cells using Phalloidin (red), anti-Ezh2 Ab (green), and anti-Vav1 Ab (blue). The image is followed by magnification of the white square. Images were acquired by SR STED microscope. The experiments were performed in three independent biological replicates with similar results.

SR microscopy followed by 3D-imaging revealed colocalization of Ezh2/Vav1 simultaneously with both chromatin fiber and actin filament, in many cases with one of the edges of the actin filament (Figures 4A, 4B, and S4A). STED microscopy validated the closed proximity between Ezh2, Vav1, and F-actin (Figure 4C). Similar results were obtained using alternative anti-Vav1 antibody (Figure S4B). These findings altogether demonstrate nuclear interactions between Ezh2, F-actin, and actin regulators at the chromatin context and raised the idea that the skewed stimulation orchestrates the activity of sequence specific factors, epigenetic regulators and nuclear skeleton to pursue the differentiation of naive Th cells.

### The assembly of the nuclear F-actin is Ezh2-dependent

**Figure 5:**
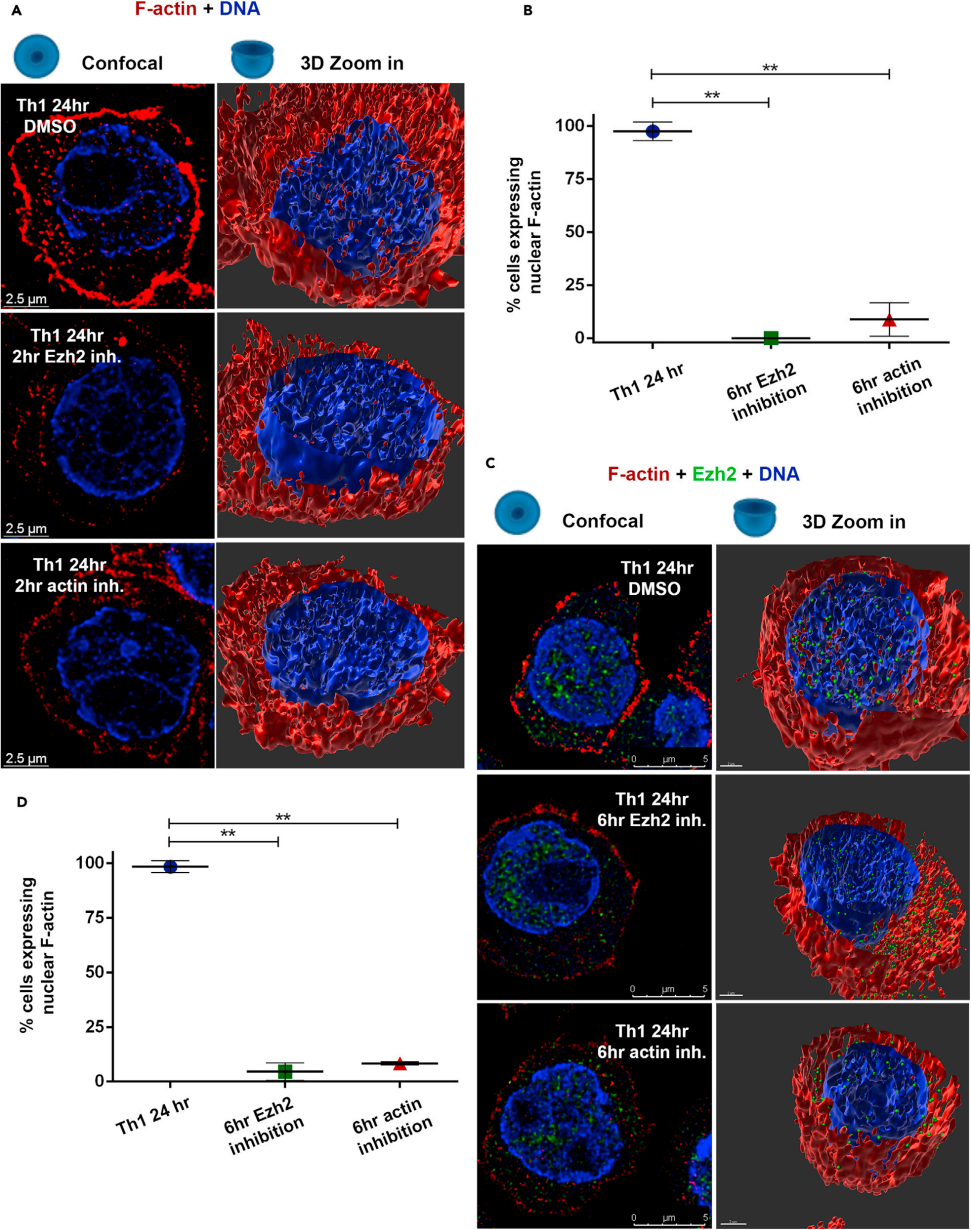
The assembly of F-actin is Ezh2-dependent (A) Immunofluorescence staining using Phalloidin (red), and Hoechst (blue) followed by computational 3D-image (IMARIS) of the 24 h-differentiating Th1 cells with or without the presence of either Ezh2 (1.75 mM UNC1999) or F-actin (10.4mM cytochalasin B) inhibitors for the last 2 h of stimulation. (B) Comparison of the percentage of visible nuclear F-actin harbored cells (mean values) between control 24 h (n = 42)-differentiating Th1 cells, differentiating Th1 cells with either Ezh2 inhibitor (n = 27)- or F-actin inhibitor (n = 20) for the last 6 h of stimulation from (A). The error bars represent the standard deviation. p values were computed with proportion Z-tests and exact Fischer’s test ∗p < 0.05, ∗∗p < 0.01, ∗∗∗p < 0.001. (C) Immunofluorescence staining using Phalloidin conjugated to biotin followed by cy3-streptavidin (red) and anti-Ezh2 rabbit polyclonal Ab followed by 488 anti-rabbit (green), and computational 3D-image (IMARIS) of the 24 h-differentiating Th1 cells with or without the presence of either Ezh2 (UNC1999) or F-actin (cytochalasin B) inhibitors for the last 6 h of stimulation. The Th2 staining and wider field images are presented in Figures S5A and S5B. (D) Comparison of the percentage of visible nuclear F-actin harbored cells between control 24 h (n = 57)-differentiating Th1 cells, differentiating Th1 cells with either Ezh2 inhibitor (n = 41)- or F-actin inhibitor (n = 36) for the last 2 h of stimulation from (B). Data are represented as mean ± SEM. p values were computed with proportion Z-tests and exact Fischer’s test ∗*p* < 0.05, ∗∗p < 0.01, ∗∗∗p < 0.001.

To assess the involvement of Ezh2 in the polymerization of nuclear actin in differentiating Th cells, naive Th cells were differentiated down the Th1 pathway for 24 h in the presence or absence of UNC1999, an inhibitor of Ezh2 (and Ezh1) methyltransferase activity, for either the last 2 h (Figures 5A, 5B, and S5A) or 6 h (Figures 5C, 5D, and S5B) of stimulation. UNC1999 was not applied simultaneously with stimulation due to the involvement of Ezh2 in the TCR signaling (Dobenecker et al., 2018; Su et al., 2005). Inhibition of Ezh2 methyltransferase activity for the last 2 h of stimulation, dramatically (in each cell; Figure 5A) and significantly (in most of the cells; Figure 5B) diminished the presence of nuclear F-actin, whereas inhibition for the last 6 h of stimulation, completely (Figure 5C) and significantly (Figure 5D) abolished it. Obviously, similar results were obtained using cytochalasin B that prevents actin polymerization (Figures 5, S5A, and S5B). The idea that Ezh2 is required for appropriate nuclear actin polymerization was supported by experiments using conditional deficient Ezh2 mice, although the ablation of Ezh2 in differentiating Th cells was more complicated to perform and interpreted (Figure S5C).

**Figure 6:**
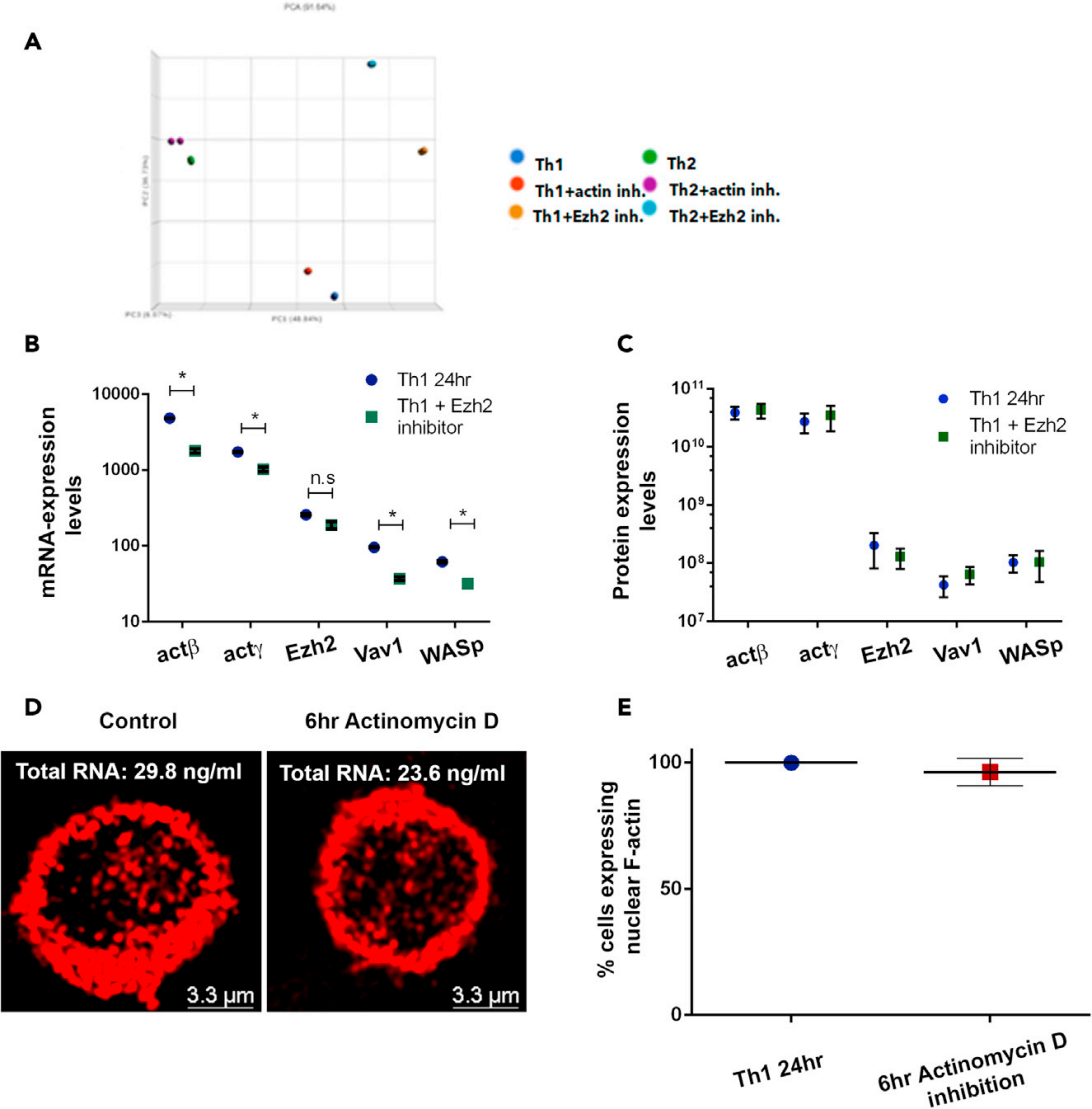
Ezh2-dependent methyltransferase activity regulates both the transcription of actin and post-transcriptionally the assembly of F-actin (A and B) (A) RNA-seq for the 24 h-differentiating Th1 and Th2 cells with or without the presence of either Ezh2 (UNC1999) or F-actin (cytochalasin B) inhibitors for the last 6 h of stimulation. Sequences were aligned using bowtie and analyzed for similarity by PCA plot, showing the differences between presence or absence of inhibitors. (B) comparison of the total mRNA counts of the indicated genes in the 24 h-differentiated Th1 cells with or without the presence of Ezh2 (UNC1999) for the last 6 h of stimulation. The lists of the mRNAs are presented in Tables S3a and S3b. Data are represented as mean ± SEM. Two-tailed t test was performed, p value<0.01 ∗, n.s – not significant, p value > 0.05. The expression levels in Th2 cells are presented in Figure S6B. (C) The expression levels of the indicated proteins in the 24 h-differentiated Th1 cells with or without the presence of UNC1999 for the last 6 h of stimulation as determined by nuclear proteomics. Data are represented as mean ± SEM. Two-tailed t test was performed, p value > 0.05. The expression levels in Th2 cells are presented in Figure S6C. (D and E) (D) Immunofluorescence staining using Phalloidin (red) of the 24 h-differentiating Th1 cells with or without the presence of actinomycin D (1μM) for the last 6 h of stimulation. The total RNA level was measured using Qbit (RNA BR Assay Kit). Wider field images of Th1 cells are presented in Figure S6D. (E) Comparison of the percentage of visible nuclear F-actin harbored cells between 24 h-differentiating cells with (n = 24) or without (n = 20) Actinomycin D. Data are represented as mean ± SEM. p values were computed with proportion Z tests and exact Fischer’s test p > 0.05.

The mRNA levels of *β-actin* and *γ-actin* and other relevant proteins such as Ezh2, Vav1, and WASp were reduced in the 24 h-differentiating Th cells in the presence of UNC1999 for the last 6 h of stimulation in comparison to the control as analyzed by RNA-seq (Figures 6A, 6B, S6A, and S6B; Tables S3a and S3b), indicating the involvement of Ezh2 in their transcriptional regulation. However, the presence of UNC1999 for the last 6 h of stimulation did not affect significantly the expression levels of the already translated β-actin, γ-actin, Ezh2, Vav1, and WASp, as was assessed by nuclear proteomics (Figures 6C and S6C), suggesting that Ezh2 regulates F-actin assembly, at least partially, post-transcriptionally. This conclusion was further supported by the fact that actinomycin D, a transcriptional inhibitor, did not interfere with the appearance of nuclear F-actin when added for the last 6 h of stimulation, although did reduce the total amount of nuclear RNA (Figures 6D, 6E, and S6D). These findings altogether indicate that Ezh2-dependent methyltransferase activity regulates both the transcription of actin and post-transcriptionally the intra-nuclear assembly of F-actin in differentiating Th cells.

### F-actin is required for chromatin spreading and nuclear expansion in differentiating Th cells

The functional role of Ezh2 in differentiation of Th cells was studied previously with some controversial results (He et al., 2013; Hod-Dvorai et al., 2011; Jacob et al., 2008, 2011; Tong et al., 2014; Tumes et al., 2013; Yang et al., 2015; Zhang et al., 2014). Integration of the list of Ezh2 bound genes in the 24 h-differentiating Th1 and Th2 cells (ChIP-seq results) and the mRNA expression levels of these genes with or without the presence of the Ezh2 inhibitor UNC1999 (RNA-seq data), revealed that the expression levels of the Ezh2-bound genes were mostly unchanged in the presence of the inhibitor, although some of the genes were down-regulated or up-regulated in more than 1.5-fold (Figures S7A and S7B; Tables S4a and S4b). These results support the idea of dual function of Ezh2 as either a positive or negative transcriptional regulator (Hod-Dvorai et al., 2011; Jacob et al., 2008, 2011), although not as the main function at this stage of differentiation. Whereas the presence of either UNC1999 or cytochalasin B eliminated the emergence of nuclear F-actin, the mRNA levels of most of Ezh2/Vav1 bound genes in either Th1 (Figure S7C; Tables S5a and S5b) or Th2 (Figure S7D; Tables S5c and S5d) cells, were not dramatically changed by these inhibitors (most of the genes were neither up- nor down-regulated by more than 1.5-fold). Moreover, even though the binding activity of Ezh2/Vav1 was lineage-specific, the expression pattern of their bound genes was not (Figure S7E; Table S6). Therefore, despite the fact that many of the Ezh2/Vav1 binding sites lies inside promoters, we hypothesized that the common activity of Ezh2 and Vav1 in the context of chromatin at this early stage of differentiation is associated with other functions rather than merely immediate transcriptional regulation.

**Figure 7:**
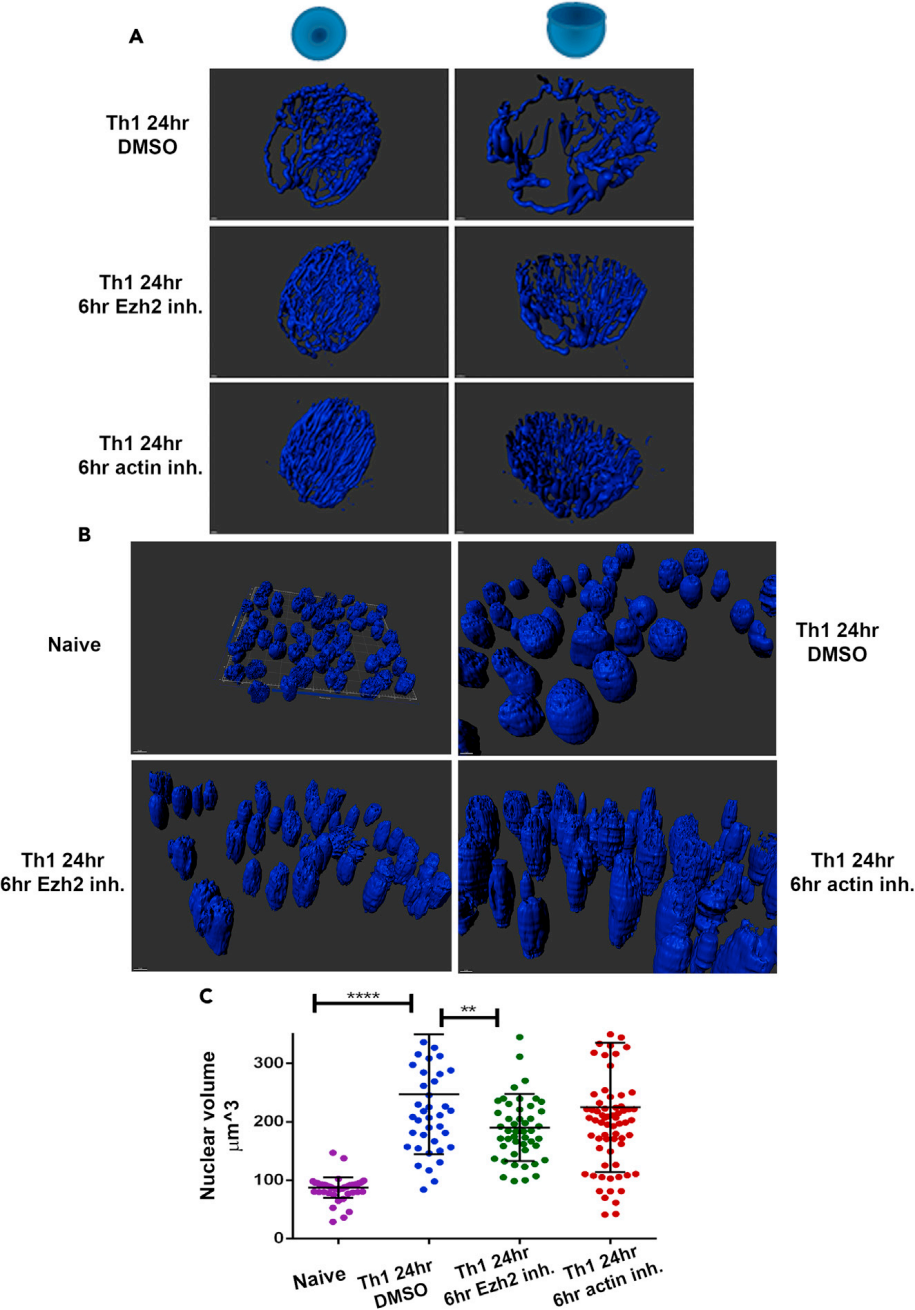
Ezh2 regulates chromatin spreading and nuclear expansion (A) Computational 3D-image of the 24 h-differentiating Th1 cells with or without the presence of either UNC1999 or cytochalasin B for the last 6 h of stimulation. DNA was stained with Hoechst. (B) Wider field images of (A) (including of the naive Th cells). (C) Quantitative analysis using IMARIS (surface application was applied) of nuclear volume of the Naïve (n = 197), 24 h differentiating Th1 cells with either Ezh2 inhibitor (n = 174), F-actin inhibitor (n = 177) or without (n = 190). Data are represented as mean ± SEM. Using PRISM software analysis, two-tailed t test was performed, p value < 0.0001 ∗∗∗∗, p value < 0.05 ∗∗.

The appearance of the oriented actin filaments in correlation with nuclear growth hinted at possible involvement of Ezh2 and F-actin in differentiation-induced alterations in chromatin architecture. Employing the Micrococcal Nuclease digestion assay (MNase) for naive and 24 h-differentiating Th1 and Th2 cells, suggested that Ezh2 activity does not significantly affect chromatin decompaction during the early 24 h of Th cell differentiation (Figure S7F). However, computational 3D-imaging comparing global chromatin structure in the 24 h-differentiating Th1 cells with or without the presence of UNC1999 for the last 6 h of stimulation, demonstrated that Ezh2 is required for chromatin spreading (Figure 7A); inhibition of the methyltransferase activity of Ezh2 resulted in a more condensed organization of the chromatin fibers. The usage of cytochalasin B similarly indicated the necessity of polymerized actin for chromatin spreading in differentiating Th cells (Figure 7A).

**Figure 8:**
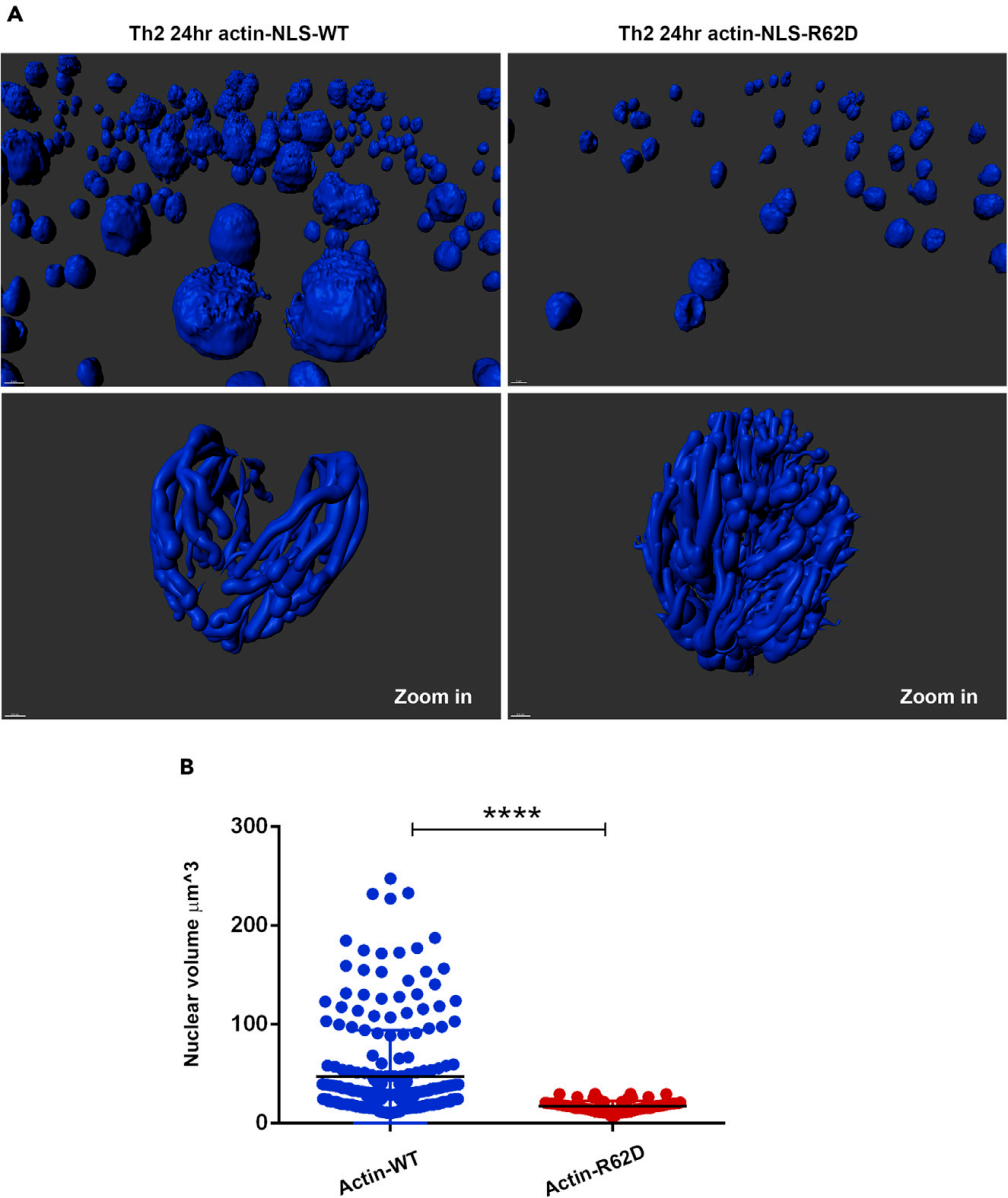
Nuclear F-actin drives chromatin spreading and nuclear expansion (A) Computational 3D-imaging of the chromatin (stained with Hoechst) in the 24 h-differentiating Th2 cells, which were electroporated with either Actin-NLS-R62D (n = 161) mRNA or Actin-NLS-WT (n = 276) mRNA as a control, 6 h before harvesting. Wider fields of the control images demonstrating the downregulation of nuclear actin in the presence of Actin-NLS-R62D is presented in Figure S8. (B) Quantitative analysis using IMARIS (surface application was applied) of nuclear volume of the Th2 cells from (A). Data are represented as mean ± SEM. Using PRISM software analysis, two-tailed t test was performed, p value < 0.0001 ∗∗∗∗, p value < 0.05 ∗∗.

Quantitative analysis by the IMARIS software demonstrated a significant decrease in the nuclear volume of the 24 h-differentiating Th1 cells that cultured in the presence of UNC1999 for the last 6 h of stimulation (Figures 7B and 7C). The shape of the UNC1999-treated nuclei was less globular and more elliptical (Figure 7B). The shape of the cytochalasin B-treated nuclei was similarly elliptical (Figure 7B), although the trend of reduction in the nuclear volume was not significant (Figure 7C). Since the disruption of cytoplasmic actin filaments can lead to nuclear shrinkage (Gupta et al., 2012; Mazumder and Shivashankar, 2010), to further verify the involvement of the intranuclear F-actin in chromatin spreading and nuclear expansion, we utilized the 'mCherry2XNLS-P3D-actin-62R1-pmCherry' (Belin et al., 2015) plasmid expressing nuclear-targeted non-polymerizing R62D mutant of actin (Baarlink et al., 2017; Belin et al., 2015) (Figure 8A). The plasmid expressing Flag-NLS-actinWT was used as a control. To overcome the low transduction and transfection efficiency in primary Th cells, we performed electroporation of *in vitro-*transcribed mRNA of the plasmids (Zhao et al., 2006) into the 24 h-differentiating Th2 cells at the last 6 h of stimulation. Staining for F-actin clearly demonstrated that the inhibition of nuclear actin polymerization reduced chromatin spreading (Figure 8A) and nuclear expansion (Figures 8B and S8) in the 24 h-differentiating Th2 cells. In summary, the methyltransferase activity of Ezh2 is required for the induction of intranuclear F-actin-driving chromatin spreading and nuclear growth in differentiating Th cells.

## Discussion

Actin is an integral component of the cytoplasmic cytoskeleton that regulates critical cellular functions including morphogenesis, migration, membrane transport, and cell-to-cell communication. The presence of actin in the nucleus has long been controversial, however, actin and actin related proteins were recognized finally as regulators of nuclear activities such as transcription and chromatin remodeling (Fischer et al., 1998; Kapoor et al., 2013; Kristo et al., 2016; Misu et al., 2017; Sharili et al., 2016; Virtanen and Vartiainen, 2017; Wesolowska and Lenart, 2015). Although the known function of actin relies on its dynamic polymerization, its nuclear activity was mostly related to its functions as a monomer. Recently, several studies demonstrated a transient nuclear actin polymerization (Belin et al., 2013; Moore and Vartiainen, 2017; Muehlich et al., 2016; Plessner and Grosse, 2019) involving in transcription (Plessner et al., 2015; Tsopoulidis et al., 2019), homology-directed DNA break repair (Caridi et al., 2018; Evdokimova et al., 2018; Schrank et al., 2018), DNA replication (Parisis et al., 2017), and chromatin de-condensation during mitotic exit (Baarlink et al., 2017). The underlying mechanisms and especially the way by which the chromatin and F-actin are connected are unclear yet. Our search to understand the dual function of the epigenetic regulator Ezh2 in differentiating Th cells revealed its crucial role in regulating the cross talk between chromatin and F-actin; Ezh2-dependent methyltransferase activity is required for the induction of intranuclear assembly of actin, which in turn choreographs the differentiation-associated chromatin spreading.

The transient induction of nuclear actin polymerization by Ezh2 can explain the ability of the PcG complexes to regulate higher order chromatin structures (Bantignies et al., 2011; Loubiere et al., 2019; Muller and Kassis, 2006; Schwartz and Pirrotta, 2007). More generally, these findings can provide a proof-of-concept for the way by which the epigenetic machinery harnesses the dynamic mechanical force of the intranuclear actin-skeleton to execute chromatin remodeling. This idea is strengthen by the fact that other epigenetic “readers”, “writers” and “erasers” have been discovered with the ability to remodel actin and microtubule filaments in the cytoplasm (“chromatocytoskeletal” activity) (Walker and Burggren, 2020). It is also possible that the globular actin and actin related proteins function as epigenetic signatures that provide F-actin with nucleation spots and determine heritable patterns of gene expression in proliferating cells.

Ezh2 regulates nuclear actin assembly, at least partially, at the post-transcriptional level, although actin-coding genes were also positively regulated by Ezh2. Post-transcriptional regulation of F-actin is in accordance with the known cytoplasmic activity of Ezh2. In the cytoplasm, Ezh2 controls dendritic cells adhesion and migration through a direct methylation of talin, a key regulatory molecule in cell migration (Gunawan et al., 2015; Venkatesan et al., 2018). Methylation of talin, with Vav as an adaptor molecule, disrupts the binding of talin and F-actin and thereby promotes the adhesion turnover. Phosphorylation of Ezh2 potentiates its binding also to vinculin and other cytoskeletal regulators of cell migration (Anwar et al., 2018). Moreover, Ezh2 directly methylates cytoplasmic alpha-actin to promote cortex cytoskeleton formation in vascular smooth muscle cells (Chen et al., 2017)**.** Indeed, besides interacting with various actin-binding proteins, proper actin function is regulated by post-translational modifications, including methylation, ubiquitination, phosphorylation, and acetylation that contribute to filament formation and stability (Varland et al., 2019). Further study is required to elucidate the nuclear targets of Ezh2 among actin proteins, actin regulators, and actin nucleation factors under different physiological stimuli.

Ezh2 and Vav1 were separately associated with the chromatin fibers, as well as with the actin filaments, and therefore the interaction between Ezh2 and Vav1 is not monogamic. However, Ezh2 and Vav1 were found together at certain promoters in a Th-lineage specific manner. Furthermore, Ezh2 and Vav1 were colocalized at chromatin-F-actin intersections. It remains to be clarified whether and how Ezh2 and potentially other epigenetic regulators, as well as sequence-specific transcription factors, tether Vav1 and additional proteins of the actin apparatus to the chromatin. And most importantly, whether their binding sites determine lineage-specific chromatin spreading. Although our methodology cannot ultimately confirm, the actin filaments seem to be wider than a single actin filament of 7 nm width. It is possible that the heavy duty of spreading chromatin in differentiating cells requires actin bundles, whereas thinner actin filaments regulate more local events such as the interactions between regulatory elements involving in ongoing transcription. In summary, our results altogether strongly suggest that the ability to remodel nuclear cytoskeletal filaments is a crucial skill of the epigenetic machinery.

### Limitation of study

Imaging of the dynamic nuclear F-actin structure, and possibly more rigorous fixation methods, are required to reflect the entire involvement of actin filaments in chromatin reorganization and, as we hypothesize, in transcriptional regulation.

## STAR★ Methods

### Key resources table

**Table:** 

REAGENT or RESOURCE	SOURCE	IDENTIFIER
**Antibodies**		

Ezh2 pAb anti-rabbit	Actif Motif	RRID#AB_2793716
Vav1 (c-14) pAb anti-rabbit	Santa CRUZ	cat#sc-132
WASp (F-8) mAb anti-mouse	Santa Cruz	RRID:AB_10842168
Ezh2 mAb anti-mouse	Cell signaling	cat#3147S
Biotin-XX Phalloidin	Thermo Fisher	cat#B7474
DDB1 pAb anti-goat	Abcam	cat#ab9194
SIR-actin	SPIROCHROME	CY-SC001
Alexa Fluor 488 anti-mouse	Jackson Immuno	RRID#AB_2338840
Cy3 – streptavidin	Invitrogen	cat#434315
Cy5 anti-rat	Jackson Immuno	RRID#AB_2340672
Alexa Fluor 488 anti-rabbit	Jackson Immuno	RRID#AB_2313584
Mounting DAPI fluoromount	SouthernBiotech	cat#0100-20
IMMU-Mount	Thermo scientific	cat#9990402
Alexa Fluor 594 anti-rabbit	Jackson Immuno	RRID#AB_2340621
Cy5 anti-rabbit	Jackson Immuno	RRID#AB_2340607
Vav1 (H-211) pAb anti-rabbit	Santa Cruz	cat#sc7206
Alexa Fluor 647 anti-mouse CD3ε	BioLegend	cat#100322
Hoechst	ThermoFisher	cat#62249

**Experimental Models: Organisms/Strains**		

Mouse: Balb/c	Harlan Biotech	
Mouse: C57BL/6NTac-Ezh2	EMMA consortium	

**Recombinant DNA**		

'mCherry2XNLS-P3D-actin-62R1-pmCherry	https://www.addgene.org/58477/	Addgene cat#58477
Flag-NLS-actinWT	https://www.addgene.org/58467/	Addgene cat#58467

**Software and Algorithms**		

Huygens		
Bowtie		
Partek™		
Seqmonk	www.bioinformatics.babraham.ac.uk/projects/seqmonk/	
meme-chip		
Tomtom	meme-suite online	
galaxy	usegalaxy.org	
GraphPad Prism 6		
ImageJ		
IMARIS 9.2/9.5		
LasX analysis software		

**Other**		

RNA-seq raw data	https://www.ebi.ac.uk/fg/annotare/login/	E-MTAB9523
ChIP-seq raw data	https://www.ebi.ac.uk/fg/annotare/login/	E-MTAB9594 E-MTAB9524

### Resource availability

#### Lead contact

Further information and requests for resources and reagents should be directed to and will be fulfilled by the lead contact, Dr. Orly Avni (Orly.Avni@biu.ac.il).

#### Materials availability

• Plasmids in this study were bought from Addgene, 'mCherry2XNLS-P3D-actin-62R1-pmCherry’ and ‘Flag-NLS-actinWT’, catalog numbers are 58477 and 58467, respectively.
• This study did not generate new unique reagents.

### Expiramental model and subject details

#### Mice

4-5-week-old either females or males BALB/c mice were purchased from Harlan Biotech (Jerusalem, Israel). C57BL/6NTac-Ezh2<tm1a(EUCOMM)Wtsi (*Ezh2* conditional KO mice) were obtained from EMMA consortium. These mice were crossed with rosa26-creERT2 mice for total body tamoxifen-induced *Ezh2* deficiency. The mice maintained under pathogen-free conditions at the animal facility/Faculty of Medicine/Bar-Ilan university, Safed. The studies have been reviewed and approved by the Bar-Ilan institutional ethic committee.

#### In vitro Th cell differentiation

This protocol was carried out essentially as described (Avni et al., 2002). Briefly, CD4^+^ T cells were purified from spleen and lymph nodes of 4-5-week-old mice using magnetic beads. For Th differentiation, naïve cells were stimulated with 1μg/ml anti-CD3ε Ab (TCR stimulation; 145.2C11, hybridoma supernatant) and 1 μg/ml of anti-CD28 Ab (co-stimulation; 37.51, BioLegend) in Dulbecco's modified Eagle's medium (DMEM) supplemented with 10% fetal calf serum, L-glutamine, penicillin-streptomycin, nonessential amino acids, sodium pyruvate, vitamins, HEPES and 2-mercaptoethanol, in a flask coated with 0.1 mg/ml of goat anti-hamster Ab (ICN). For Th1 differentiation, the cells were stimulated in the presence of 10ng/ml of recombinant mouse IL-12 (R&D Systems) and 10μg/ml purified anti-IL-4 Ab (11B11). For Th2 differentiation, the cells were stimulated in the presence of 1000U/ml of mouse IL-4 (added as a supernatant of the 13L6 cell line), 5μg/ml anti-IFNγ Ab (XMG1.2) and 3μg/ml anti-IL-12 Ab (C178).

#### Ezh2 knocked-out Th cells

Tamoxifen (200μg/1gr mouse) or filtered corn oil (sigma), as control, were injected daily IP to 4-5-week-old conditional Ezh2-knock-out mice for 5 days before the purification of the naïve Th cells.

### Method details

#### Inhibitors

10μM UNC1999 (Cayman Chemical) was used to inhibit Ezh2 and 10μM cytochalasin B (Sigma) was used to inhibit actin polymerization. 'mCherry2XNLS-P3D-actin-62R1-pmCherry' (addgene), which expresses nuclear-targeted non-polymerizing R62D mutant of human actin, with an mCherry expressing reporter, was used to specifically inhibit the polymerization of nuclear actin. The plasmid Flag-NLS-actinWT was used as a control (Baarlink et al., 2017; Belin et al., 2015). The plasmids were transcribed *in-vitro* and the mRNAs were electroporated (300V, 1ms) into 18hr-differentiated Th2 cells 6hr before harvesting.

#### Chromatin immuno-precipitation sequencing (ChIP-Seq)

Cells (1x10^6^) were cross-linked at RT for 10 min on ice by adding 1% formaldehyde solution directly to the media. Following incubation, glycine was added to a final concentration of 0.125M. The cells were then washed twice with ice cold PBS, and incubated in Tris-DTT buffer for 15 min at 30°C followed by sequential wash with PBS, Buffer I (0.25% Triton X-100, 10mM EDTA, 0.5mM EGTA, 10mM HEPES pH-6.5) and Buffer II (200mM NaCl, 1mM EDTA, 0.5mM EGTA, 10mM HEPES pH-6.5), and then re-suspended in 0.5ml of lysis buffer (0.5ml of lysis buffer - 1% SDS, 10 mM EDTA, 50 mM Tris-HCl pH 8.1, 1× protease inhibitor cocktail) and sonicated 3 times with 4 min interval at 4°C or by Covaris device for 8 min. The samples were centrifuged at 14,000 rpm at 13°C for 10 min and the supernatant was diluted with equal amount of dilution buffer. Aliquots containing 0.5-1x10^6^ cells stored frozen at -80°C.

Frozen chromatin samples were immunoprecipitated overnight at 4°C with antibodies and 20μl magnetic beads (Magna a+g beads Millipore). Precipitates were washed sequentially for 5 min each in TSE I (0.1% SDS, 1% Triton X-100, 2mM EDTA, 20mM Tris-HCl pH 8.1, 150mM NaCl), TSE II (0.1% SDS, 1% Triton X-100, 2mM EDTA, 20 mM Tris-HCl pH 8.1, 500 mM NaCl), and Buffer III (0.25M LiCl, 1% NP-40, 1% Deoxycholate, 1mM EDTA, 10mM Tris HCl pH-8.1), and two times with 1ml of TE buffer (10mM Tris-HCl pH-8.1, 1mM EDTA) using magnetic plate, and extracted with 50μl of extraction buffer (0.5%SDS, 300mM NaCl, 5mM EDTA, 10mM Tris-HCl pH8.1). After short vortex, the elutions were heated at 65°C for 2hr in the presence of Proteinase K (10μg/μl) to reverse the cross linking. 50μl of the supernatant was separated using the magnetic plate and transferred into new tubes. 120μl of AmpureBeads (Beckman Coulter) were added to the supernatant tubes and incubated at room temperature for 5 min. Using magnetic plate the samples were washed with EtOH 70%, and the DNA was eluted with 110μl of UPW (Biological Industries) for further DNA Library preparation and sequencing by illumine high-seq (Faculty of Medicine Bar-Ilan university).

#### ChIP-seq analysis

Fastq files were aligned using bowtie in Partek™ environment. Peaks were detected using MACS in Seqmonk (https://www.bioinformatics.babraham.ac.uk/projects/seqmonk/) software with p Value<0.0001 threshold. Gene annotations and graphical output were generated using either Partek or seqmonk software. Intersection of peaks conducted using bedtools intersect intervals algorithm in usegalaxy.org online tools. Motif finding was conducted using meme-chip and annotated using Tomtom in meme-suite online.

#### Total RNA extraction and RNA-seq

RNA was extracted (Norgen), mRNA was purified (NEBNext® Poly(A) mRNA Magnetic Isolation Module; New England biotechnologies) and cDNA library was constructed (NEBNext® Ultra™ RNA Library Prep Kit for Illumina®). Each sample was indexed, multiplexed and sequenced in the genomic center (Faculty of Medicine Bar-Ilan university). The data was aligned using bowtie and further analyzed using seq-monk (https://www.bioinformatics.babraham.ac.uk/projects/seqmonk/) for differential expression.

#### Proteomics

Cells (5x10^6^) were collected and transferred to 1.5 ml tubes (on ice) and centrifuged for 10 sec at 6000rpm. The supernatant was discarded and 900μl of HL buffer (10mM HEPES-KOH, pH7.5, 10mM KCl, 3mM MgCl2, 0.05% NP-40, 1mM EDTA, pH8) was added for 30 min (on ice). Nuclei were centrifuged in 3000rpm for 5 min, and supernatant was discarded. Resuspension of the pellet in 900μl HL buffer and centrifugation of the nuclei in 3000rpm for 5 mins. Supernatant was discarded and 900μl of 1xGSLB buffer (50mM HEPES-KOH, pH7.9, 250mM KCl, 0.1% NP-40, 0.1mM EDTA pH8, 0.1mM EGTA pH8, 10% glycerol) was added and incubated for 30 min (on ice). Nuclei were centrifuged in full-speed for 10 min at 4°C and extracted protein were collected. Mass spectrometry was conducted at the Smoler proteomic center at the Technion/Israel.

#### Staining

Cells were centrifuged at 5000rpm at 4°C for 3 min. 3X10^6^ cells were transferred into Eppendorf tubes with 100μl PBS buffer and centrifuged at 3000rpm at 4°C for 3 min fixed with 100μl of 3.7 % paraformaldehyde (PFA) for 10 min at room temperature, and rinsed twice with washing buffer PBS. Subsequently, the cells were permeabilized with 0.3% TritonX (diluted in PBS) for 5 min, followed by rinsing twice with PBS buffer. Staining was done with 2 μg/ml of Ab: WASp mouse mAb (f-8, Santa Cruz Biotechnology); Vav1 rabbit pAb or mAb (c14 or H211, respectively. Santa Cruz Biotechnology); Ezh2 rabbit pAb (Active motif) or Ezh2 mouse mAb (Cell Signaling – only as indicated for Figures 3A, 3H, 4, and S3A, to be able to co-stain Ezh2 and Vav1, and DDB1 rat polyclonal Ab (1 μg/ml; ab9194, abcam). Phalloidin-Biotin (1μM; Thermo Fisher Scientific) or SirActin (1μΜ; Spirochrome – only as indicated for Figure S1D) were applied for F-actin visualization. Abs were diluted in donkey serum and samples were incubated for 1 hr. Then the samples were rinsed 3 times with PBS washing buffer, and further incubated with second antibodies, which were diluted in donkey serum for 1hr: anti-DyLight 488 anti-mouse (2.5 μg/ml)/ streptavidin Cy3 (2 μg/ml)/ anti-DyLight 488 anti-rabbit/ Alexa Fluor® 594, anti-rabbit (2.5 μg/ml), cy5 anti-rat or Cy5 anti-rabbit (1.5 μg/ml). The samples were rinsed twice with PBS buffer. 100 μl PBS was added to the tubes following 1.5 μl of Hoechst staining and the liquid was transferred to live cell tubes (ibidi). As control, a secondary Ab was used only.

For cytospin samples, the cells were centrifuged at 800 RPM for 3 min on cytospin slides (Tharmac Cellspin)*,* stained as described above and were mounted with 50 μl of Mounting DAPI fluoromount (SouthernBiotech)/IMMU-Mount (Thermo scientific).

To maintain cellular orientation (staining in the context of CD3 stimulation), Chamber Slide System (ThermoFisher) staining protocol was applied. The growth media and wells were removed and the cells were stained as described above. The sample were mounted with 50μl of Mounting DAPI fluoromount (SouthernBiotech)/IMMU-Mount (Thermo scientific).

### Quantification and statistical analysis

#### Image processing and quantification

To visualize and evaluate chromatin spreading, the confocal super-resolution microscopy was applied. For visualization or quantification, the Z-stacks specific signals were generated into surface/protein/filamentous application and reconstructed a 3D image. For nuclear-specific analysis, images were acquired with complete z-stacks and the signal obtained by Hoechst was analyzed by surface application and respective volumes were measured (μm^3^). To visualize F-actin, images were acquired with complete z-stacks of the signal obtained by phalloidin and were analyzed by surface application with IMARIS 9.2 or 9.5 software. For visualization of Ezh2/Vav1/WASp proteins, images were acquired with complete z-stacks, the signals were analyzed by spots application with IMARIS 9.2 or 9.5 software. For Figures 7, 8, and Figure S1D. The filamentous application was applied, the size of nuclear filaments was defined as 5nm – 35nm, to analyze and visualize the accurate F-actin fibers orientation. To assess colocalization of 2 channels, we applied LasX colocalization tool that carry out a colocalization analysis within an area region of interest (ROI). The colocalization rate is presented by the indicated percentage values and is calculated from the ratio of the requested area out of the total fluorescence signals (the Colocalization area specifies the area of colocalizing fluorescence signals [in μm^2^], out of the area of the image foreground [in μm^2^] that is calculated from the difference between the total area of the image, and the area of the image background (Area Background [μm^2^]). To assess colocalization of 4 channels (only for Figure 4A) we used ImageJ software; the stack image was split into 4 different color channels and automatically threshold was applied to convert each channel to binary. The intersected pixels of each 2 images were combined to find channels of colocalization.

#### Microscopy

Images and Z-stacks were acquired by the Leica Super Resolution (SR) TCS SP8 HyVolution, which merges optical super-resolution (confocal) and computational (deconvolution) super-resolution, using x63 oil objective. Alternatively, as indicated, images were acquired by SR stimulated emission depletion (STED), using x100 oil objective. Deconvolution was performed automatically by Huygens software.

#### Statistical analysis

GraphPad prism version6 was used for two-tailed T-test statistical analysis or non-parametric tests, based on the distribution of the population. Proportion Z-tests and exact Fischer’s test were used to test significant difference in the prevalence of two-sample or three-sample for unrelated sample comparisons. Differences between groups were considered statistically significant when ∗p ≤ 0.05; ∗∗p ≤ 0,01; ∗∗∗p ≤ 0.001; ∗∗∗∗p ≤ 0.0001.

## Data Availability

• ChIP-seq and RNA-seq Raw data have been deposited at ANNOTARE and are publicly available as of the date of publication. Accession numbers are listed in the key resources table. • This paper does not report original code. • Any additional information required to reanalyze the data reported in this paper is available from the lead contact upon request.

## Supplemental information

Document S1. Figures S1–S8

Table S1. Proximal genes (in distance smaller than 5kb from Transcription Start Site) to the Ezh2/Vav1 (exact overlapping) peaks in 24 h-differentiating Th1 and Th2 cells (FDR<0.05). Traces ChIP-seq, related to STAR Methods

Table S2. (a) Genes that are bound by both Ezh2 and Vav1 (in proximity) in the 24 h-differentiating Th1 cells (with FDR<0.05). Traces ChIP-seq, related to STAR Methods. (b) Genes that are bound by both Ezh2 and Vav1 (in proximity) in the 24 h-differentiating Th2 cells (with FDR<0.05). Traces ChIP-seq, related to STAR Methods

Table S3. (a) RNA-seq of differentially expressed genes in the 24 h-differentiating Th1 cells, with or without the presence of Ezh2 inhibitor (UNC1999) (FDR<0.05). Traces RNA-seq, related to STAR Methods. (b) RNA-seq of differentially expressed genes in the 24 h-differentiating Th2 cells, with or without the presence of Ezh2 inhibitor (UNC1999) (FDR<0.05). Traces RNA-seq, related to STAR Methods

Table S4. (a) Combining the Ezh2-ChIP-seq annotated peaks (±5kb from TSS) with the RNA-seq values of differentially expressed genes (FDR<0.05) with or without the presence of Ezh2 inhibitor (UNC1999) of the 24 h-differentiating Th1 cells. Traces ChIP-seq, related to STAR Methods. (b) Combining the Ezh2-ChIP-seq annotated peaks (±5kb from TSS) with the RNA-seq values of differentially expressed genes (FDR<0.05) with or without the presence of Ezh2 inhibitor (UNC1999) of the 24 h-differentiating Th2 cells. Traces ChIP-seq, related to STAR Methods

Table S5. (a) Ezh2/Vav1 peaks (±5kb from TSS) in the 24 h-differentiating Th1 combining the RNA-seq values of differentially expressed genes (FDR<0.05) with or without the presence of Ezh2 (UNC1999) inhibitor. Traces ChIP-seq and RNA-seq, related to STAR Methods. (b) Ezh2/Vav1 peaks (±5kb from TSS) in the 24 h-differentiating Th1 combining the RNA-seq values of differentially expressed genes (FDR<0.05) with or without the presence of F-actin (Cytochalasin B) inhibitor. Traces ChIP-seq and RNA-seq, related to STAR Methods

Table S6. The expression levels of genes (RNA-seq values) that are associated with the Ezh2 and Vav1 bound genes (in proximity) of the 24 h-differentiating Th1 and Th2 cells. ChIP-seq and RNA-seq, related to STAR Methods
