# Supplementary material for: Ezh2 harnesses the intranuclear actin cytoskeleton to remodel chromatin in differentiating Th cells
Source: iScience. 2021 Sep 9;24(10):103093. doi: 10.1016/j.isci.2021.103093 (PMC8479699; doi:10.1016/j.isci.2021.103093)
Supplement: Document S1. Figures S1–S8 [file mmc1.pdf]

iScience, Volume *24*

Supplemental information

Ezh2 harnesses the intranuclear actin cytoskeleton to remodel chromatin in differentiating Th cells

Moran Titelbaum, Boris Brant, Daniel Baumel, Alina Burstein-Willensky, Shira Perez, Yiftah Barsheshet, and Orly Avni

Figure S1

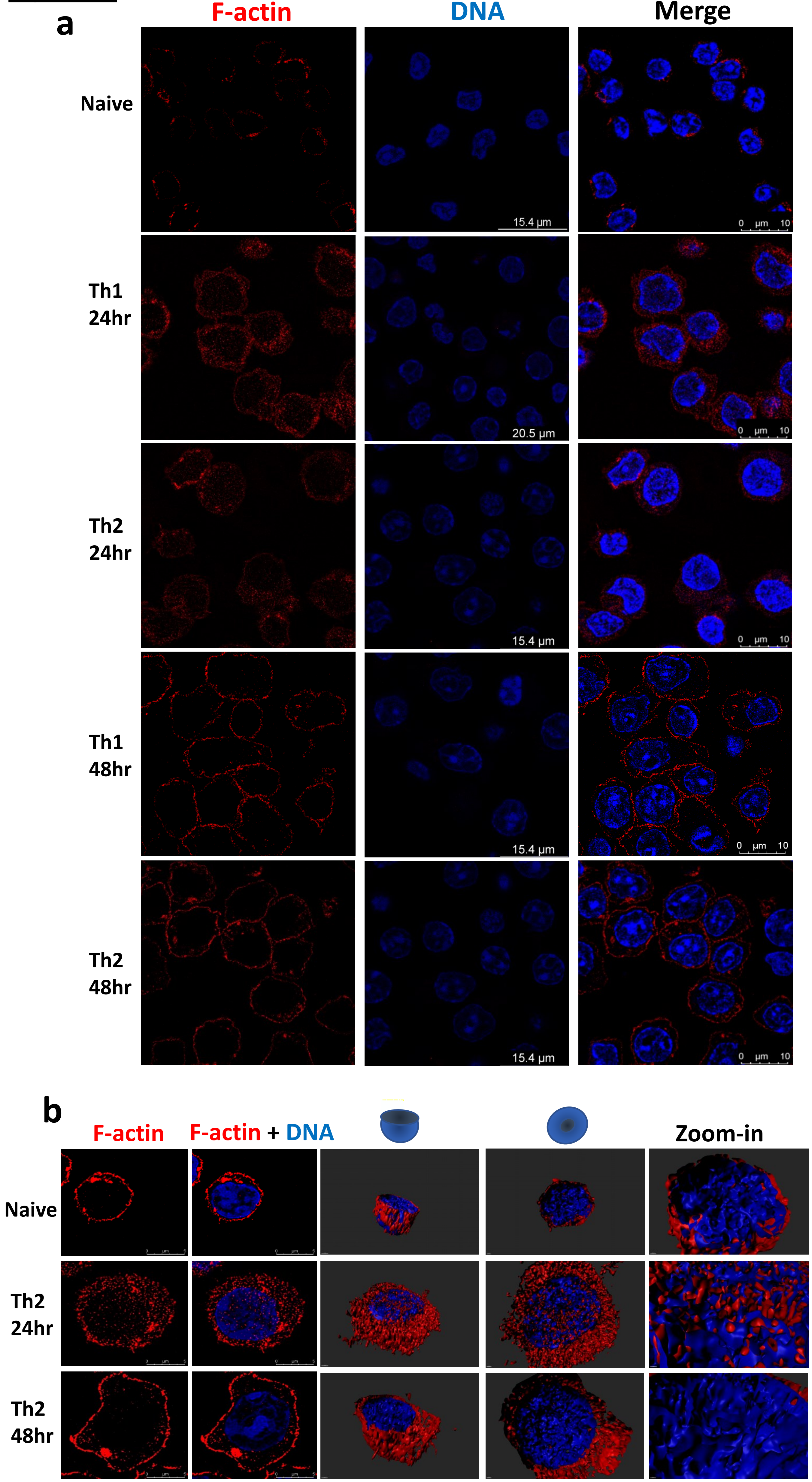
a
F-actin
DNA
Merge
Naive
Th1
24hr
Th2
24hr
Th1
48hr
Th2
48hr
15.4 μm
20.5 μm
0 μm 10
b
F-actin
F-actin + DNA
Zoom-in
Naive
Th2
24hr
Th2
48hr

c

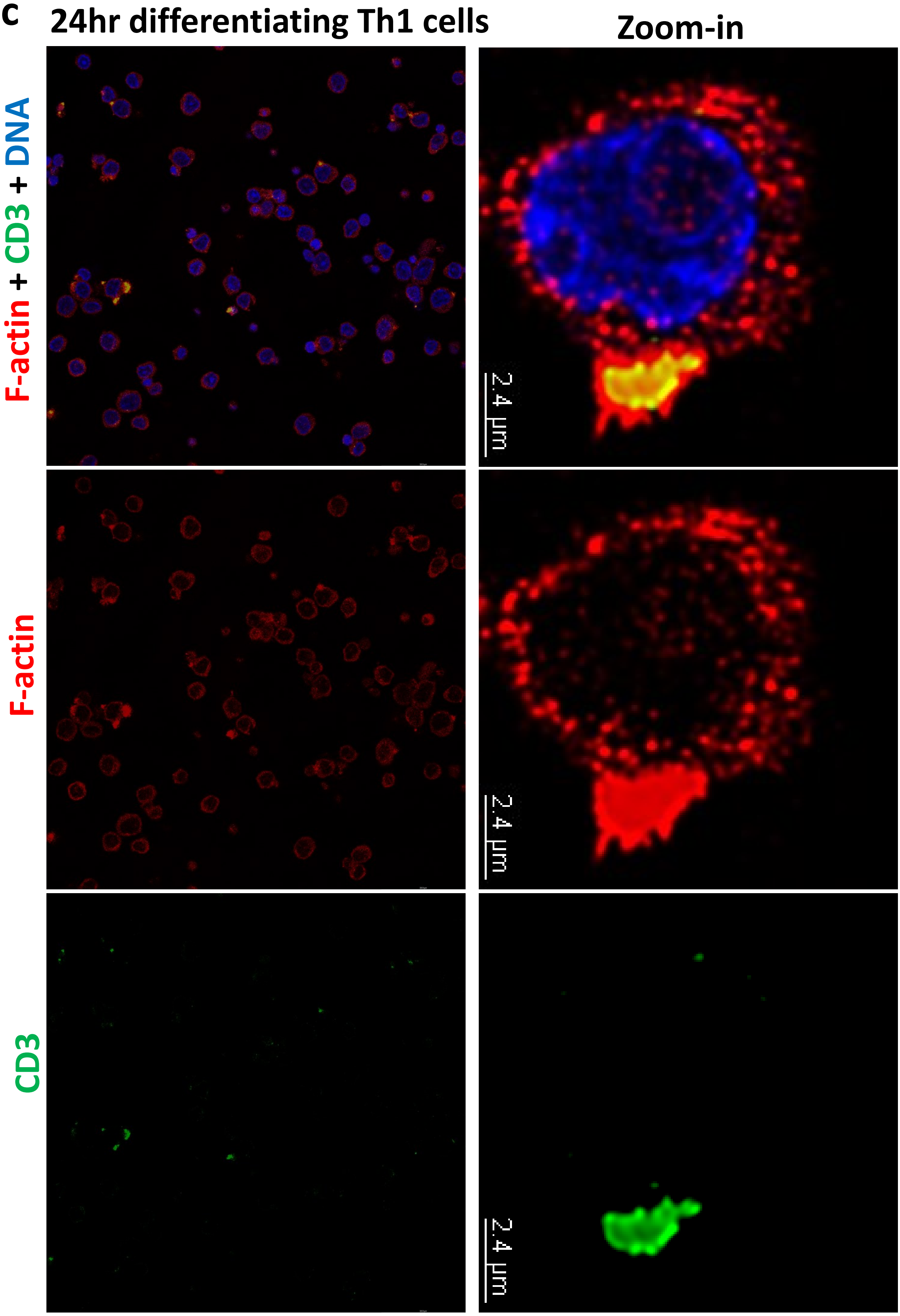
24hr differentiating Th1 cells
Zoom-in
F-actin + CD3 + DNA
F-actin
CD3
2.4 µm
2.4 µm
2.4 µm

d

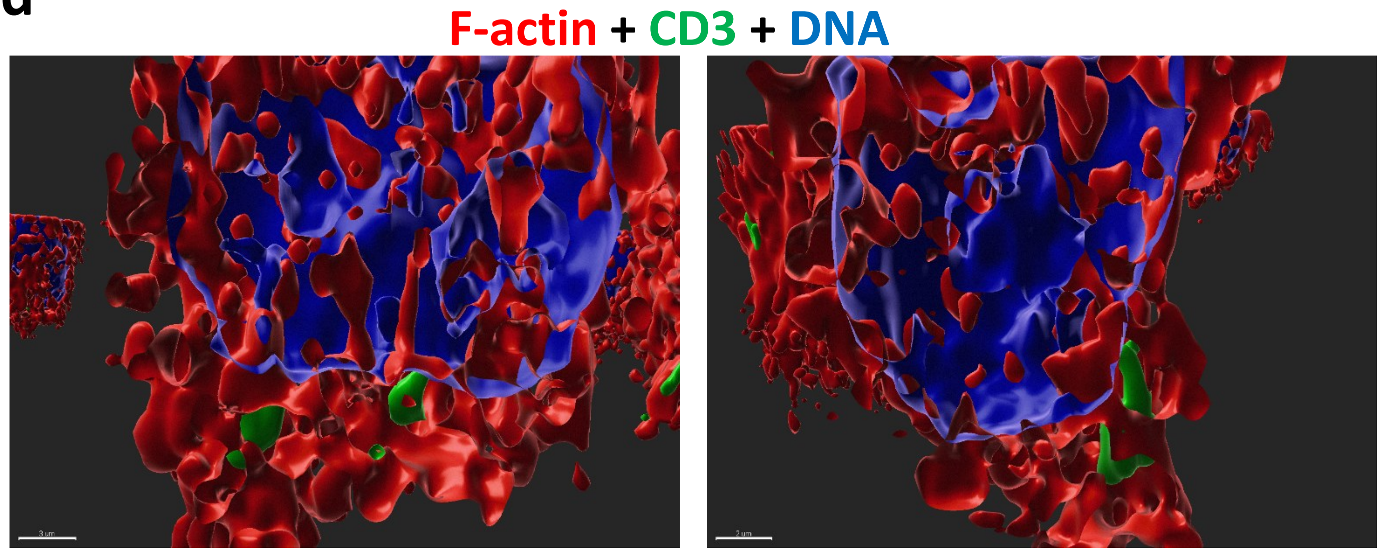
F-actin + CD3 + DNA

e

F-actin + CD3 + DNA

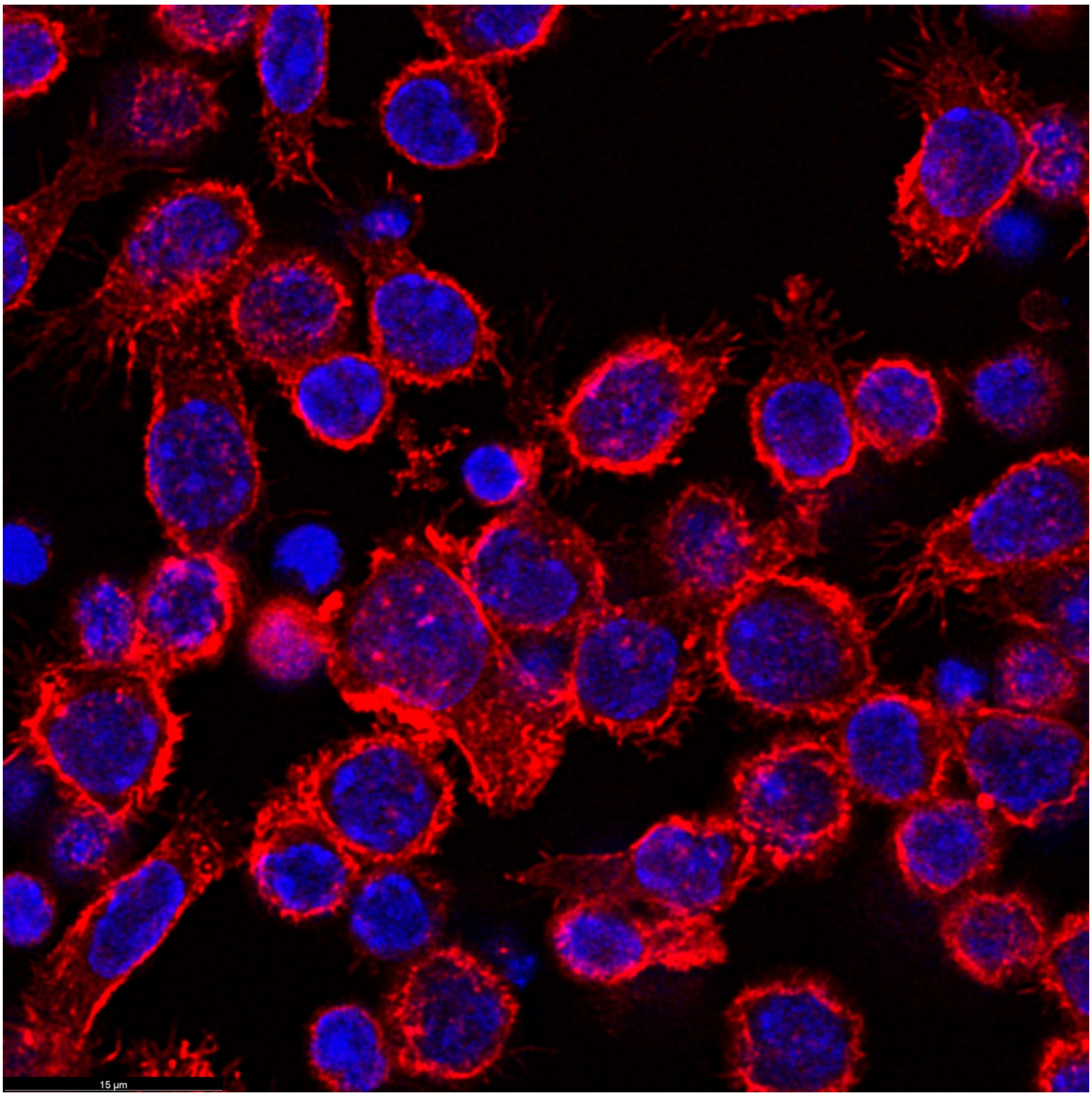

f

F-actin + DNA

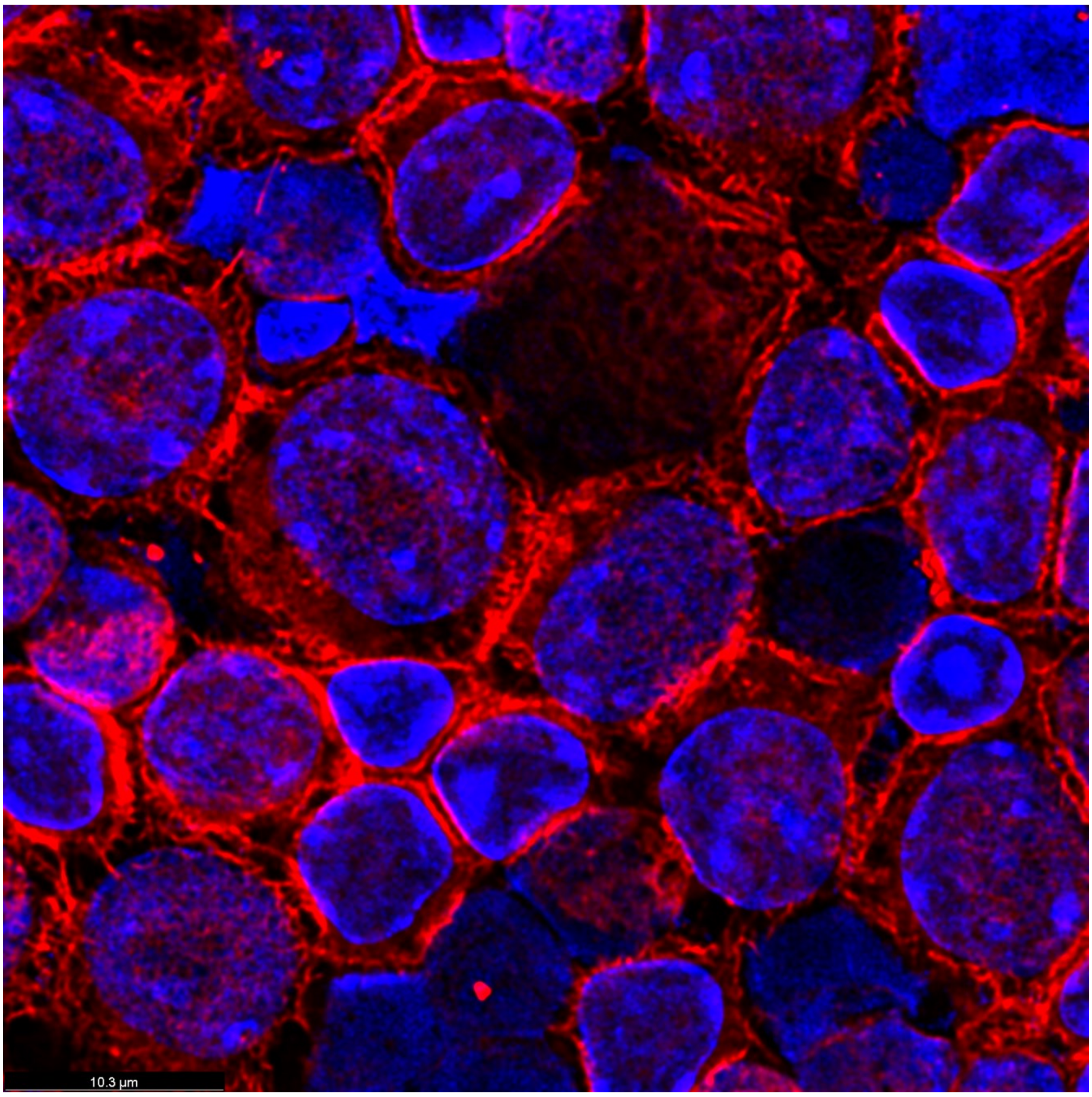

24hr differentiating Th1 cells

g

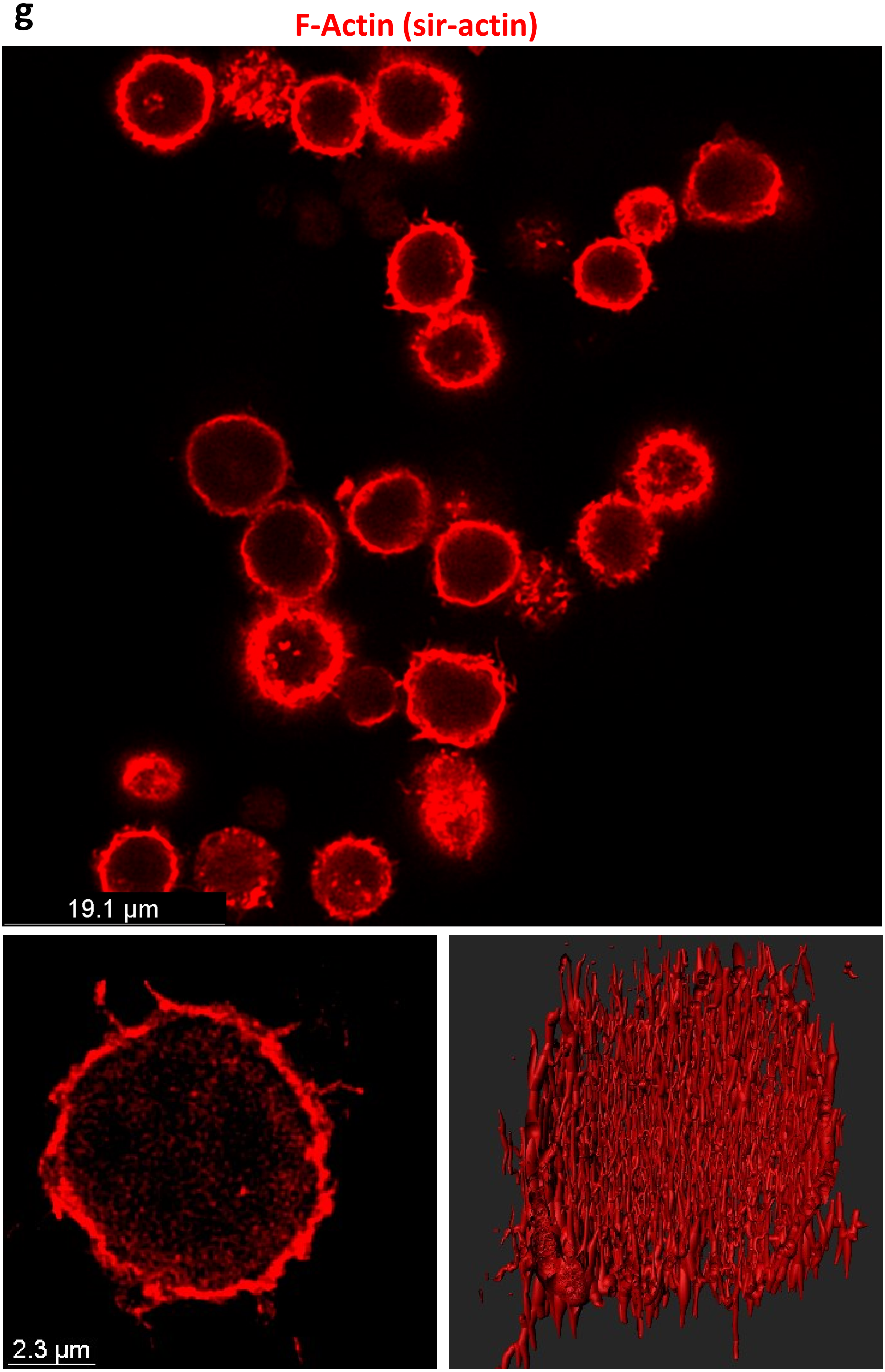

24hr differentiating Th1 cells

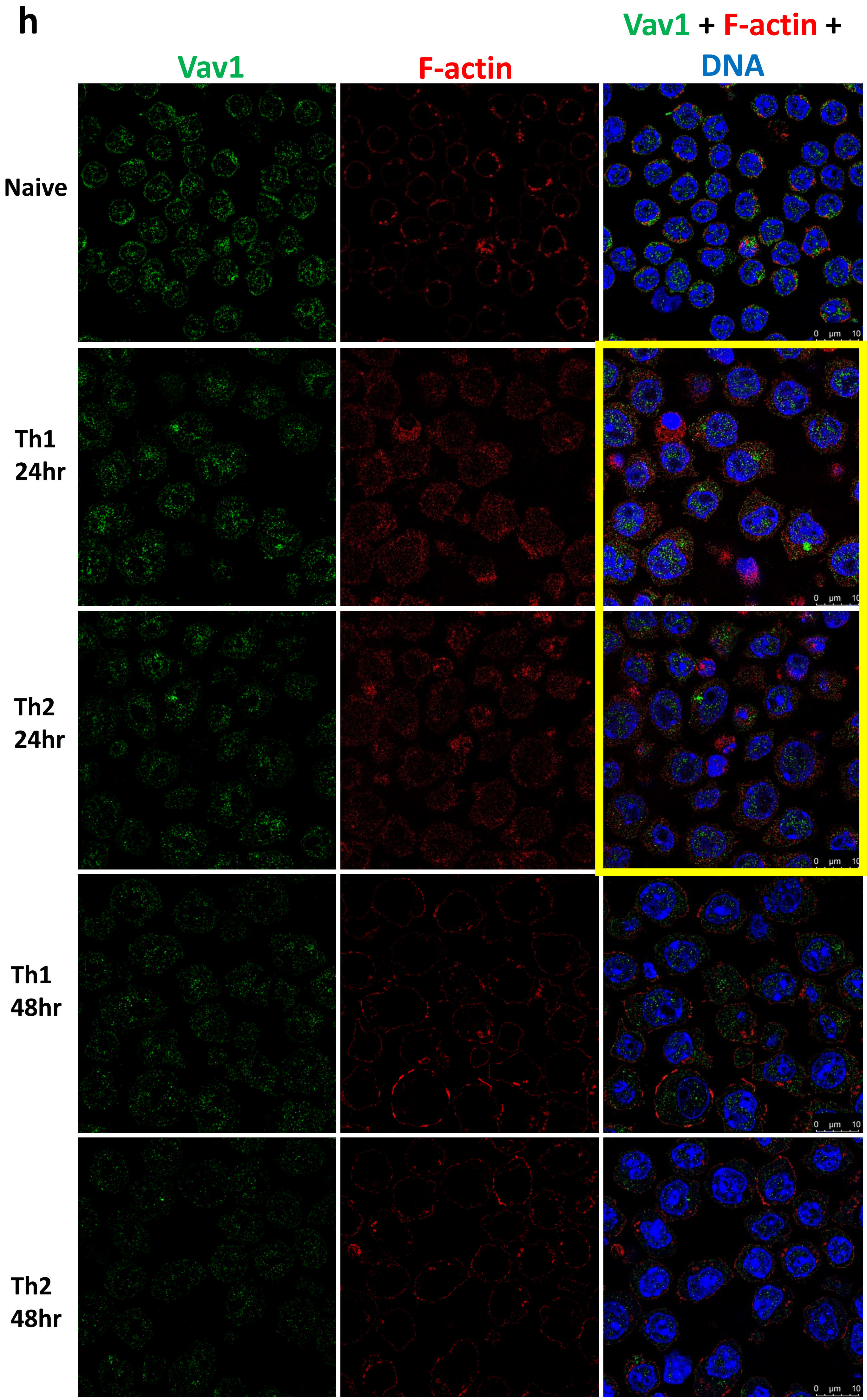

The yellow box is enlarged in the next page

Th1 24hr

Vav1 + F-actin + DNA

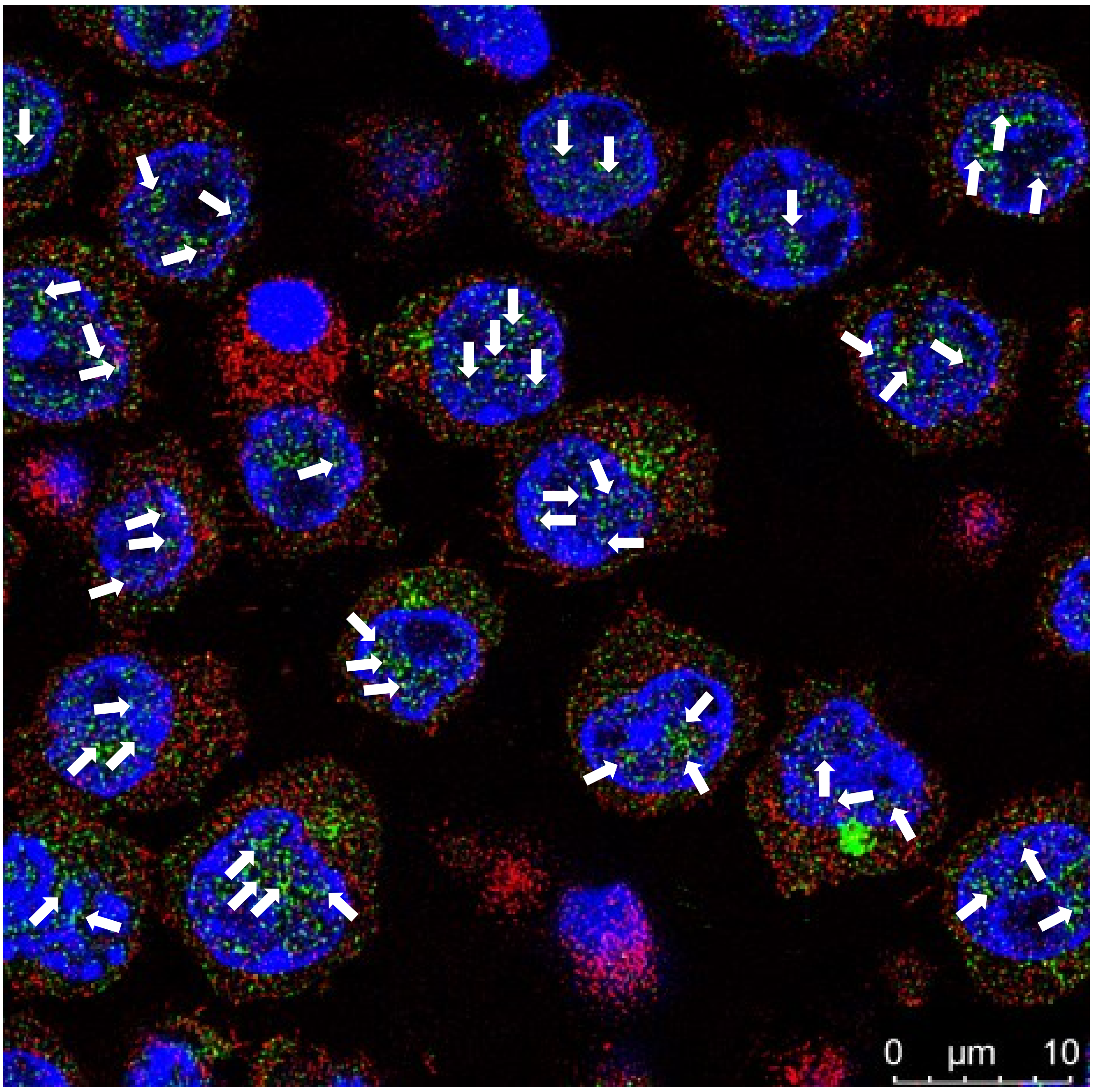

Th2 24hr

Vav1 + F-actin + DNA

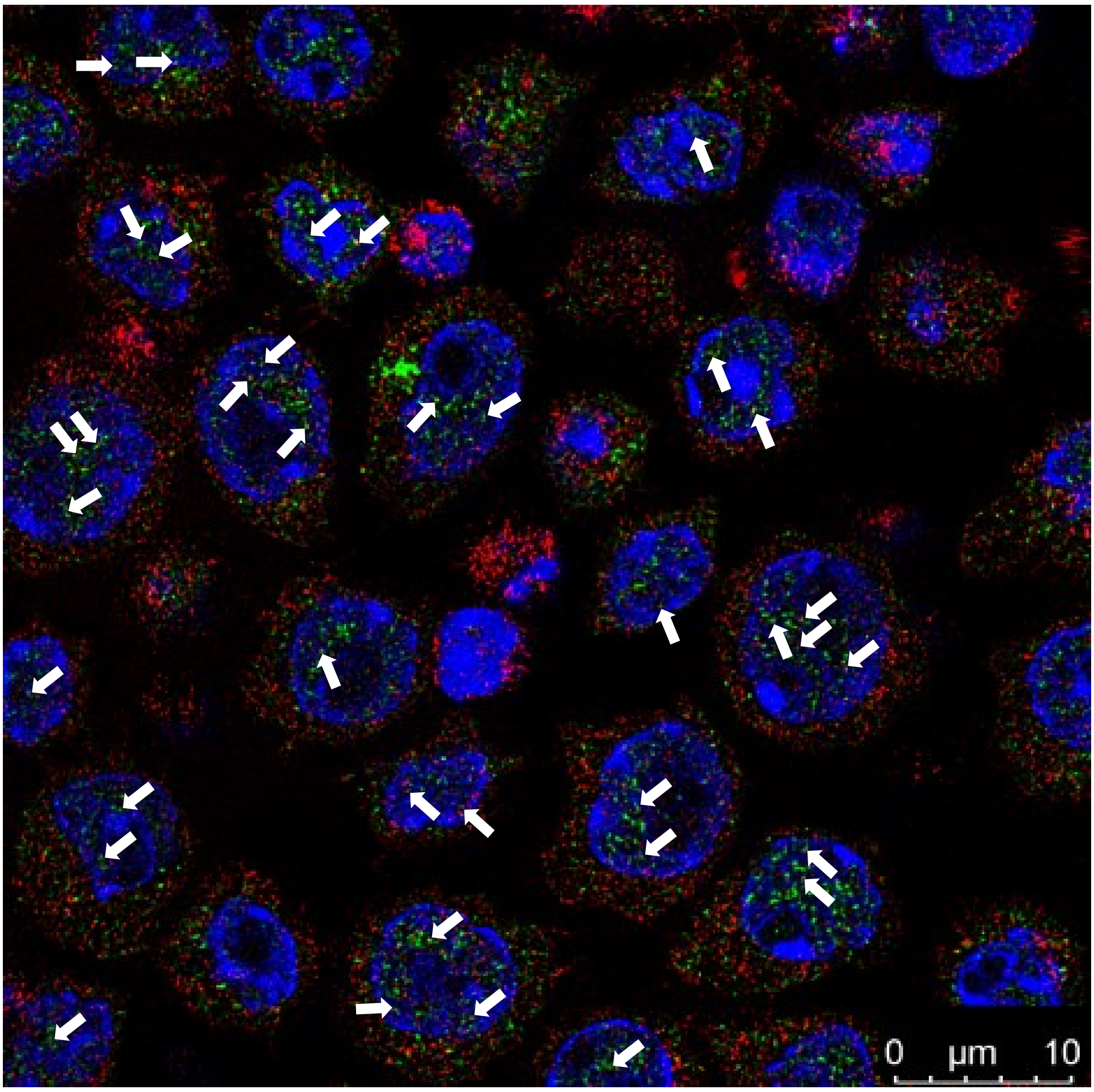

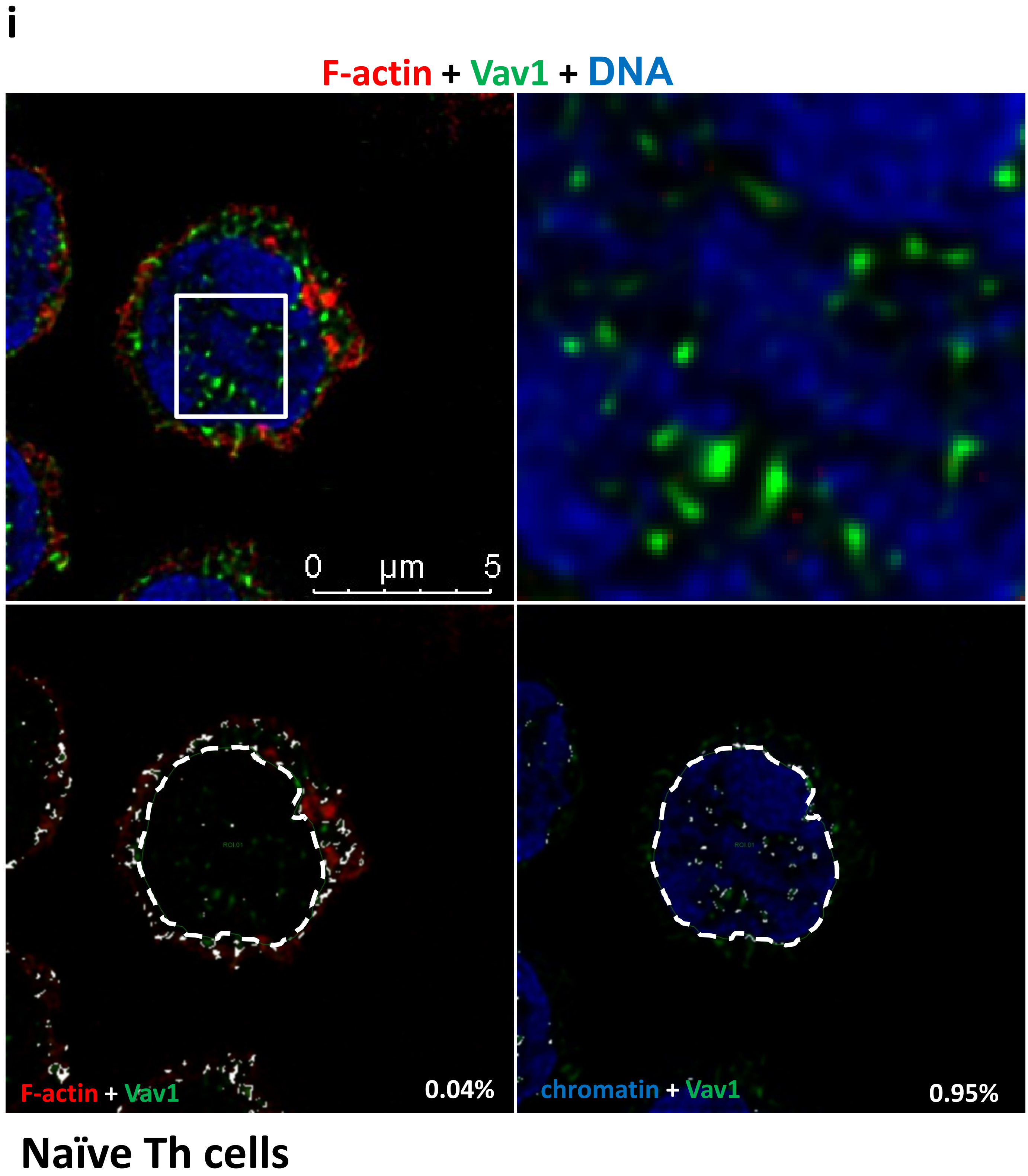
i
F-actin + Vav1 + DNA
0 µm 5
F-actin + Vav1
0.04%
chromatin + Vav1
0.95%
Naïve Th cells

j

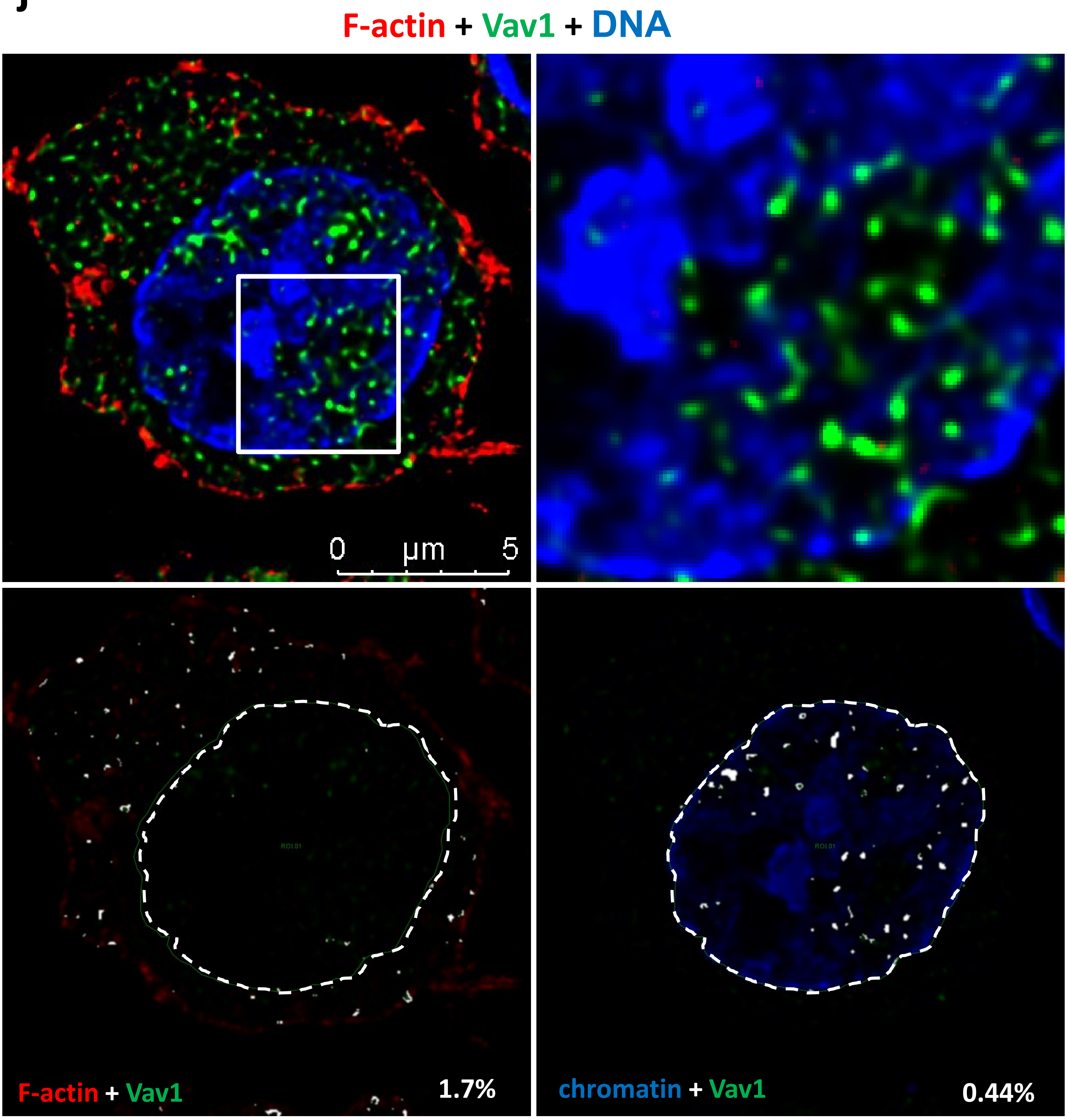

48hr-differentiating Th1 cells

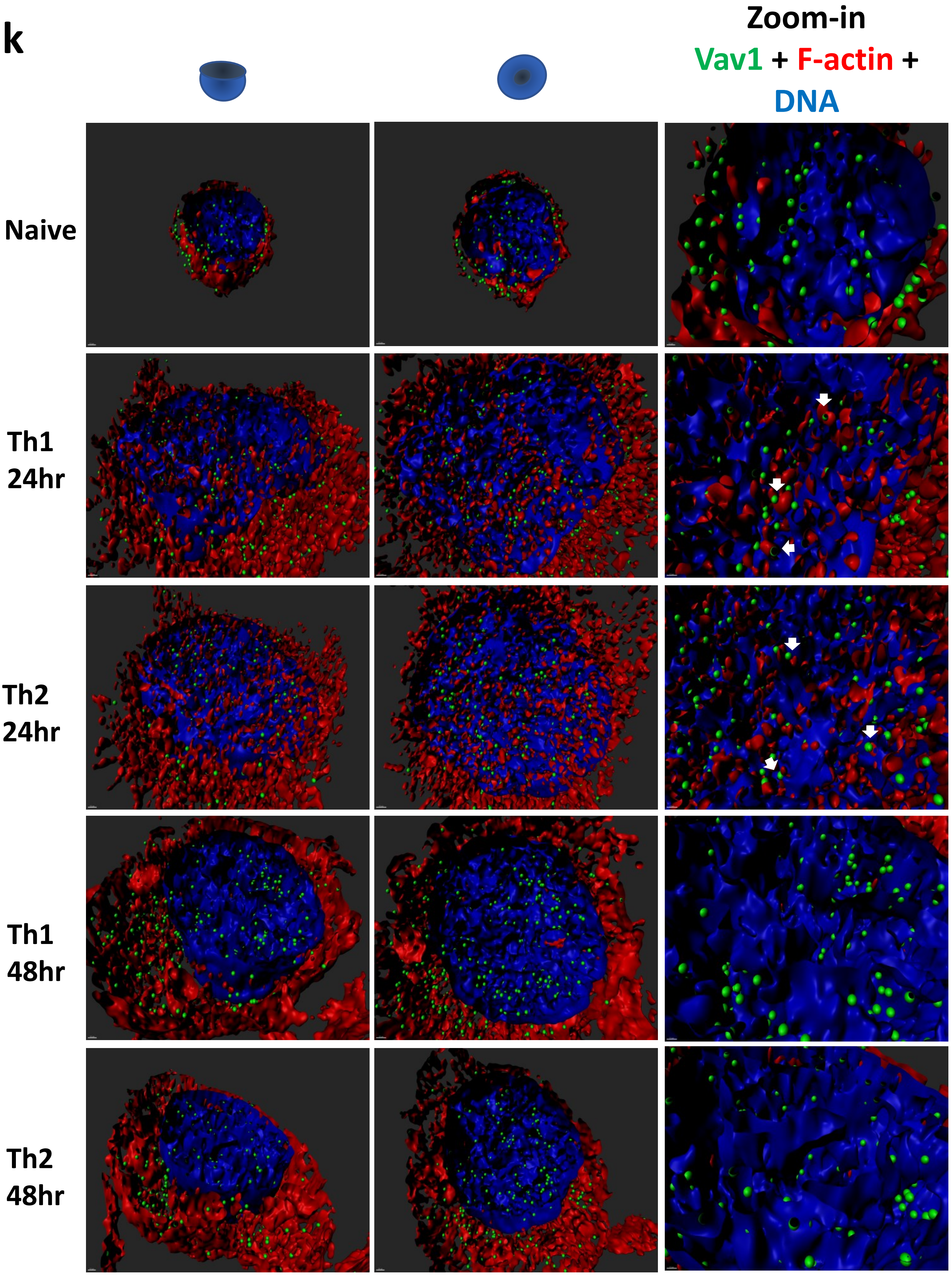
k
Zoom-in
Vav1 + F-actin + DNA
Naive
Th1
24hr
Th2
24hr
Th1
48hr
Th2
48hr

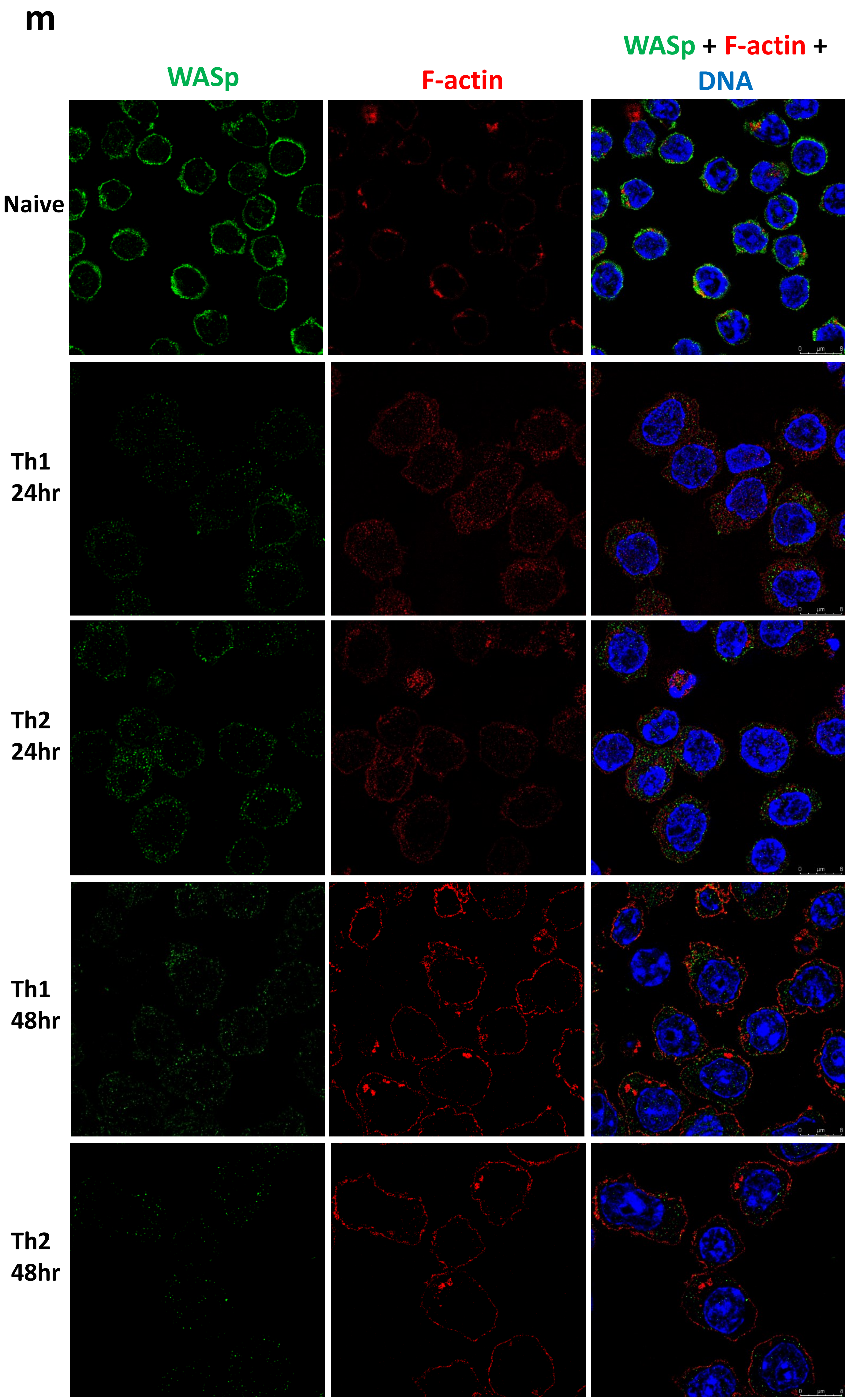
m
WASp
F-actin
WASp + F-actin + DNA
Naive
Th1 24hr
Th2 24hr
Th1 48hr
Th2 48hr

n

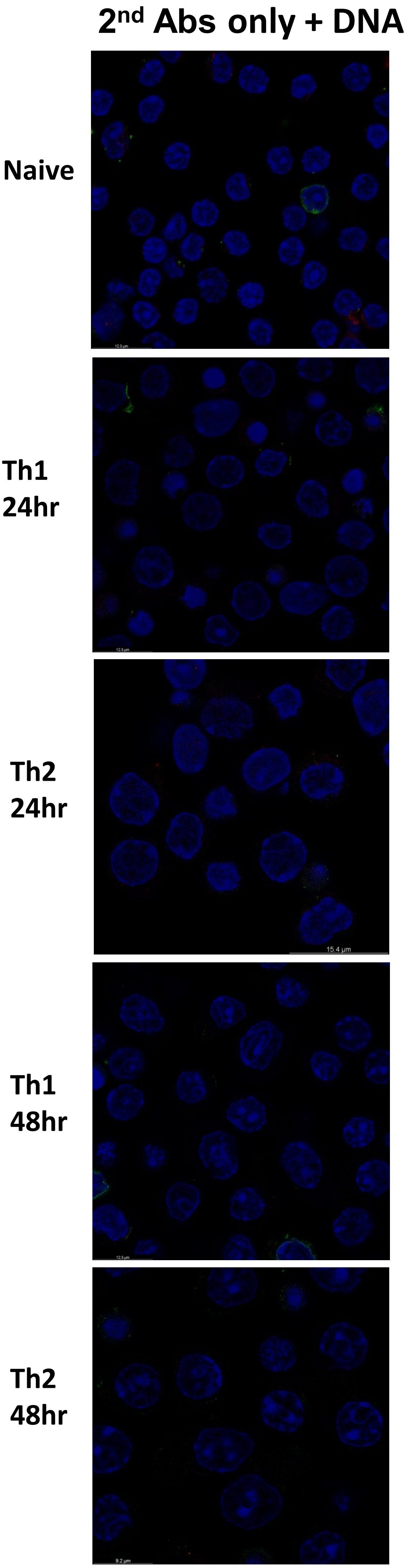

o

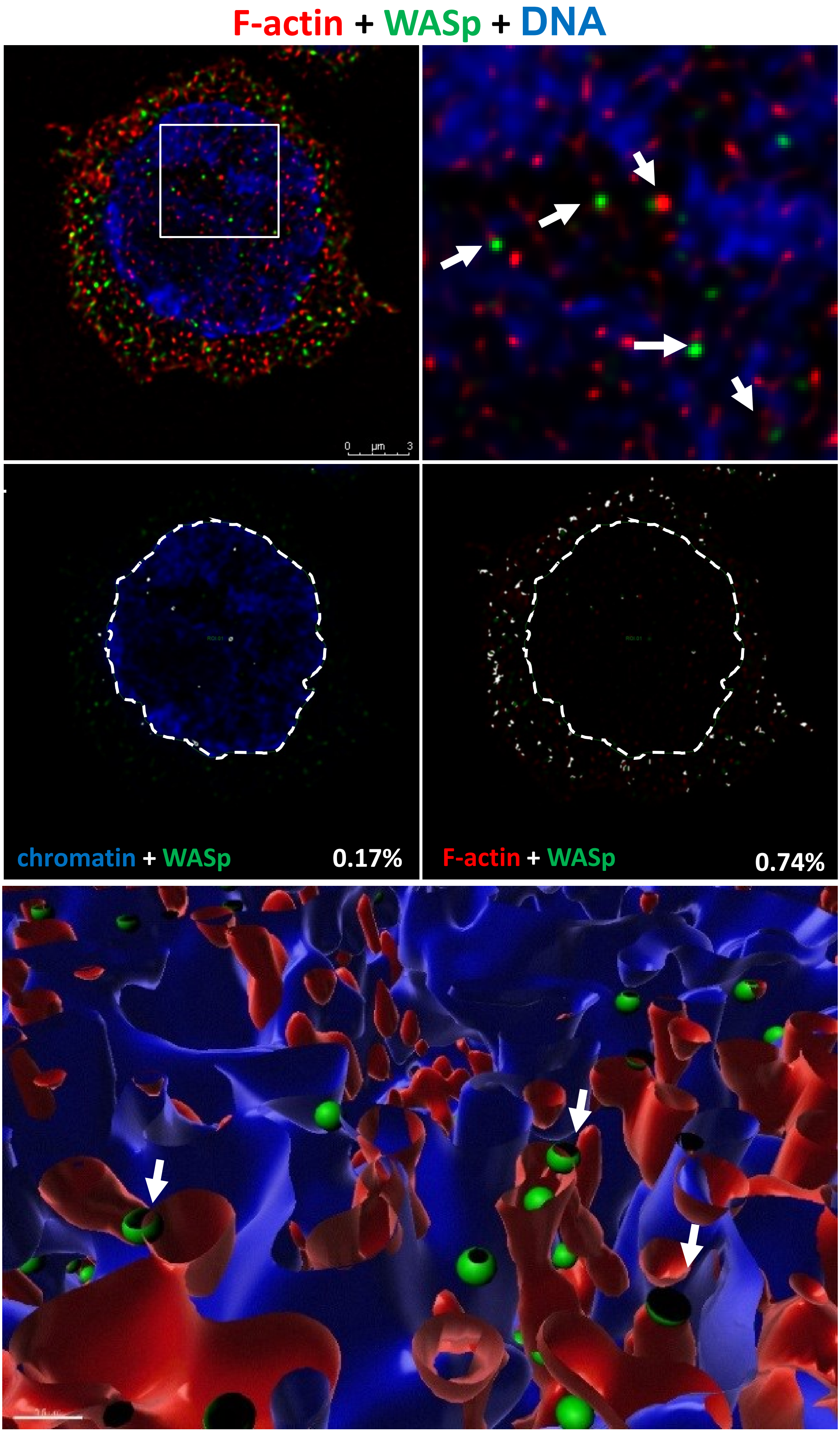

24hr-differentiating Th1 cells

p

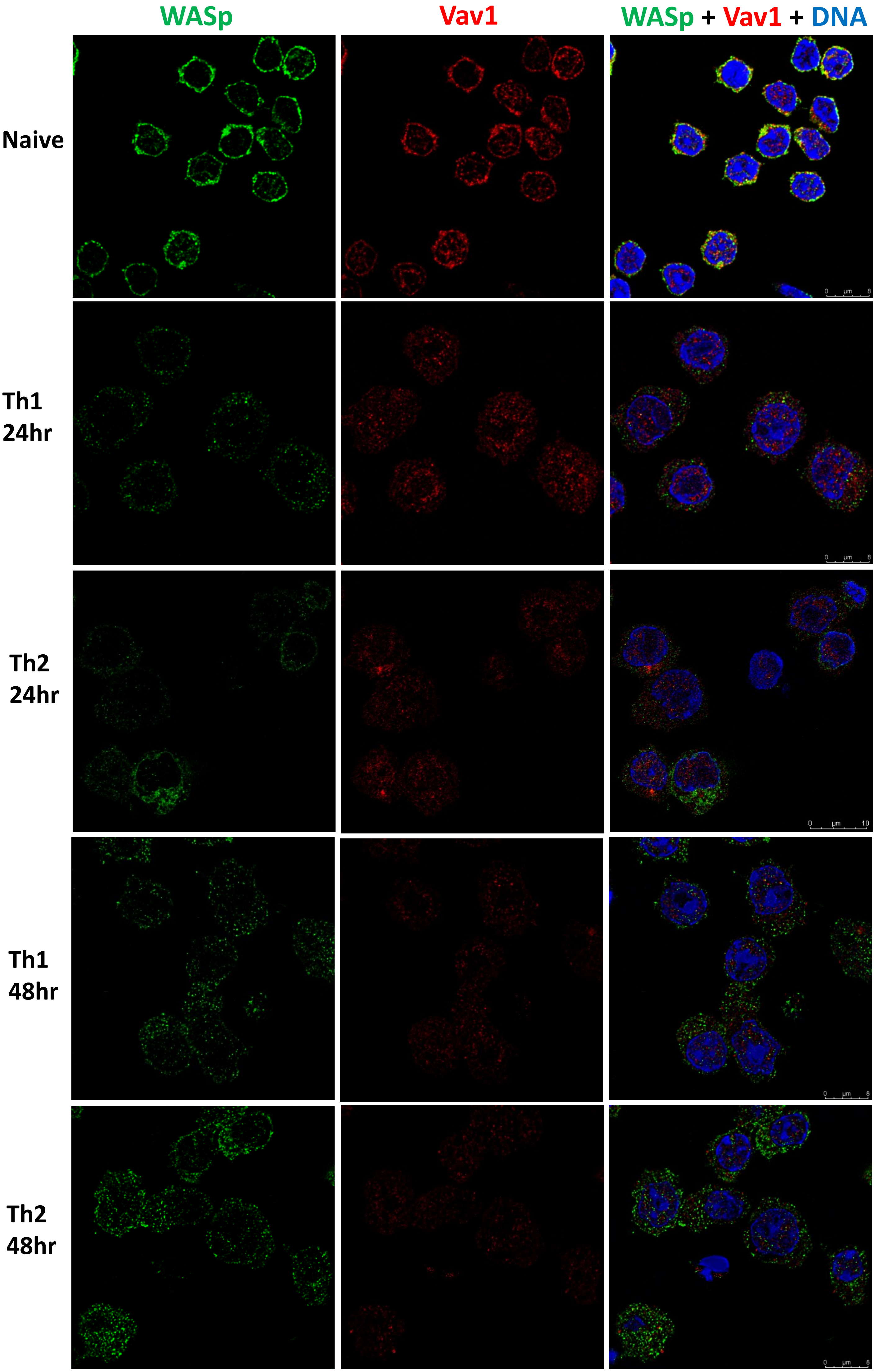
WASp
Vav1
WASp + Vav1 + DNA
Naive
Th1
24hr
Th2
24hr
Th1
48hr
Th2
48hr

q

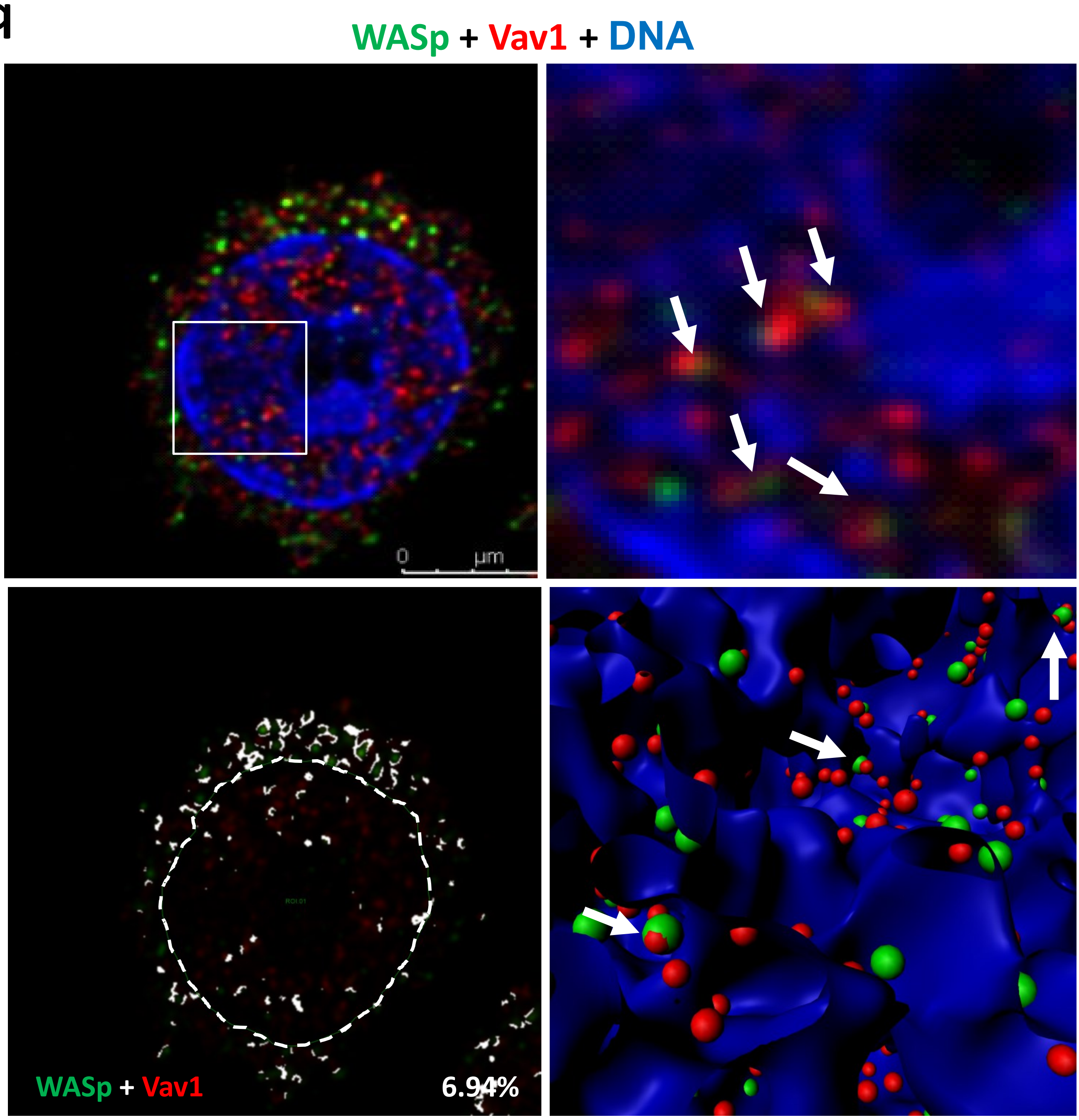

24hr-differentiating Th1 cells

r

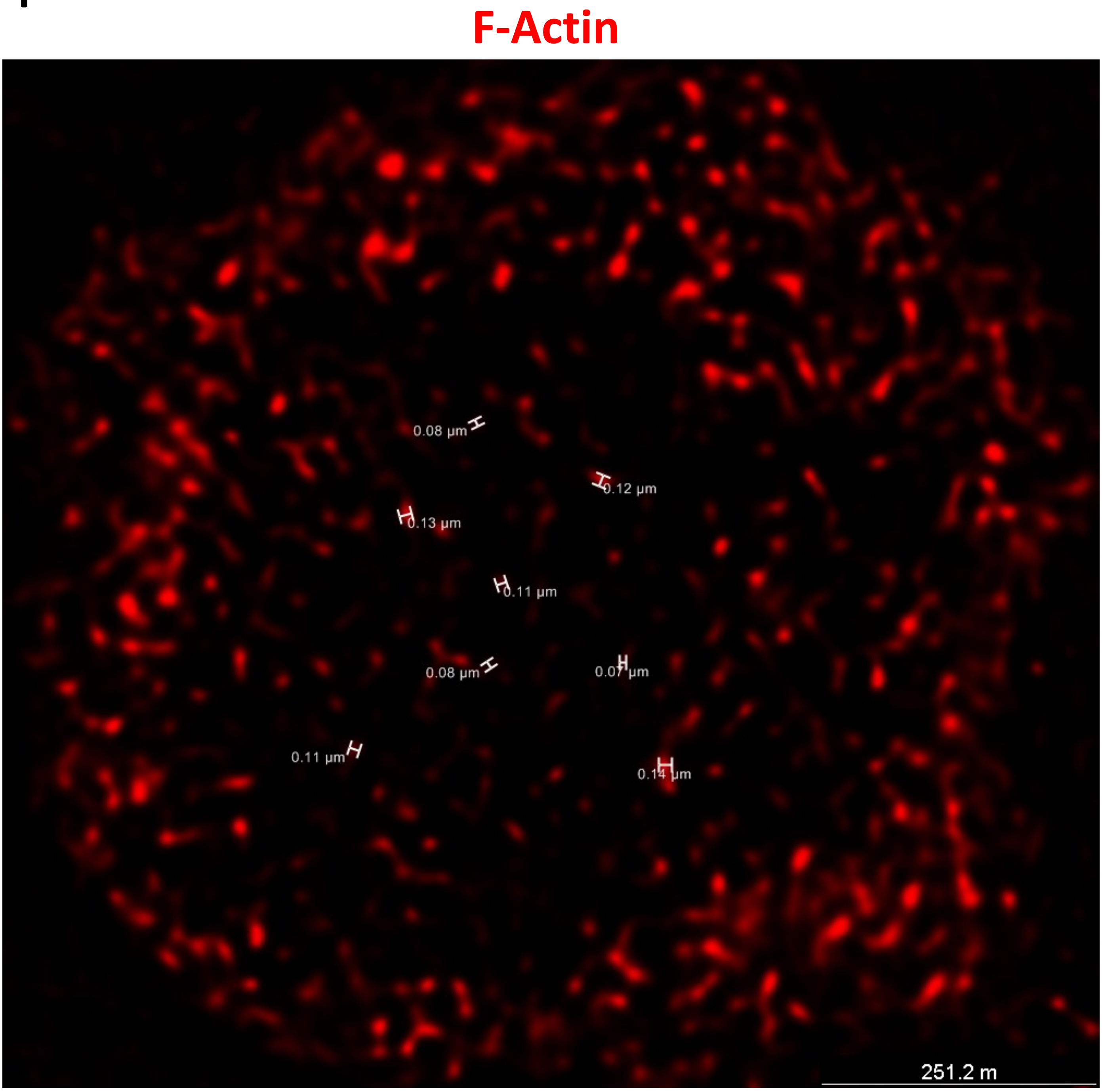

24hr- differentiating Th1 cells

Figure S1 (refers to Fig.1): Inducible nuclear F-actin in the 24hr-differentiating Th cells. (a) Immunofluorescence staining of naïve, 24hr- and 48hr-differentiating Th1 and Th2 cells with DAPI (blue) and Phalloidin (red). **(b)** Z stacks of representative images of naïve and Th2 cells from **a** were combined into computational 3D-structure using the IMARIS 9.5 software. The experiments were performed in 4 independent biological replicates with similar results. **(c)** Immunofluorescence staining of 24hr-differentiating Th1 cells using Phalloidin (red) and anti-CD3ε Ab. **(d)** Z stacks of representative images from **c** were combined into 3D-structure. **(e)** Immunofluorescence image of the 24hr-differentiating Th1 using the Chamber Slide System protocol, staining with Phalloidin (red), anti-CD3ε Ab (green), which could barely access the surface-attached CD3ε, and stained only some exposed rims, and Hoechst (blue). **(f)** Immunofluorescence image of the 24hr-differentiating Th1 using the cytospin protocol, staining with Phalloidin (red) and Hoechst (blue). **(g) (up)** Immunofluorescence image of 24hr-differentiating Th1 cells staining with SirActin (red) for F-actin visualization **(lower, left)** followed by zoom-in using STED microscope, and **(lower, right)** computational 3D-image (IMARIS). The experiments were performed in two independent biological replicates with similar results. **(h-k)** Immunofluorescence image of naïve, 24hr- and 48hr-differentiating Th1 and Th2 cells using Phalloidin (red), and anti-Vav1 Ab (green). **(i)** Zoom-in of naïve and **(j)** 48hr-differentiated Th1 cells followed by magnification of the white square. (**bottom left**) Colocalization rate of nuclear Vav1 and F-actin (overlapping signal in white). The white dashed line, which was determined by Hoechst staining, defines the nuclear periphery (ROI) for nuclear colocalization assessment. (**bottom right**) Colocalization rate of nuclear Vav1 and chromatin. **(k)** Computational 3D-structure of the indicated images. **(m-o)** Immunofluorescence staining of naïve, 24hr- and 48hr-differentiating Th1 and Th2 cells. Staining was performed using Phalloidin (red), in parallel with anti-WASp Ab (green). **(n)** As in **m**, staining the DNA and secondary Ab only. **(o)** zoom-in of immunofluorescence image of the 24hr-differentiating Th1 cells followed by magnification of the white square. (**middle left**) Colocalization rate of nuclear WASp and chromatin. (**middle right**) Colocalization rate of nuclear WASp and F-actin. (**bottom**) 3D-structure of the indicated image. **(p,q)** Immunofluorescence imaging of naïve, 24hr- and 48hr-differentiating Th1 and Th2 cells. Staining was performed using anti-WASp Ab (green) in parallel with anti-Vav1 Ab (red). **(q)** Immunofluorescence image of 24hr-differentiating Th1 cells followed by magnification of the white square. (**bottom left**) Colocalization rate of nuclear Vav1 and WASp. (**bottom right**) 3D-structure of the indicated image. The experiments were performed in 4 independent biological replicates with similar results. **(r)** F-actin width (μm) measurement of the filaments in the 24hr-differentiated Th1 cell from **a** using LasX analysis software. Secondary Ab only was used as Control (a, n). The experiments were performed in four independent biological replicates with similar results. Images were acquired by SR Hyvolution microscope or STED microscope, as indicated.

Figure S2

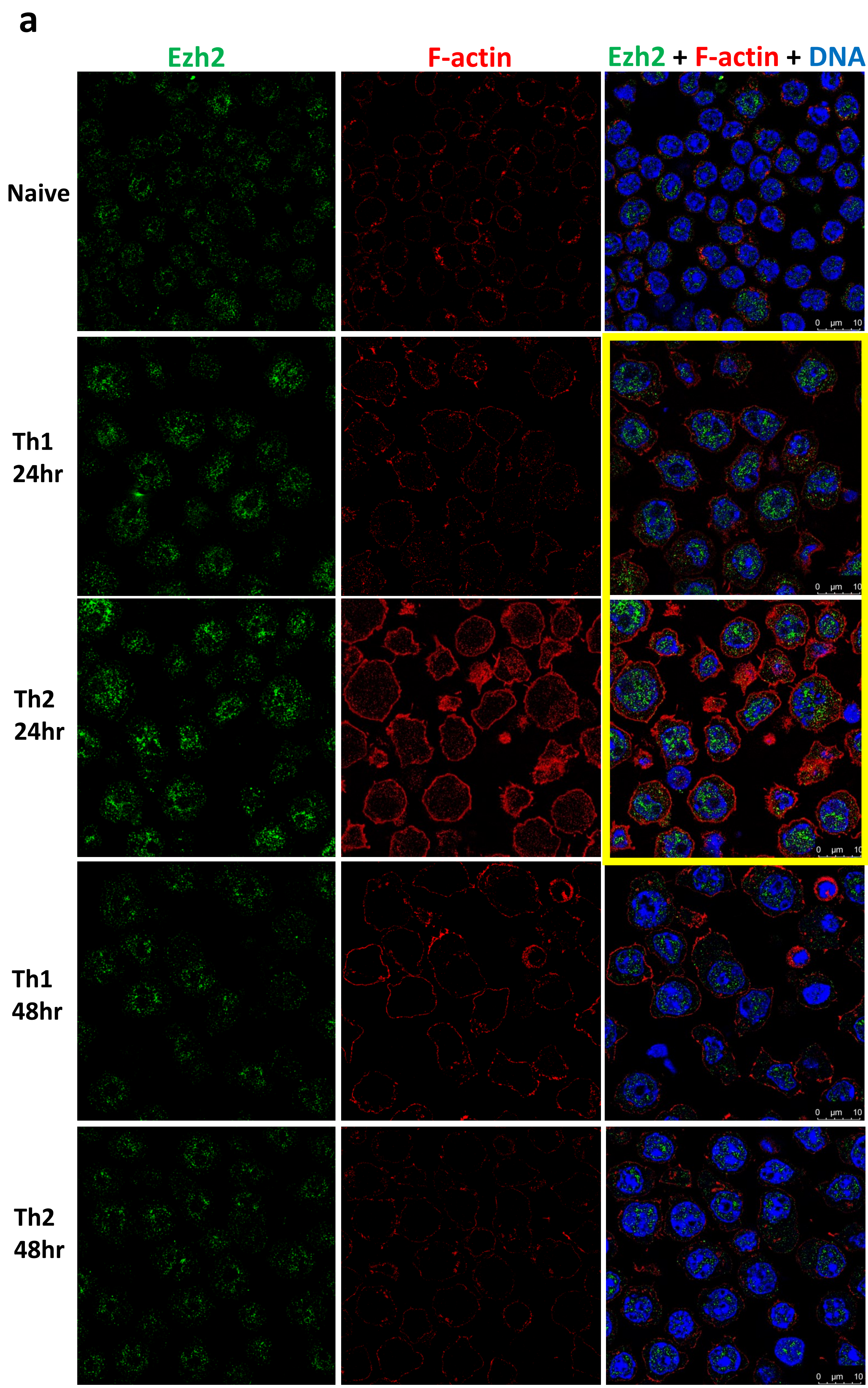

The yellow box is enlarged in the next page

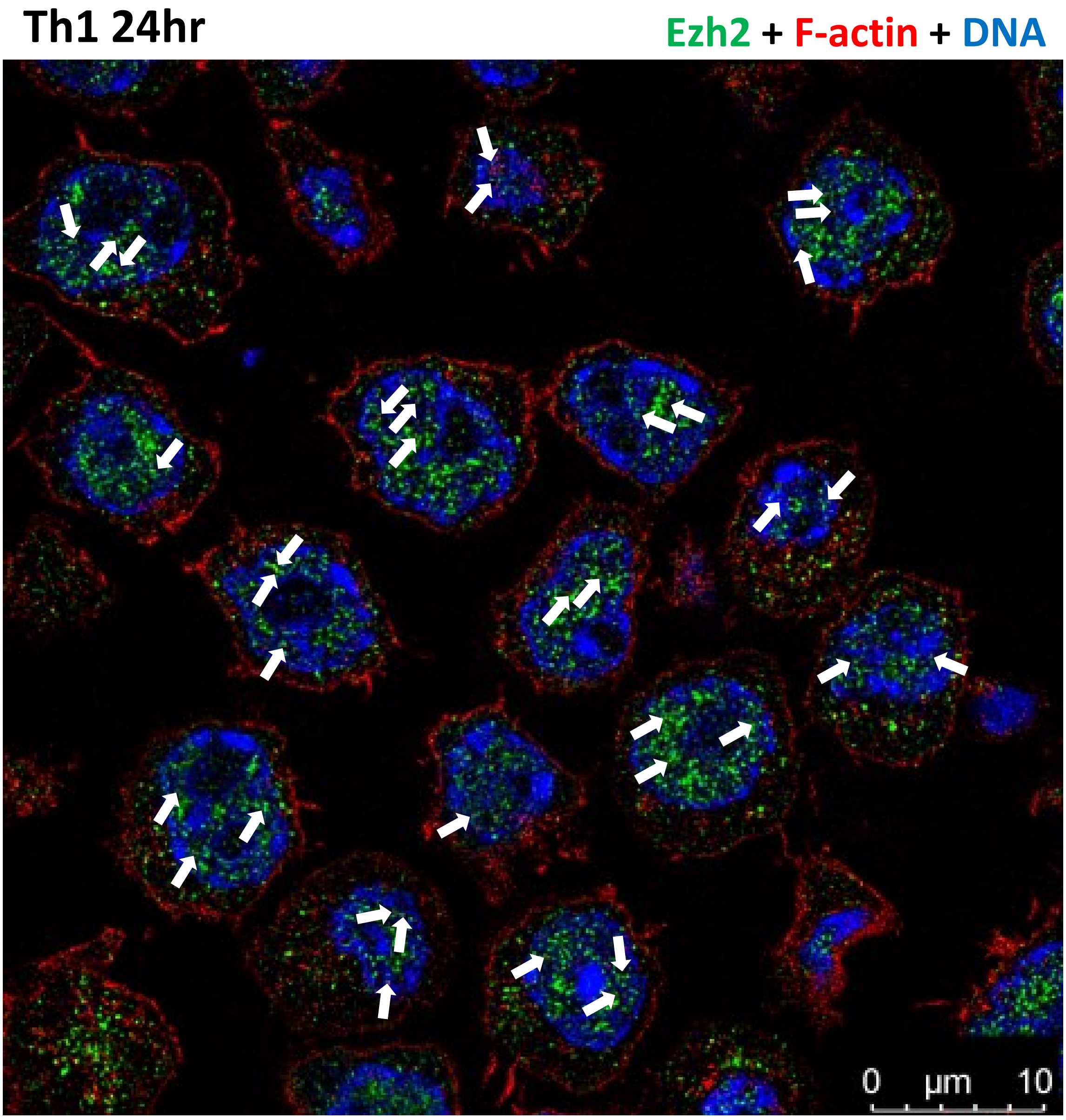
Th1 24hr
Ezh2 + F-actin + DNA
0 μm 10

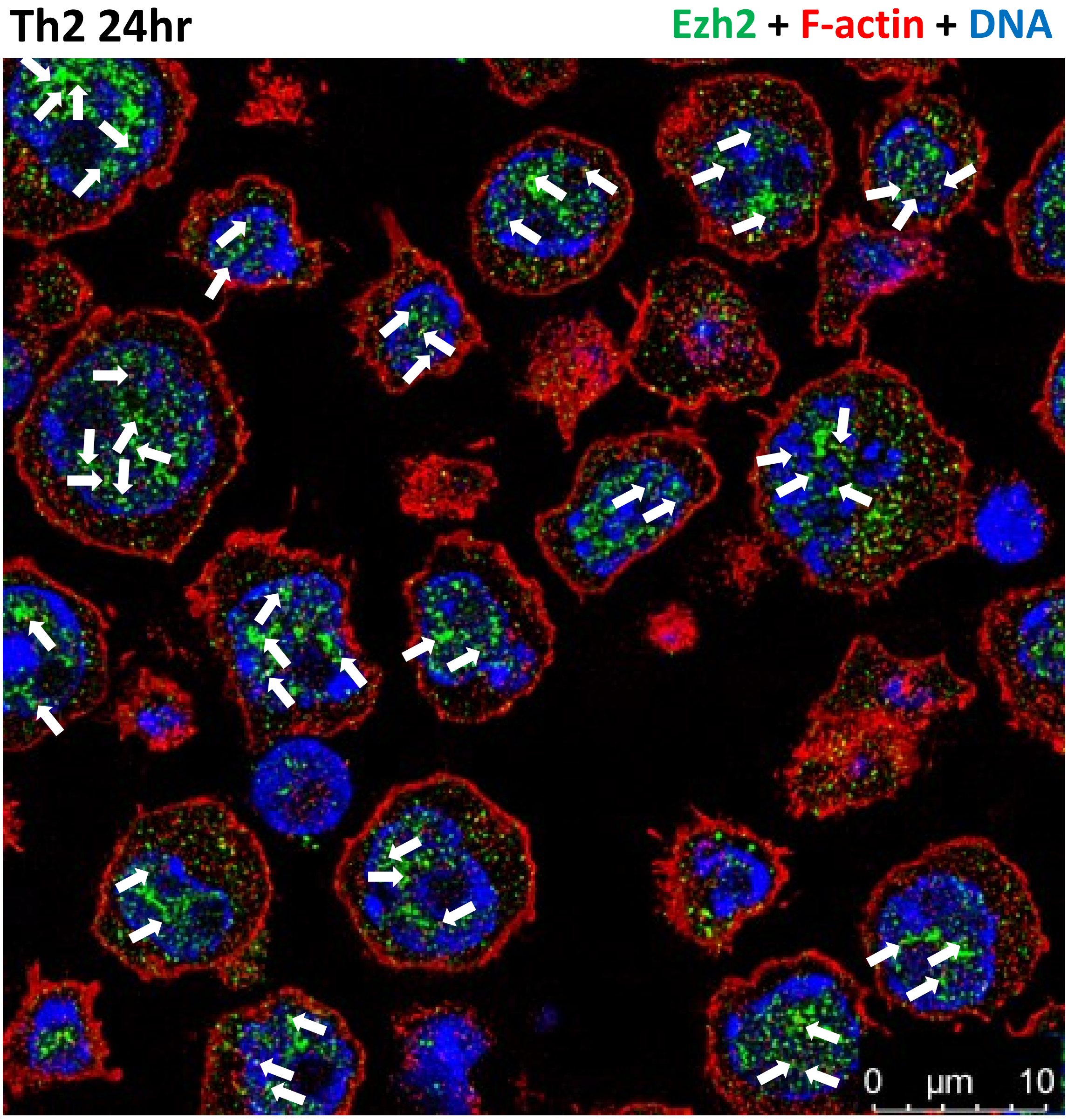
Th2 24hr
Ezh2 + F-actin + DNA
0 μm 10

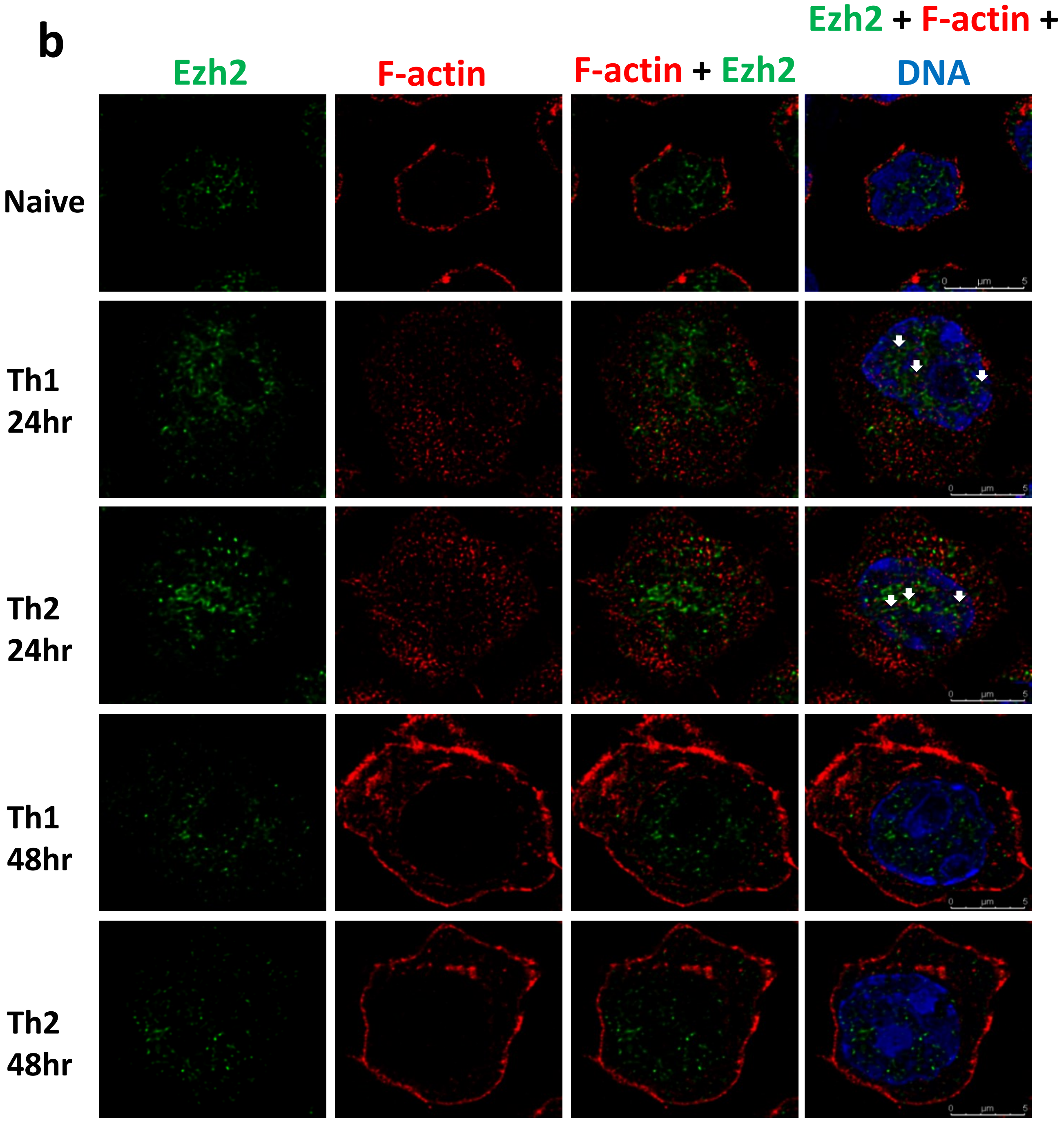
b
Ezh2
F-actin
F-actin + Ezh2
Ezh2 + F-actin + DNA
Naive
Th1 24hr
Th2 24hr
Th1 48hr
Th2 48hr

c

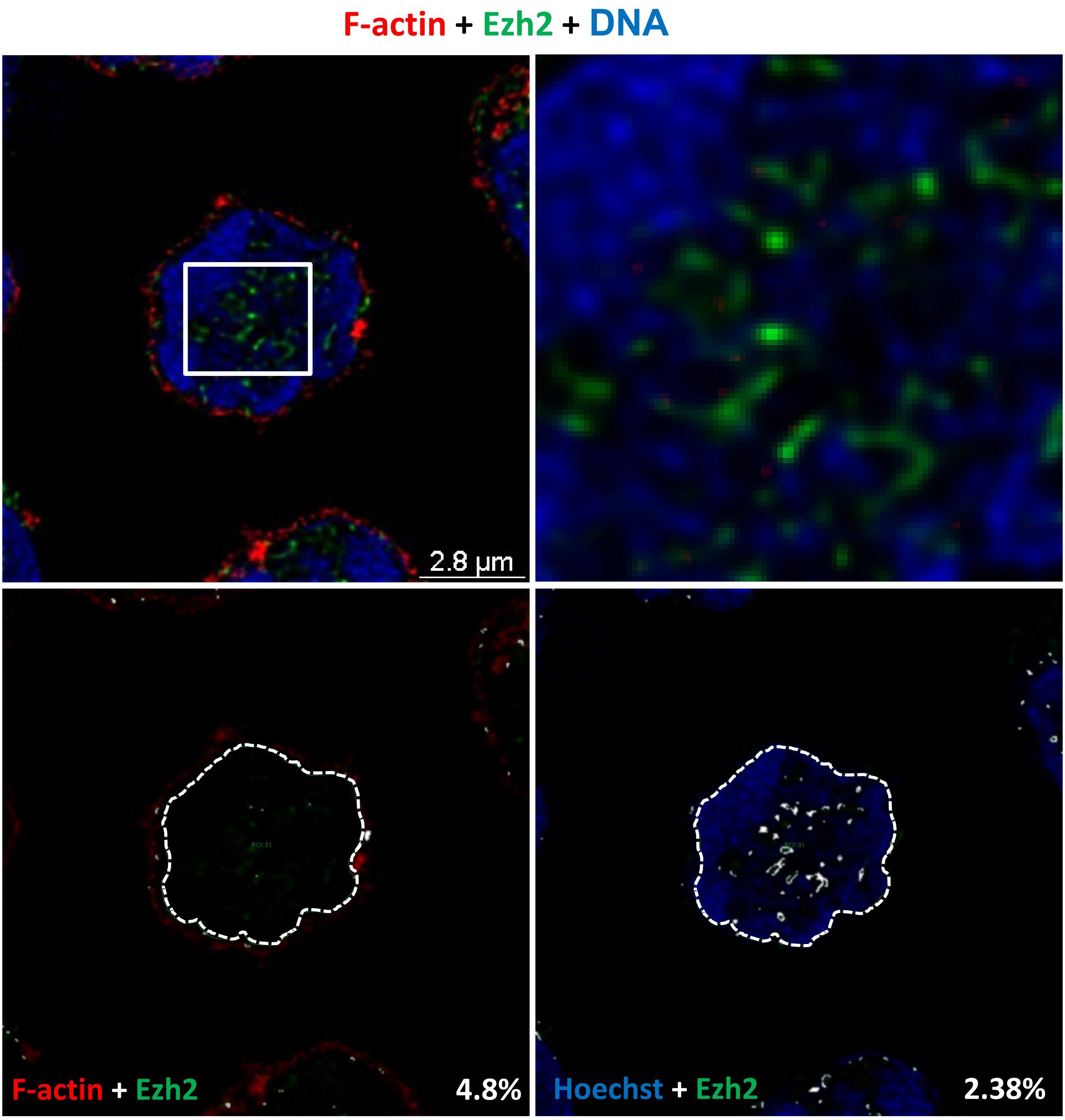

Naïve Th cells

d

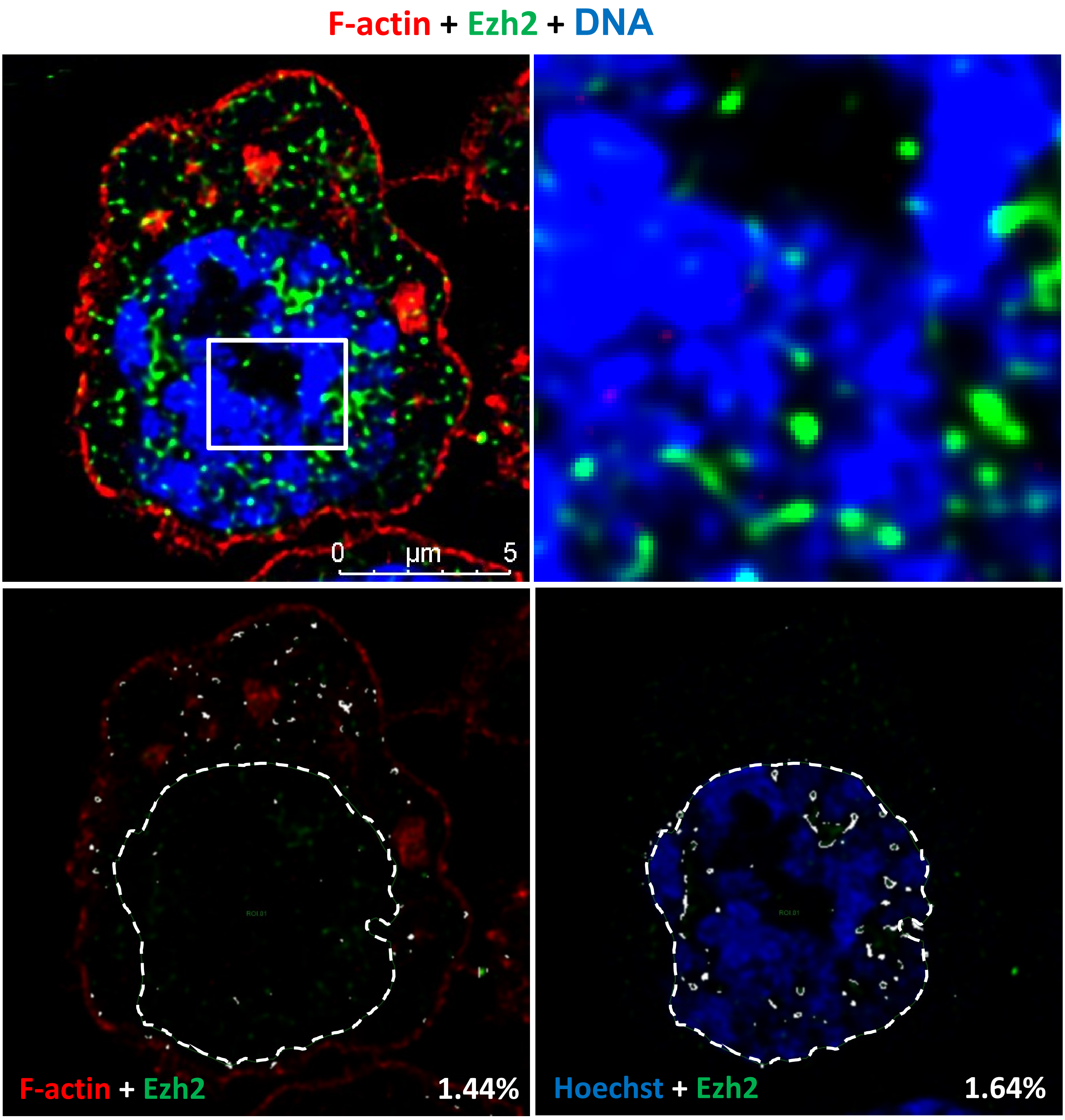

48hr- differentiating Th1 cells

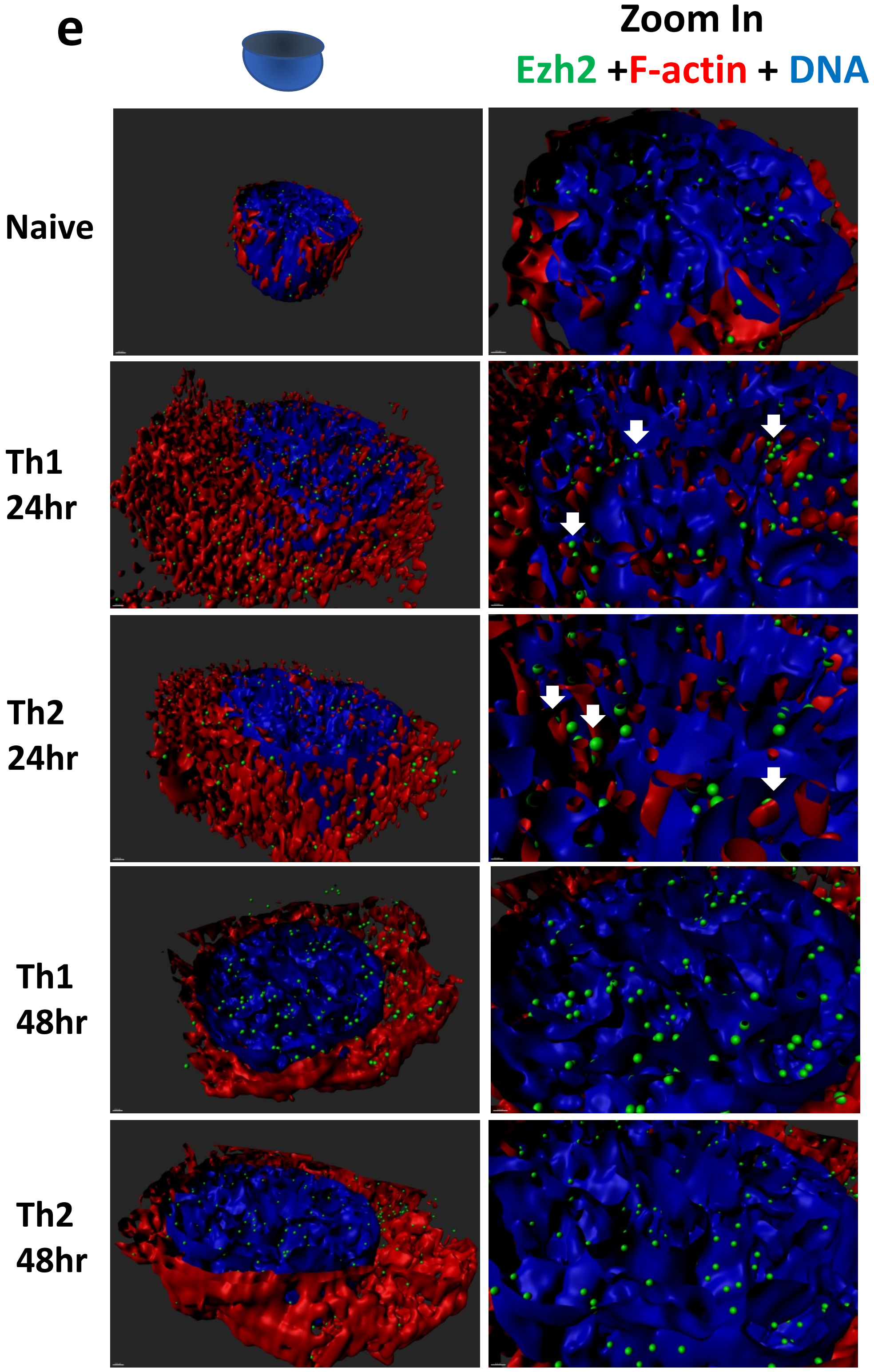
e
Zoom In
Ezh2 +F-actin + DNA
Naive
Th1
24hr
Th2
24hr
Th1
48hr
Th2
48hr

Figure S2 (refers to Fig.2): **Ezh2 is associated with the nuclear F-actin and the actin machinery**. **(a)** Immunofluorescence image of naïve, 24hr- and 48hr-differentiating Th1 and Th2 cells. Staining was performed using Phalloidin (red), simultaneously with anti-Ezh2 Ab (green). DNA was stained with Hoechst (blue). **(b)** Magnification of selected cells from **a**. **(c, d) (upper left)** Zoom-in of the images of naïve (c) and 48hr-differentiating Th1 cells (d) followed by magnification of the white square in upper right. **(lower left)** Colocalization rate of nuclear Ezh2 and F-actin. **(lower right)** Colocalization rate of nuclear Ezh2 and chromatin. **(e)** 3D-structure of selected naïve, 24hr- and 48hr-differentiating Th1 and Th2 cells from **b** using IMARIS 9.5 software; Ezh2 (green) and F-actin (red). The experiments were performed in three independent biological replicates with similar results.

Figure S3

a

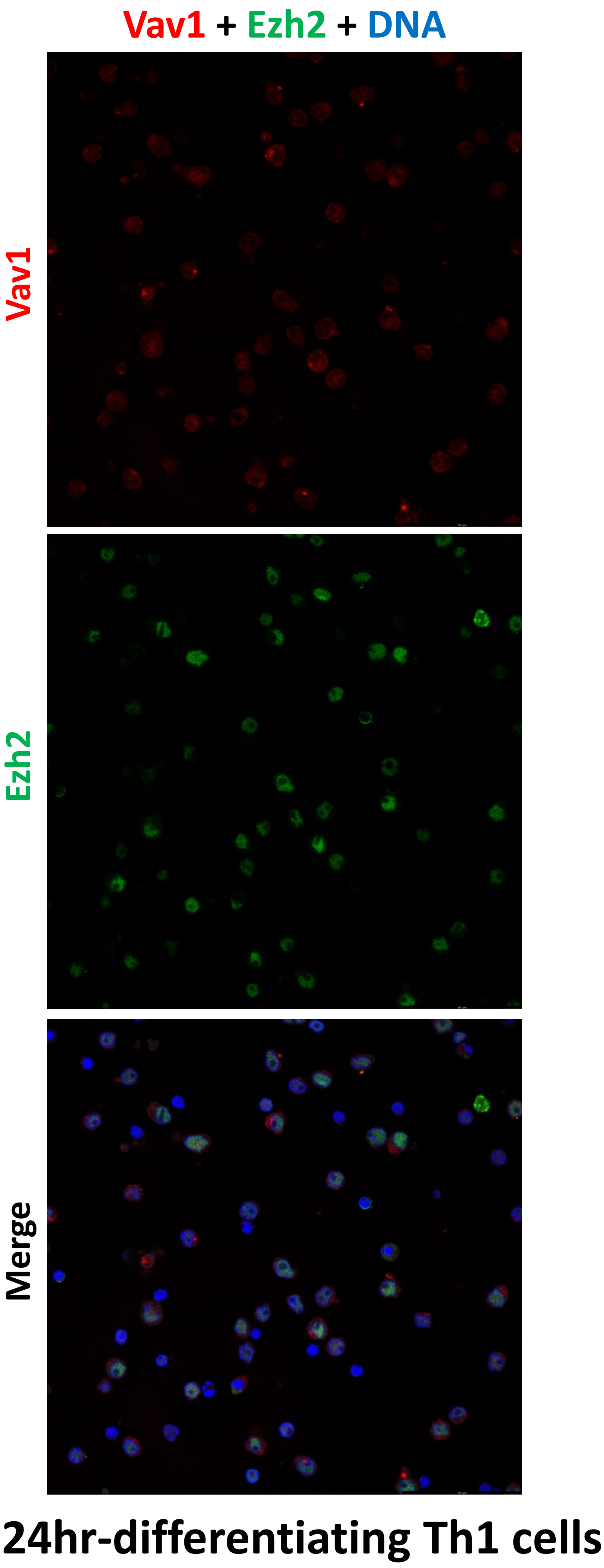
Vav1 + Ezh2 + DNA
Vav1
Ezh2
Merge
24hr-differentiating Th1 cells

b

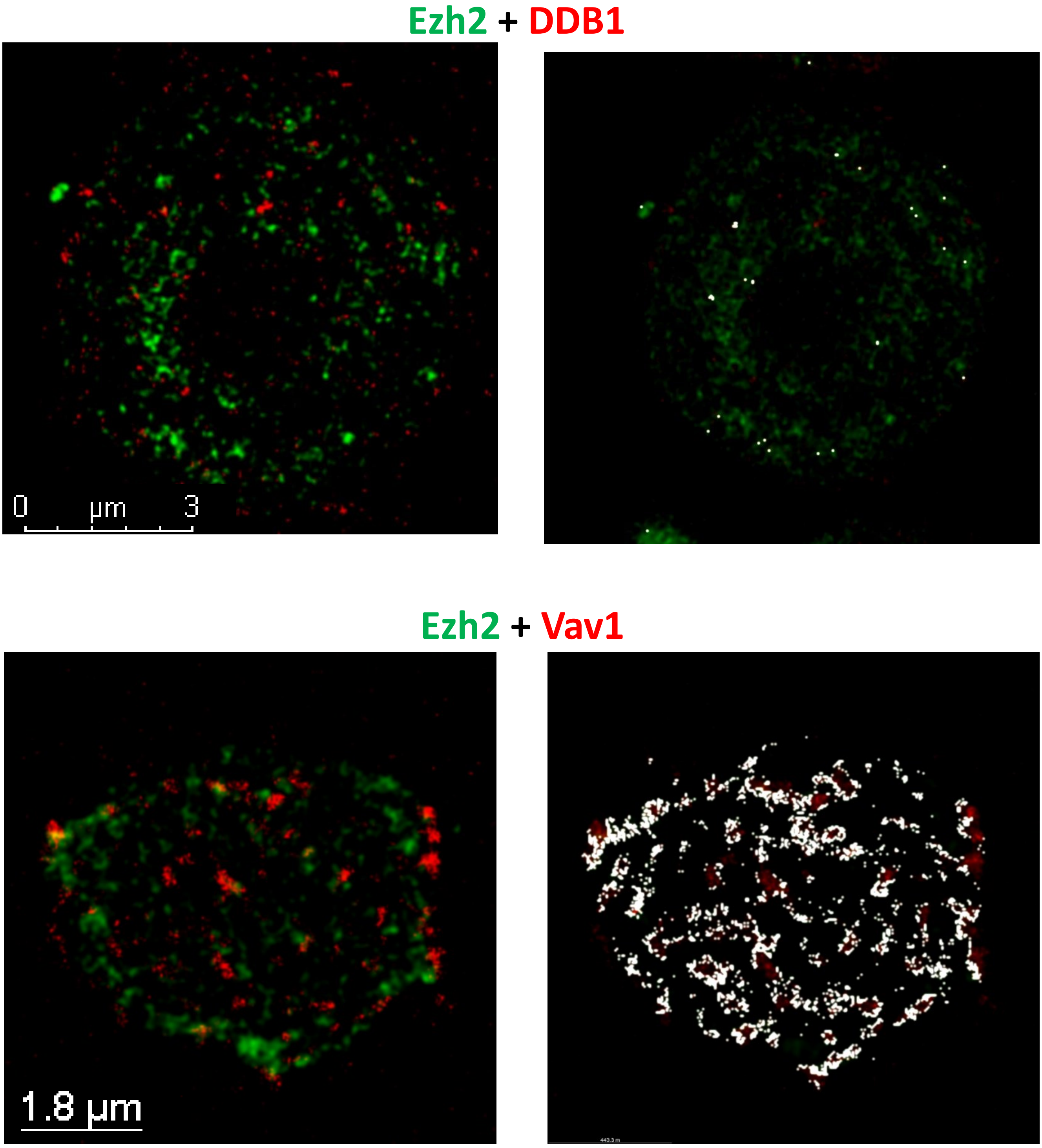

24hr-differentiating Th1 cells

Whole cell colocalization

c

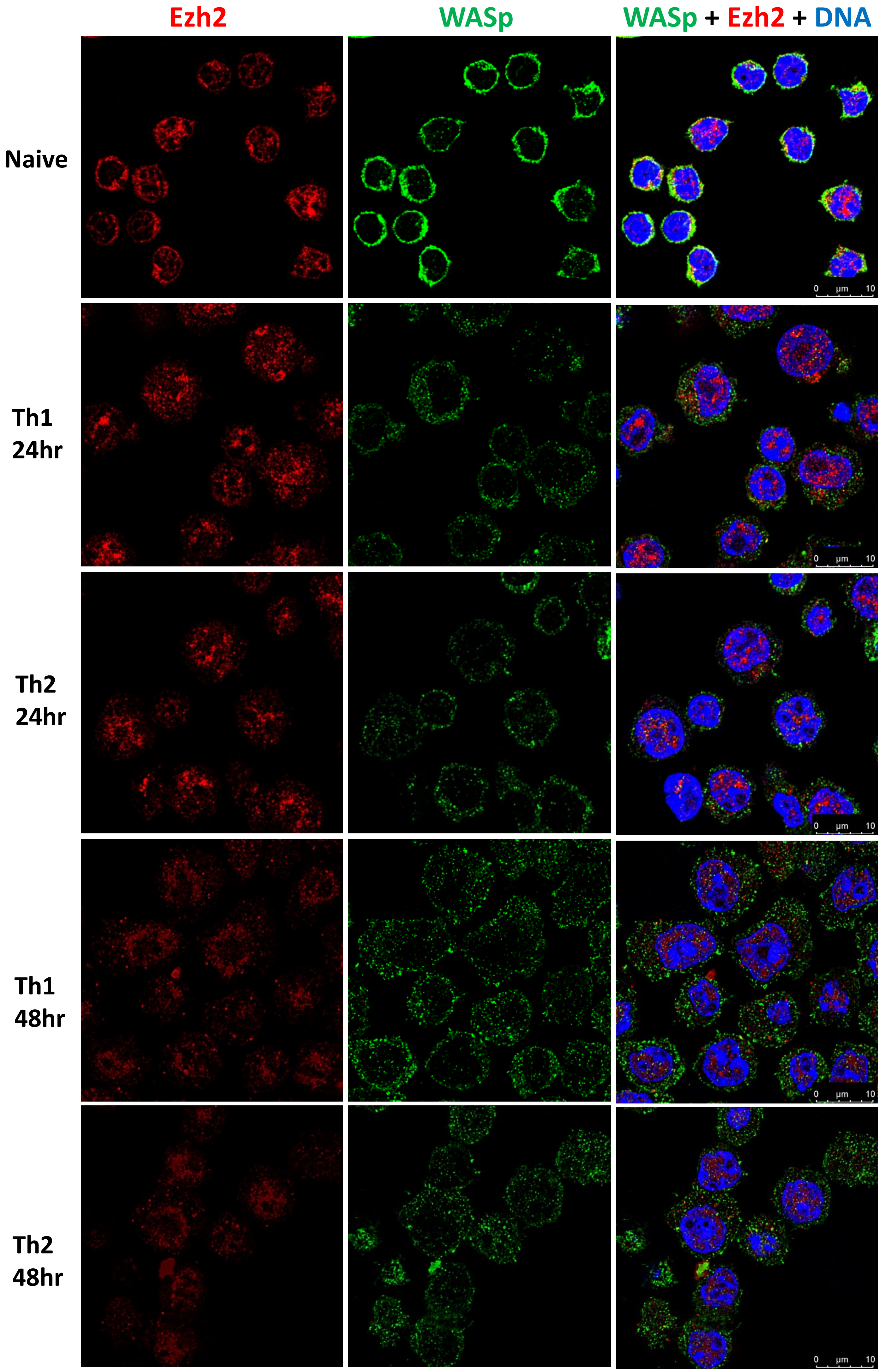
Ezh2
WASp
WASp + Ezh2 + DNA
Naive
Th1 24hr
Th2 24hr
Th1 48hr
Th2 48hr
0 µm 10

d

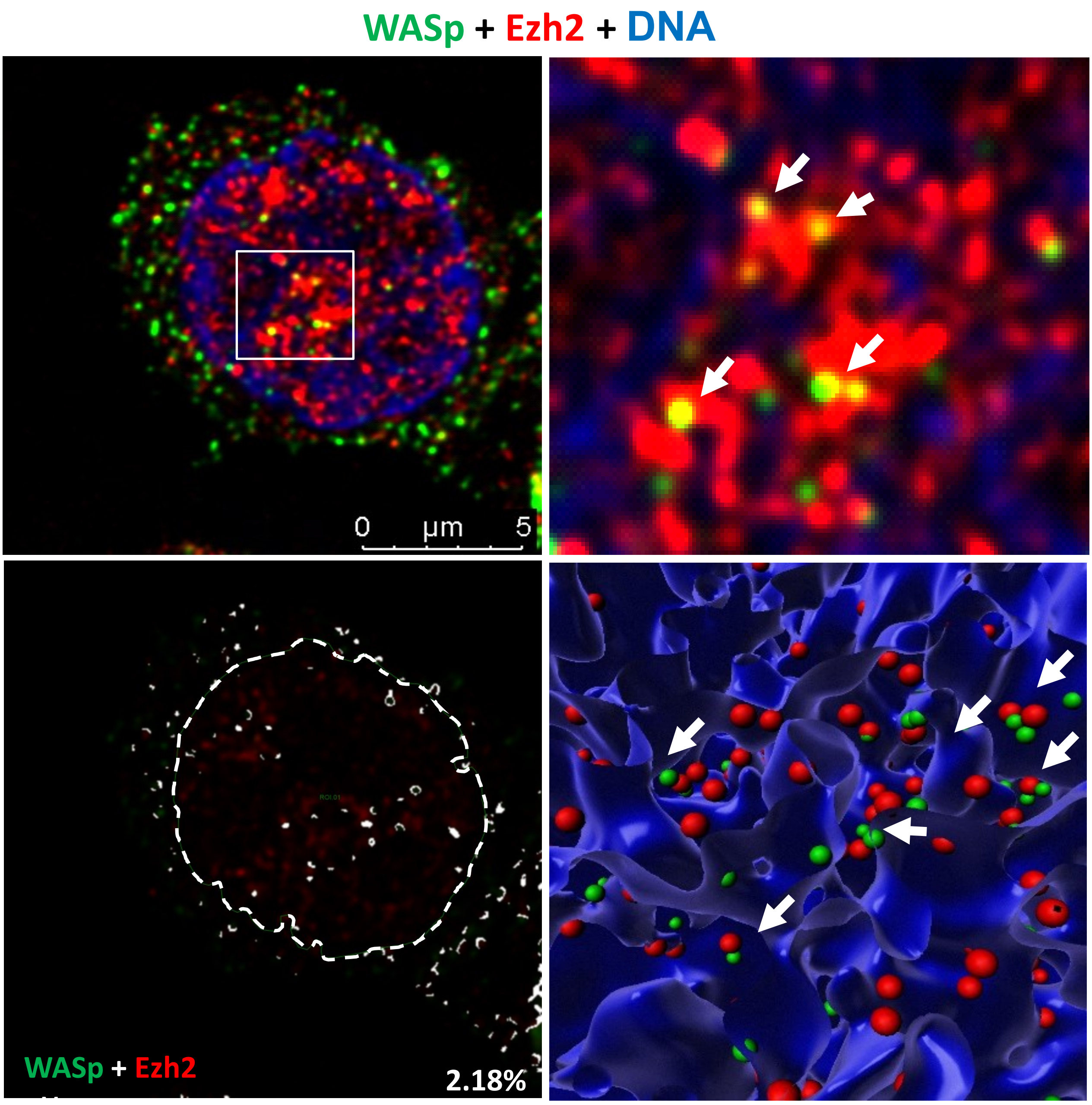

24hr-differentiating Th1 cells

e

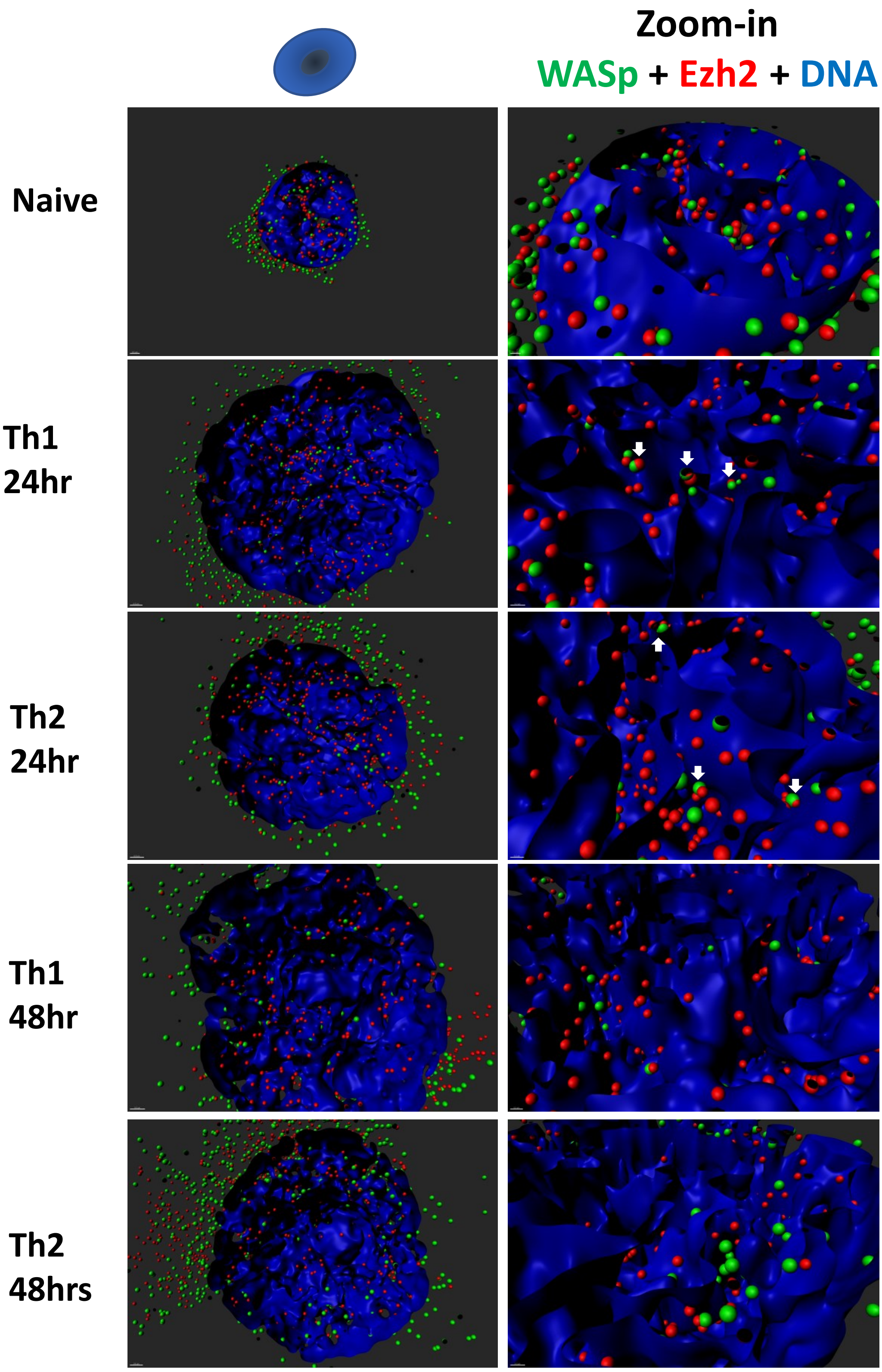
Zoom-in
WASp + Ezh2 + DNA
Naive
Th1
24hr
Th2
24hr
Th1
48hr
Th2
48hrs

f

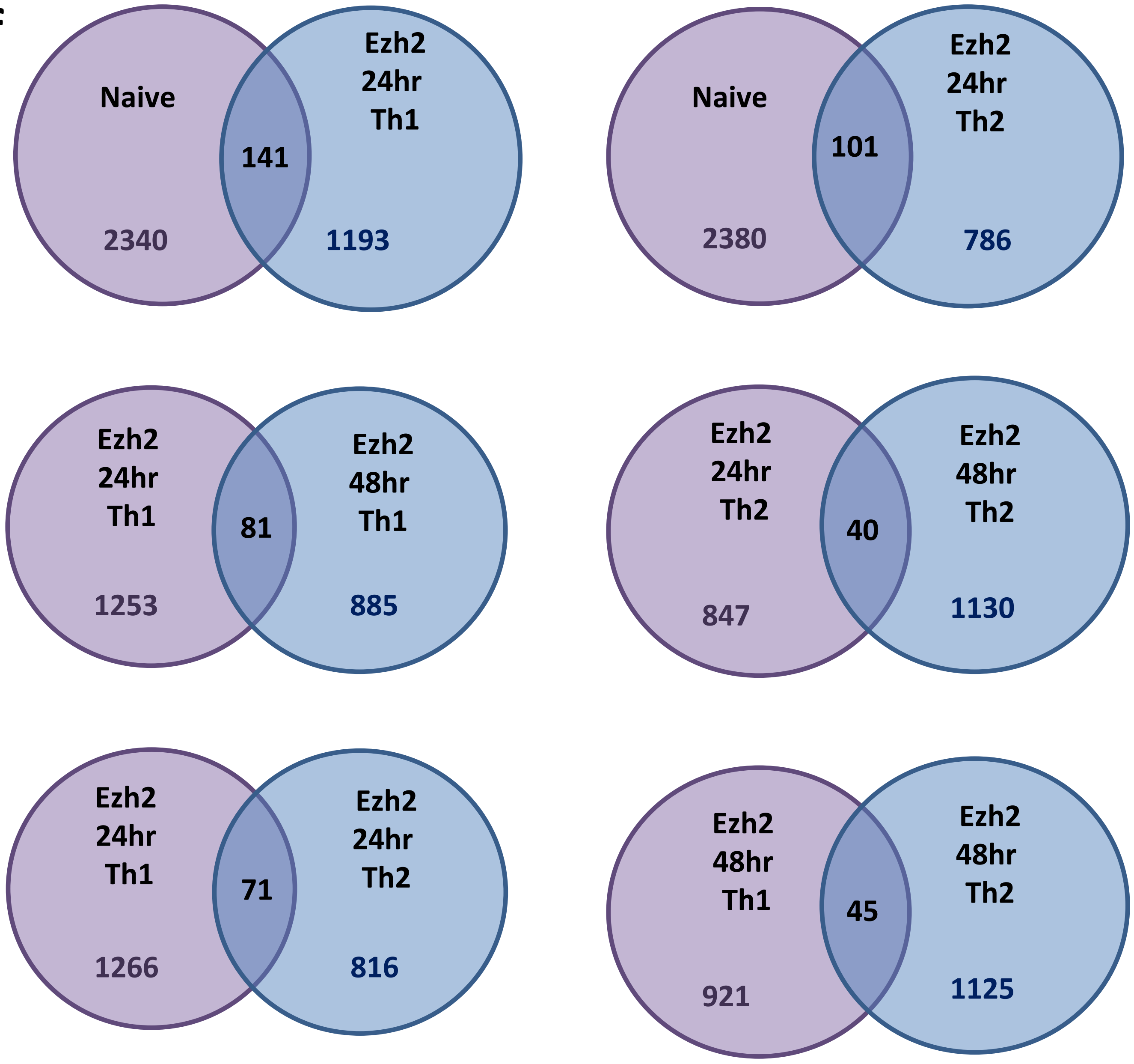

g **24hr differentiating Th1 cells**
Profiling of Ezh2/Vav1 binding sites

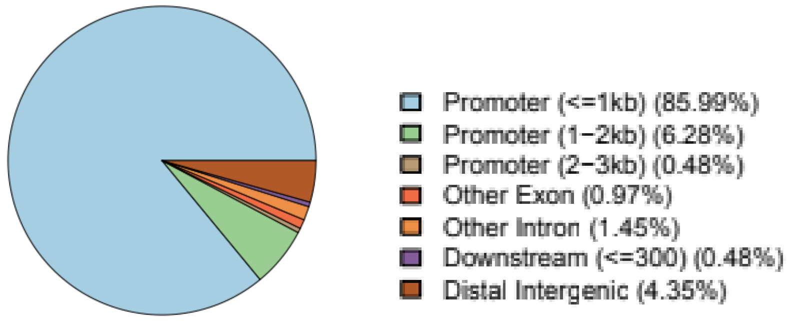

24hr differentiating Th2 cells
Profiling of Ezh2/Vav1 binding sites

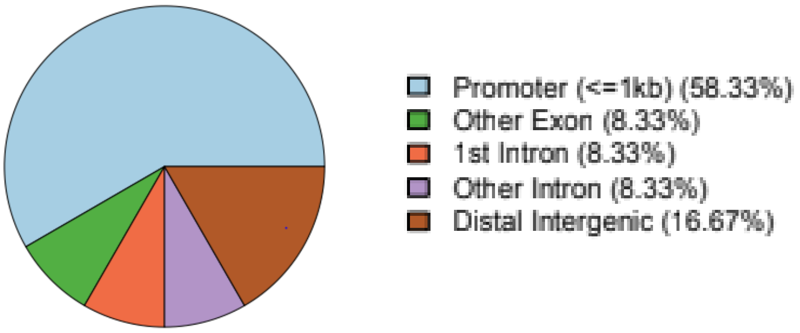

h

24hr differentiating Th1 cells
Profiling of Ezh2/Vav1 binding sites

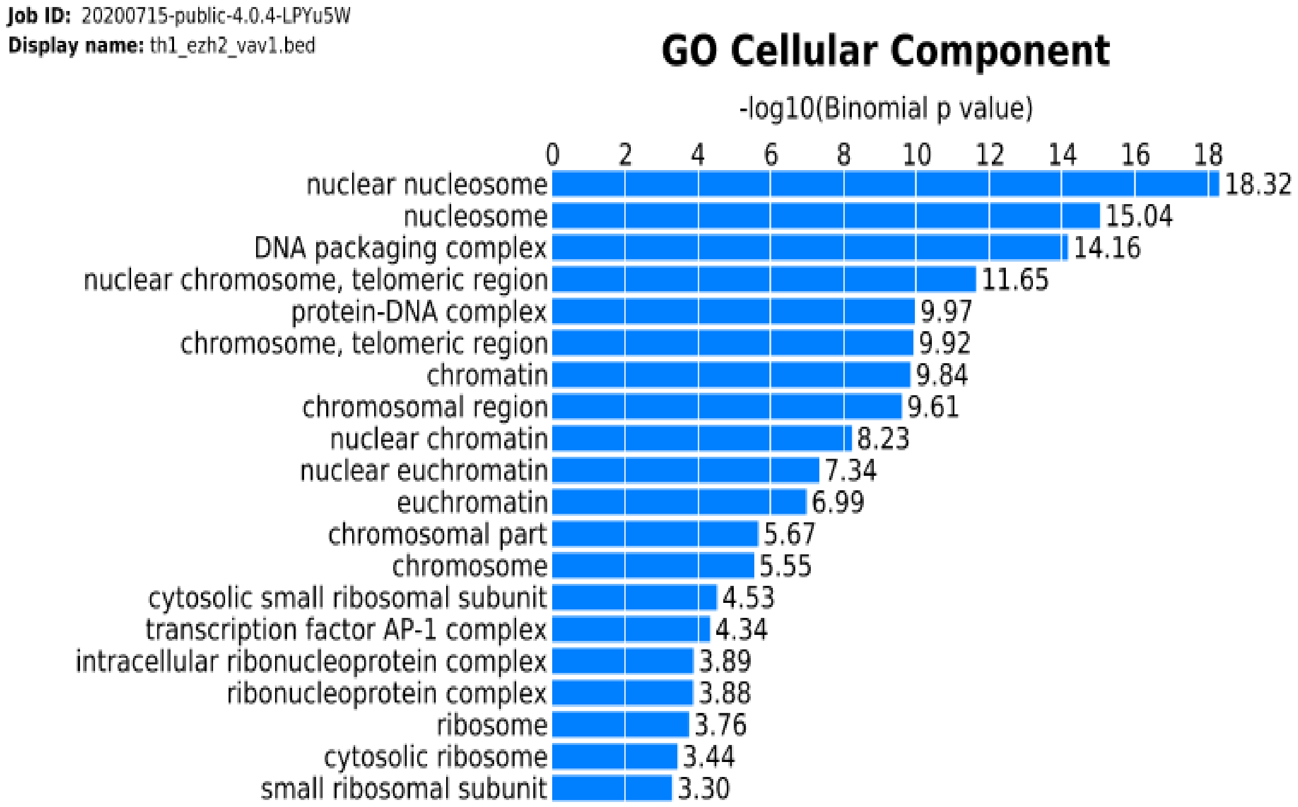

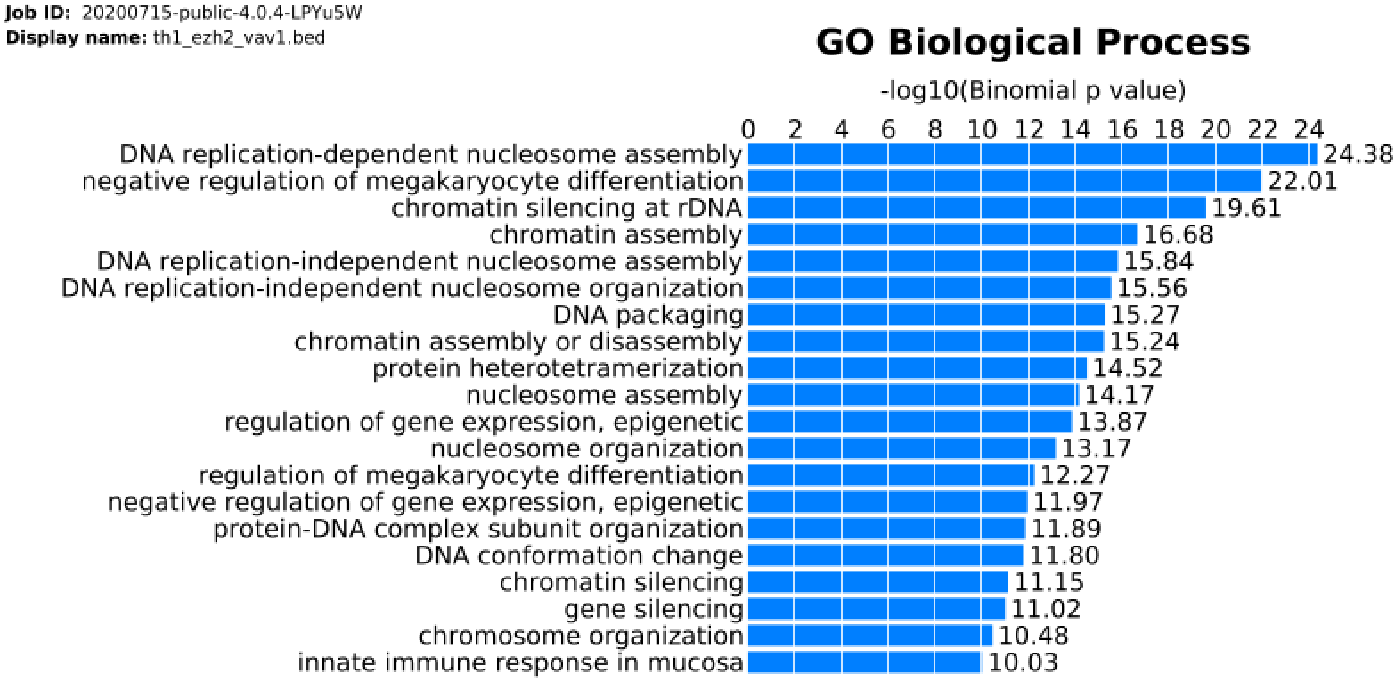

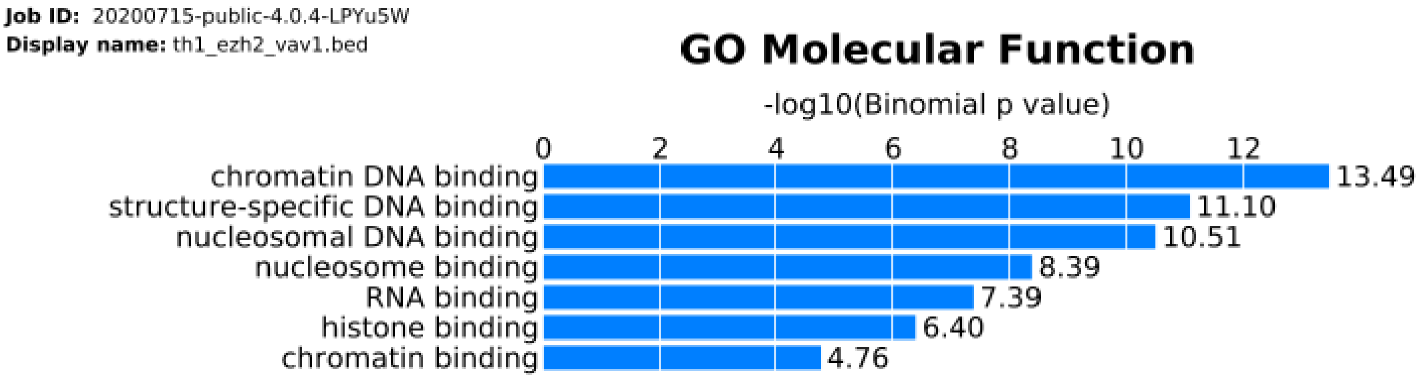

i

j

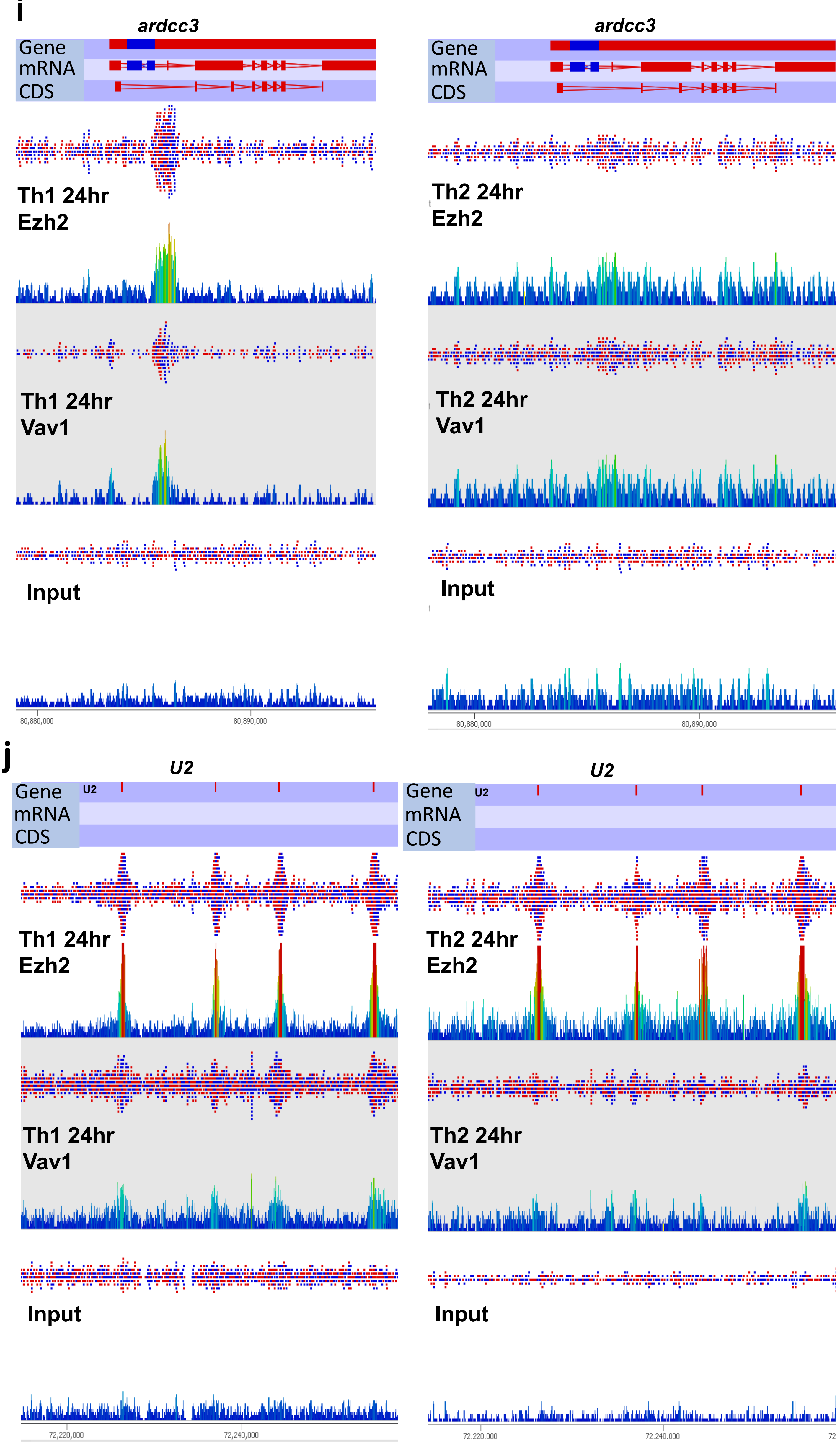

ardcc3
Gene
mRNA
CDS
Th1 24hr
Ezh2
Th1 24hr
Vav1
Input
80,880,000
80,890,000
ardcc3
Gene
mRNA
CDS
Th2 24hr
Ezh2
Th2 24hr
Vav1
Input
80,880,000
80,890,000
U2
Gene
mRNA
CDS
U2
Th1 24hr
Ezh2
Th1 24hr
Vav1
Input
72,220,000
72,240,000
U2
Gene
mRNA
CDS
U2
Th2 24hr
Ezh2
Th2 24hr
Vav1
Input
72,220,000
72,240,000

Figure S3 (refers to Fig.3): Nuclear Ezh2 is colocalized with F-actin, WASp and chromatin-associated Vav1. (a) Immunofluorescence image of 24hr-differentiating Th1 cells, staining with anti-Ezh2 Ab (green) in parallel with anti-Vav1 Ab (red). DNA was stained with Hoechst (blue). **(b) (upper left)** Immunofluorescence staining of the 24hr-differentiating Th1 cells using anti-Ezh2 Ab (green) and anti-DDB1 Ab (red), and the colocalization of nuclear Ezh2 and DDB1. **(lower left)** Immunofluorescence staining of the 24hr-differentiating Th1 cells using anti-Ezh2 mouse monoclonal Ab (green) and anti-Vav1 Ab (red), and the colocalization of nuclear Ezh2 and Vav1. The experiments were performed in two independent biological replicates with similar results. Images were acquired by SR STED microscope. **(c-e)** Immunofluorescence imaging of naïve, 24hr- and 48hr-differentiating Th1 and Th2 cells, staining with anti-Ezh2 Ab (red), in parallel with anti-WASp Ab (green) and Hoechst (blue). **(d)** Immunofluorescence image of zoomed-in 24hr-differentiating Th1 cells followed by (**upper right**) magnification of the white square. (**bottom left**) Colocalization rate of nuclear WASp and F-actin. (**bottom right**) 3D-structure using the computational IMARIS 9.5 software. **(e)** 3D-structure of naïve, 24hr- and 48hr-differentiating Th1 and Th2 cells using IMARIS 9.5 software. Ezh2 (green), F-actin (red) and DNA (blue). The experiments were performed in three independent biological replicates with similar results. Images were acquired by SR Hyvolution microscope. **(f)** naïve and 24hr- and 48hr-differentiating Th1 and Th2 cells were subjected to ChIP-seq analysis using Ezh2 rabbit polyclonal antibodies (ab3748, Abcam). Binding peaks in naive, 24hr-and 48hr- differentiating Th1 and Th2 were identified using MACS and intersected by bedtools intersect interval in galaxy. **(g)** naïve and 24hr- and 48hr-differentiating Th1 and Th2 cells were subjected to ChIP-seq analysis using Ezh2 rabbit polyclonal Abs (ab3748, Abcam) and anti-Vav1 Abs (c14, Santa Cruz Biotechnology). Overlapping peaks of Ezh2 and Vav1 were analyzed by Chipseeker algorithm in galaxy environment for the distribution of the peaks near genomic features. **(h)** Ezh2/Vav1 common binding sites in 24hr-differentiating Th1 cells were analyzed by Great to predict biological processes of cis-regulatory elements. **(i,j)** Illustration of the binding activity of Ezh2 and Vav1 at the *ardcc3* and *U2* in Th cells. The peaks were plotted using wiggle plot in Seqmonk free software.

Figure S4

a

F-actin + Ezh2 + Vav1 + DNA

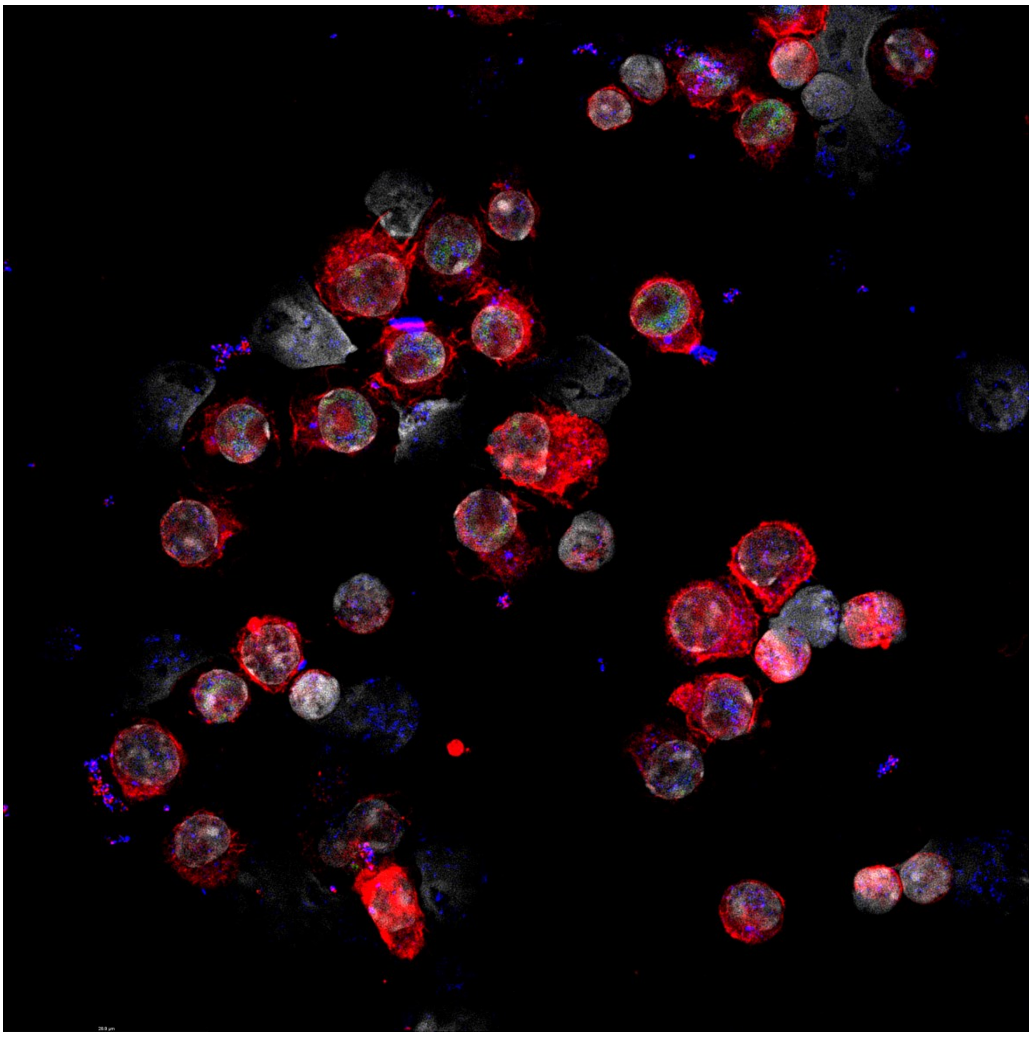

2nd Ab ONLY+ DNA

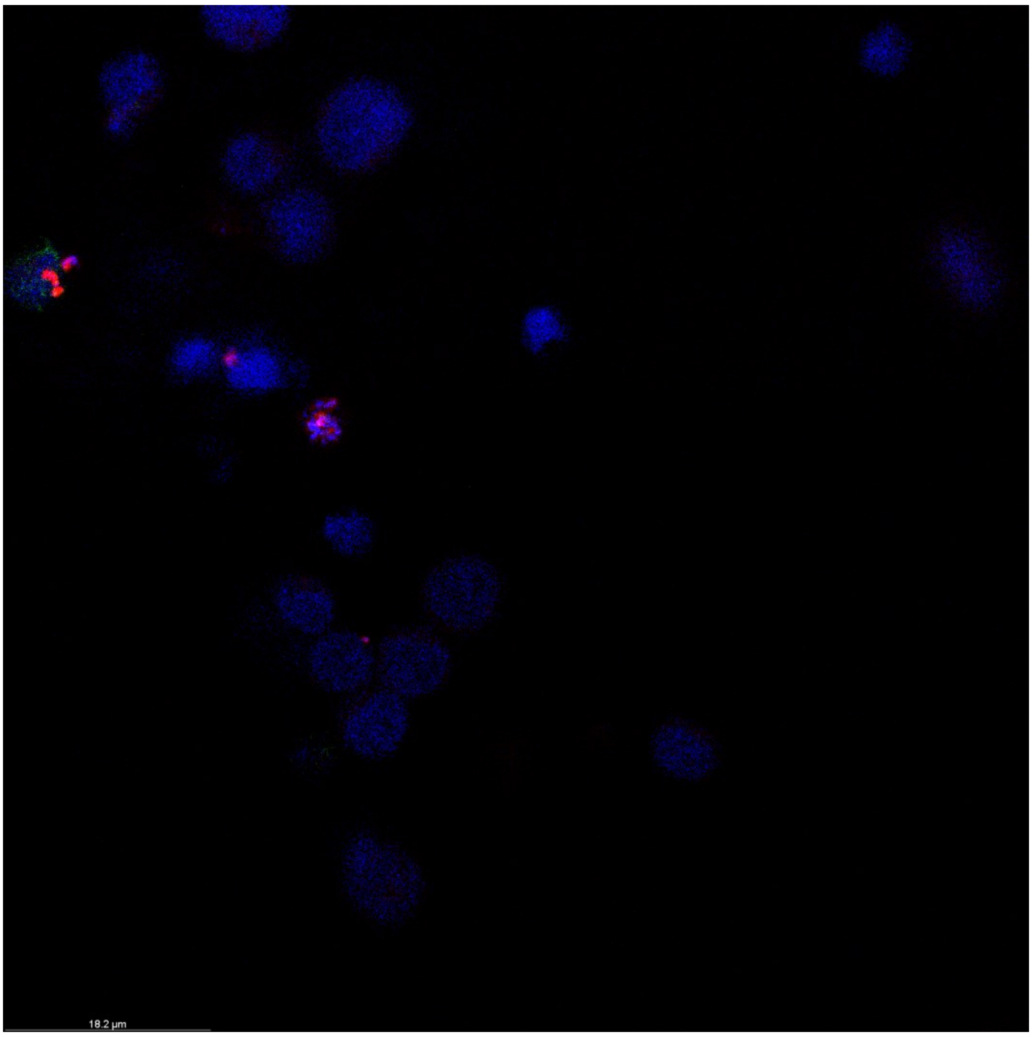

b

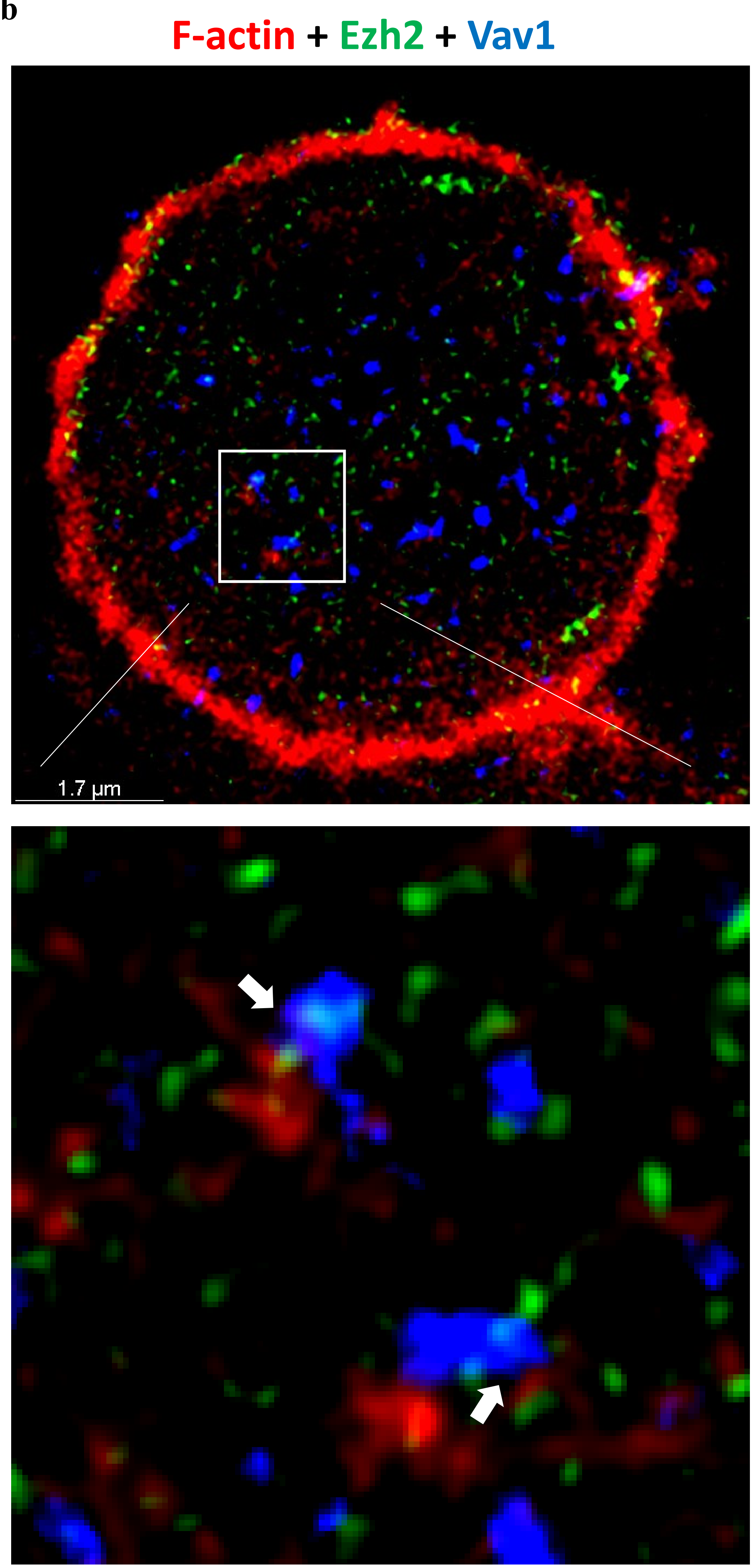

Figure S4 (refers to Fig. 4): Nuclear Ezh2/Vav1 are colocalized with F-actin at the chromatin context. (a) Immunofluorescence staining of 24hr-differentiating Th1 cells, using anti-Ezh2 mouse monoclonal Ab (green), in parallel with anti-Vav1 Ab (blue), Phalloidin (red) and Hoechst (grey). Secondary Ab only was used as Control. **(b) (upper)** SR STED Immunofluorescence staining of 24hr-differentiating Th1 cells using anti-Ezh2 mouse monoclonal Abs (green), in parallel with anti-Vav1 Ab (H211, Santa Cruz Biotechnology; blue) and Phalloidin (red). **(bottom)** magnification of the white square. The experiments were performed in two independent biological replicates with similar results. Images were acquired by either SR Hyvolution or STED microscope, as indicated.

Figure S5

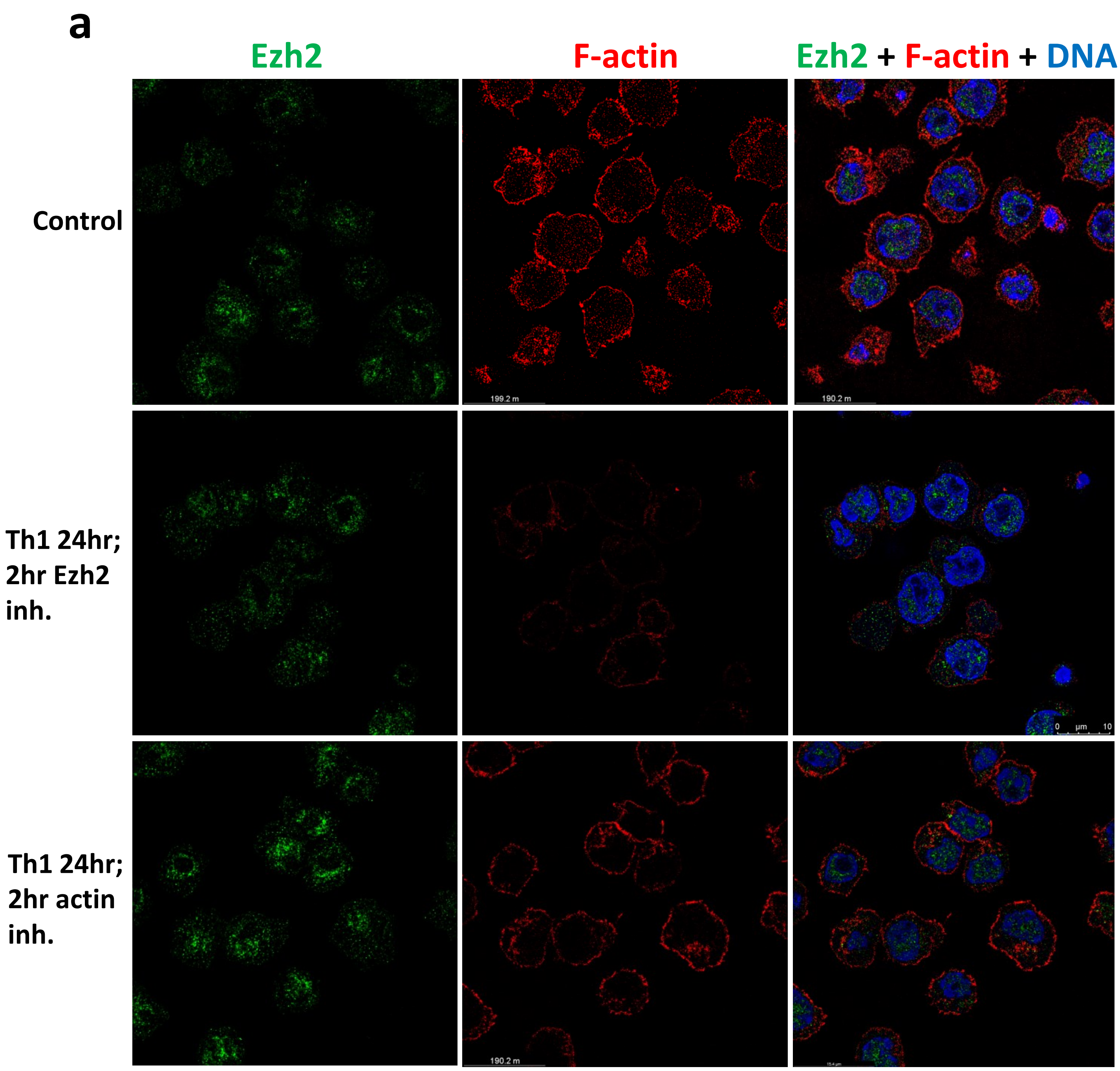
a
Ezh2
F-actin
Ezh2 + F-actin + DNA
Control
Th1 24hr; 2hr Ezh2 inh.
Th1 24hr; 2hr actin inh.
0 μm 10

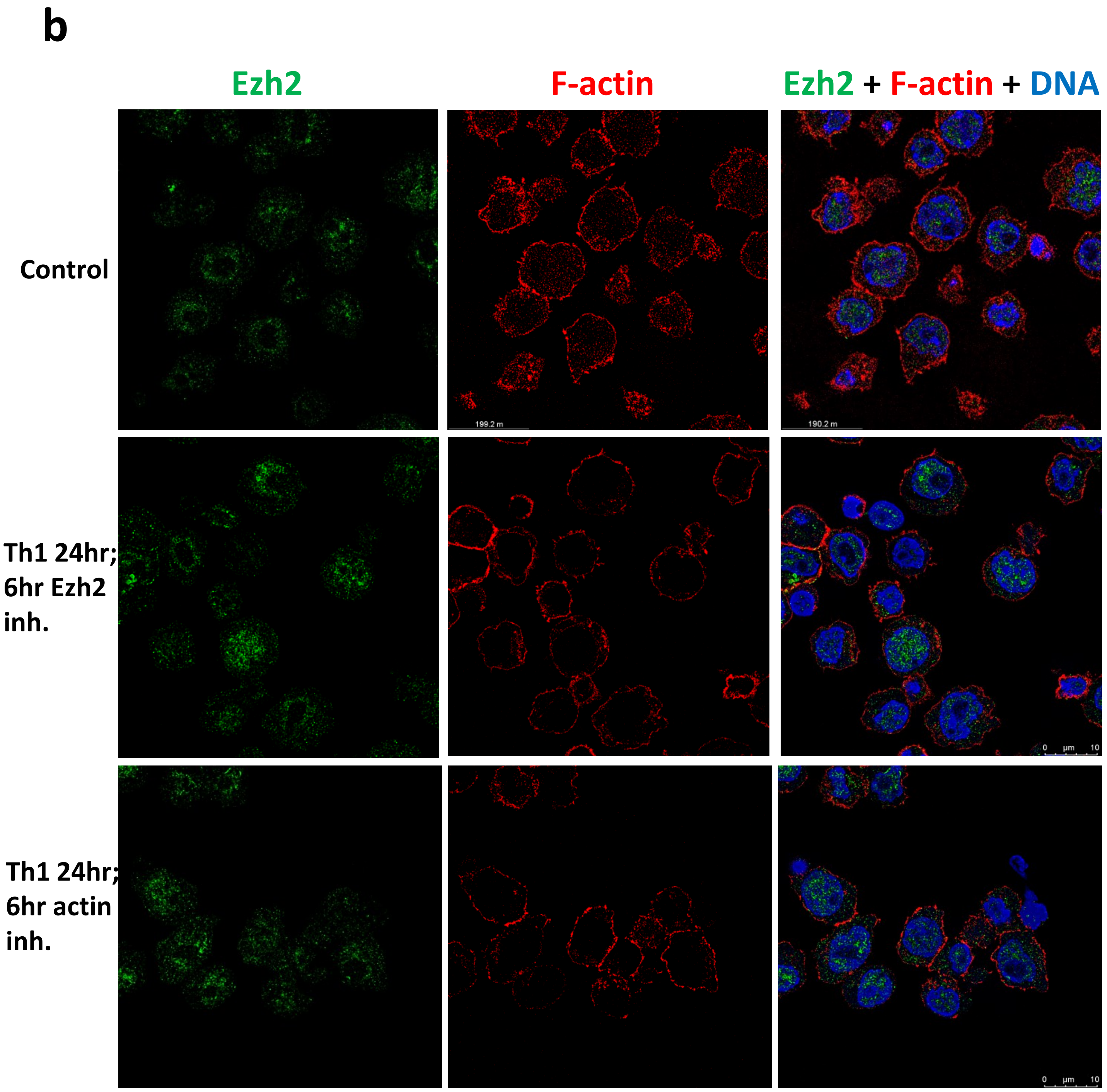
b
Ezh2
F-actin
Ezh2 + F-actin + DNA
Control
Th1 24hr; 6hr Ezh2 inh.
Th1 24hr; 6hr actin inh.
0 µm 10
0 µm 10

c

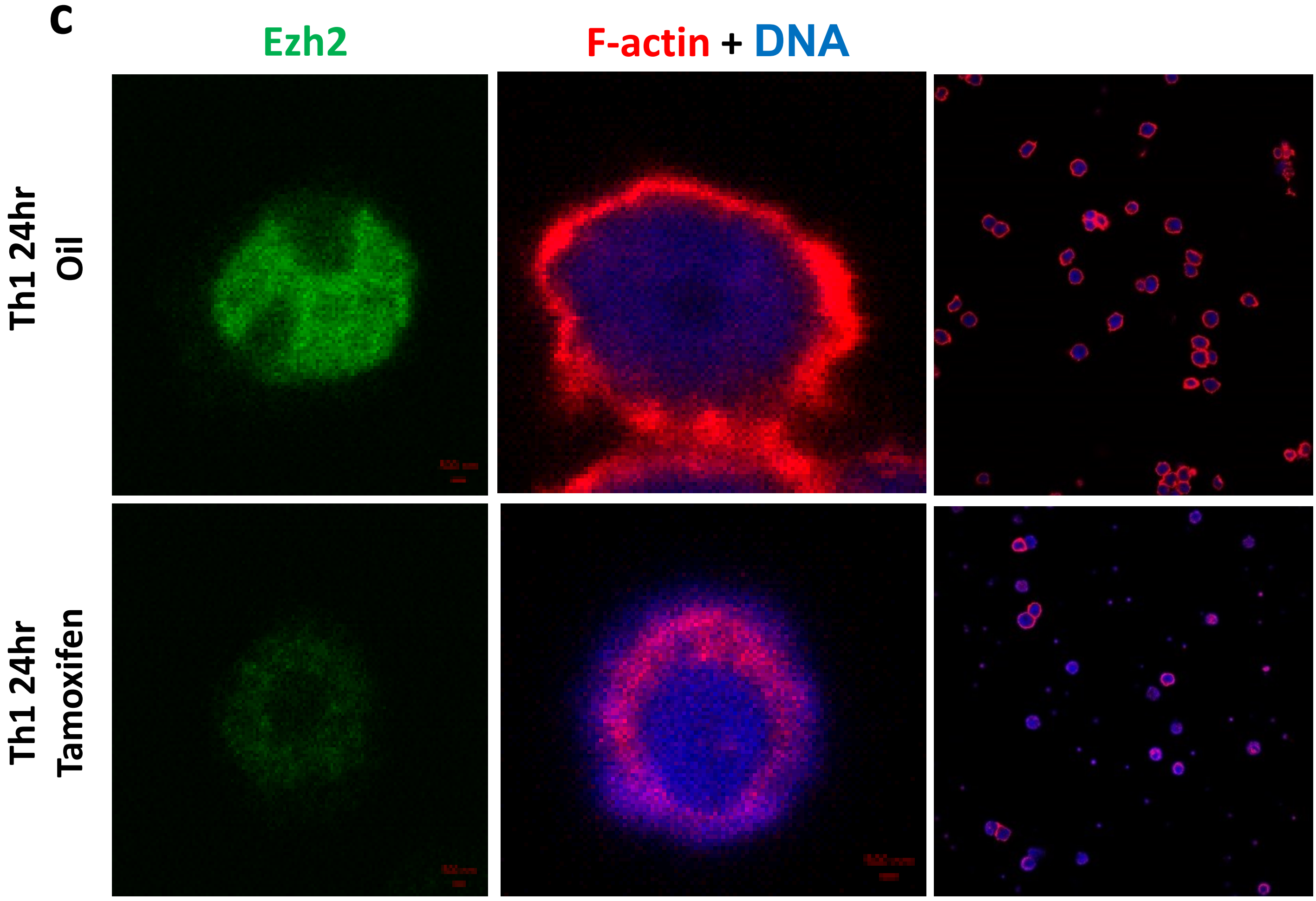

Figure S5 (refers to Fig. 5): **F-actin-dependent Ezh2.** Immunofluorescence image of 24hr-differentiating Th1 cells with either Ezh2 (1.75mM UNC1999) or actin (10.4mM cytochalasin B) inhibitors for the last 2hr **(a)** or 6hr **(b)** of stimulation. Staining was performed using phalloidin (red), simultaneously with anti-Ezh2 (green). Nuclei were stained with Hoechst (blue). The experiments were performed in three independent biological replicates with similar results. Images were acquired by SR Hyvolution microscope. **(c)** To assess the functional role of Ezh2 in differentiated cells, the expression of Ezh2 was ablated *in vitro* in conditional Ezh2- knock-out derived naïve Th cells expressing the cre-recombinase conjugated with Estrogen receptor from the Rosa26 promoter. The presence of (Z)-4-Hydroxytamoxifen (4-OHT) results in inducible translocation of the Estrogen receptor-cre from the cytosol to nucleus[72,73]. Preliminary experiments demonstrated that if 4-OHT was introduced with stimulation, Ezh2 protein was indeed ablated at the end of the first week of differentiation, however not at the first 48 hrs. To overcome this obstacle, tamoxifen or corn oil, as a control, were injected IV to the conditional Ezh2-knock-out mice for 5 days before the purification of the naïve cells. The 24hr-differentiating Th1 cells were stained with Phalloidin (red) simultaneously with anti-Ezh2 Ab (green). DNA was stained with DAPI (blue). Images were acquired by Zeiss LSM780 Inverted Confocal microscope. Although the expression of Ezh2 was not completely ablated, the nuclear actin filaments were absent. Instead, an unorganized ring of nuclear F-actin was performed, suggesting that Ezh2 regulates the spatiotemporal dynamics of F-actin. The experiments were performed in two independent biological replicates with similar results. Images were acquired by SR Hyvolution microscope.

Figure S6

a Hierarchical clustering heat map of differentially expressed genes in differentiating Th1 cell in the presence or absence of Ezh2 inhibitor UNC1999

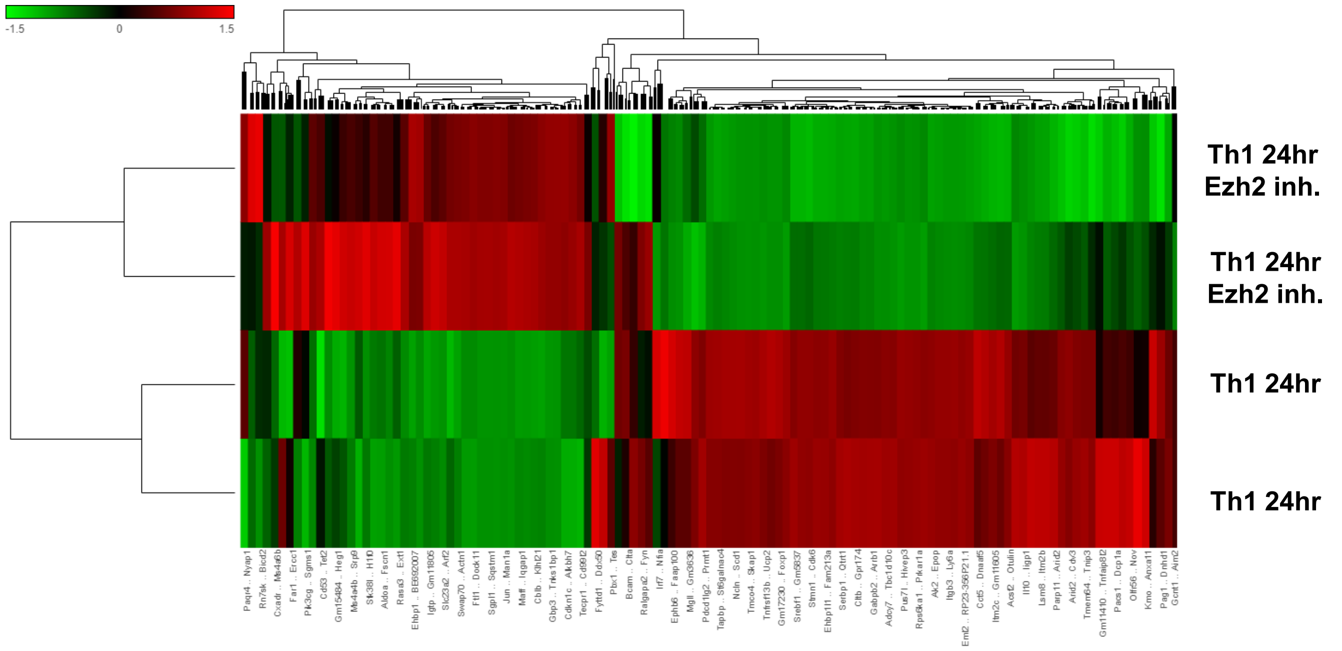

Hierarchical clustering heat map of differentially expressed genes in differentiating Th1 cell in the presence or absence of actin inhibitor cytochalasin B

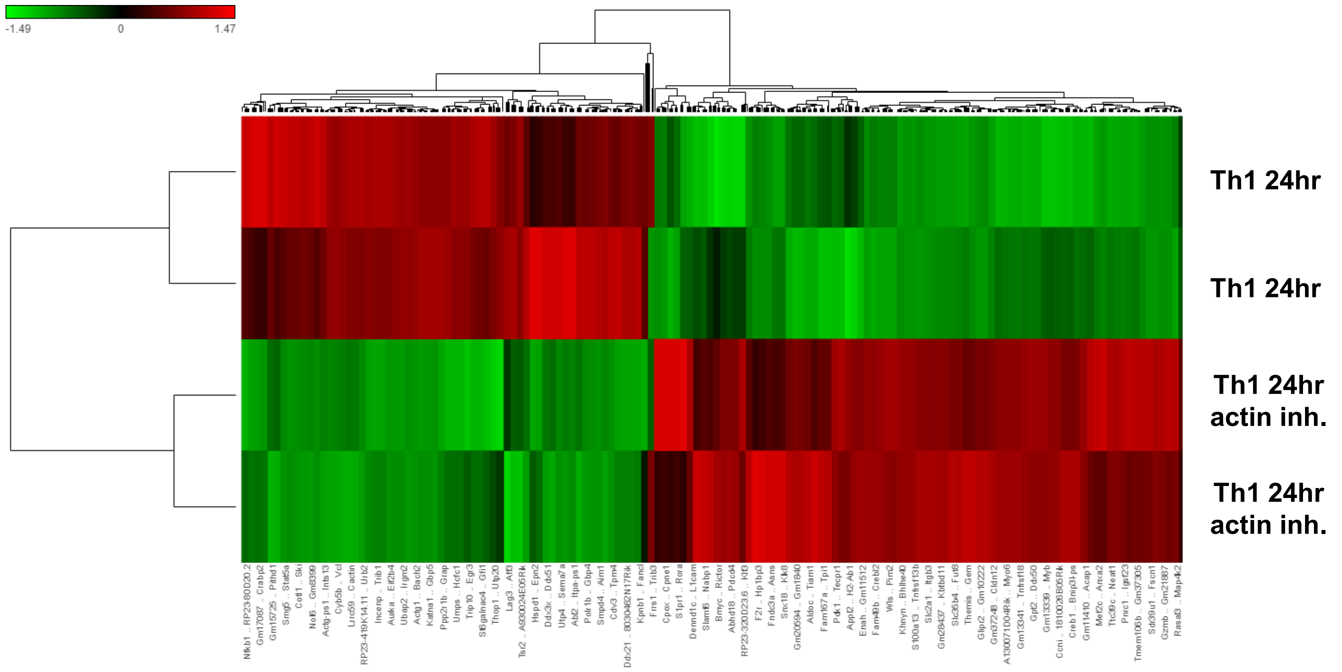

Hierarchical clustering heat map of differentially expressed genes in differentiating Th2 cell in the presence or absence of Ezh2 inhibitor UNC1999

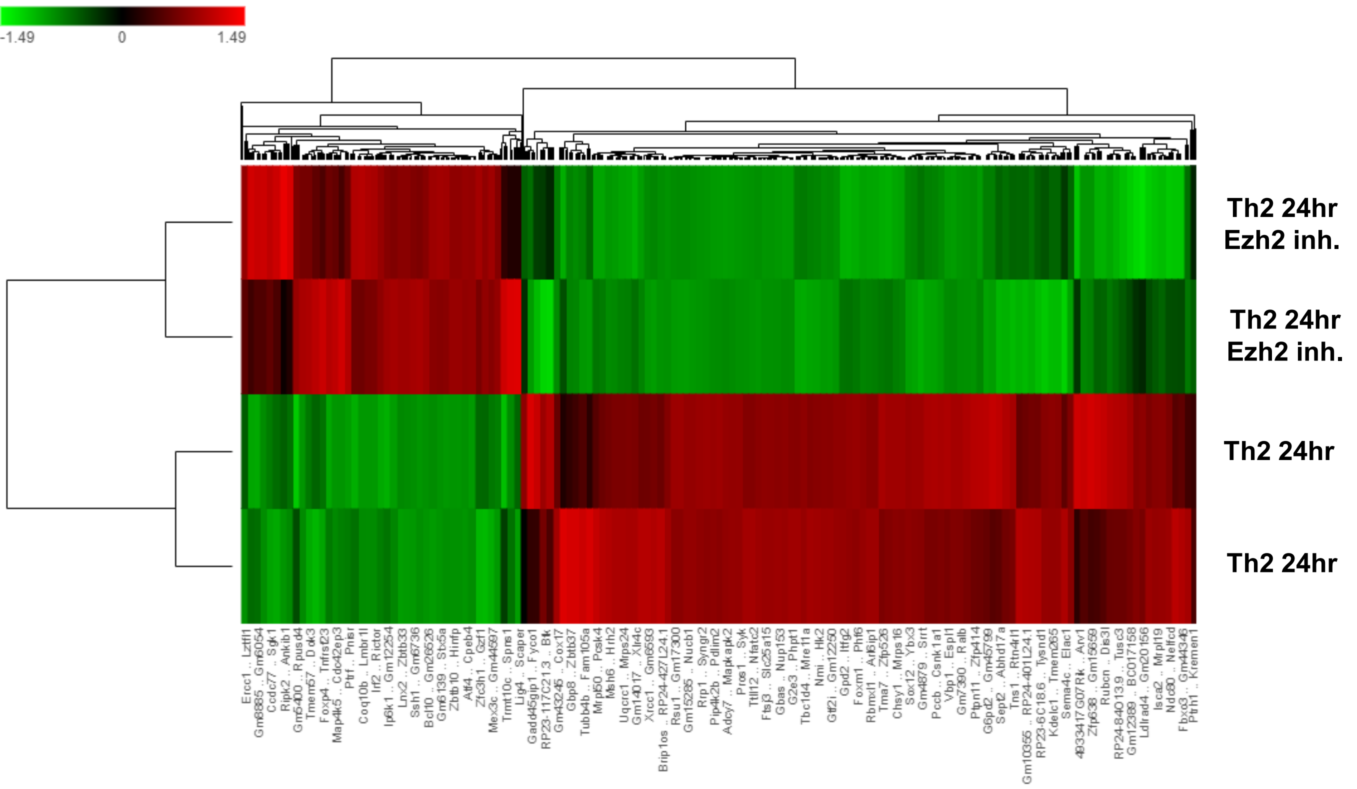

Hierarchical clustering heat map of differentially expressed genes in differentiating Th2 cell in the presence or absence of actin inhibitor cytochalasin B

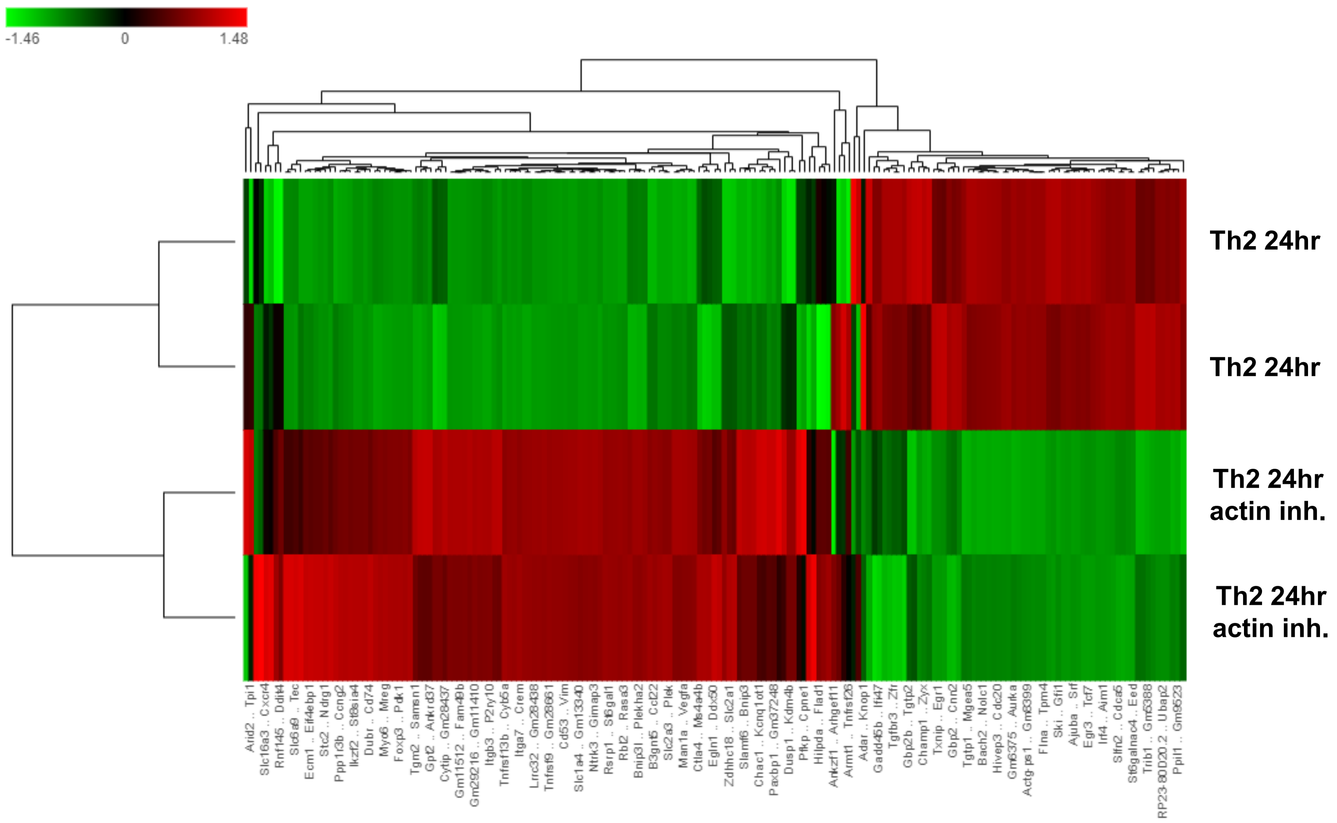

b

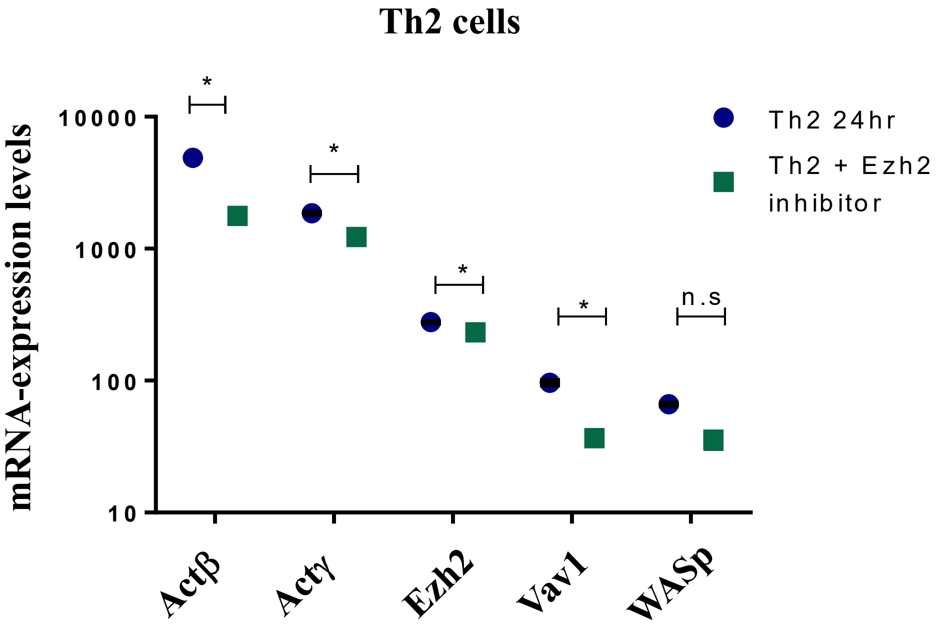
Th2 cells
mRNA-expression levels
10000
1000
100
10
*
*
*
*
n.s
Th2 24hr
Th2 + Ezh2 inhibitor
Actβ
Actγ
Ezh2
Vav1
WASp

c

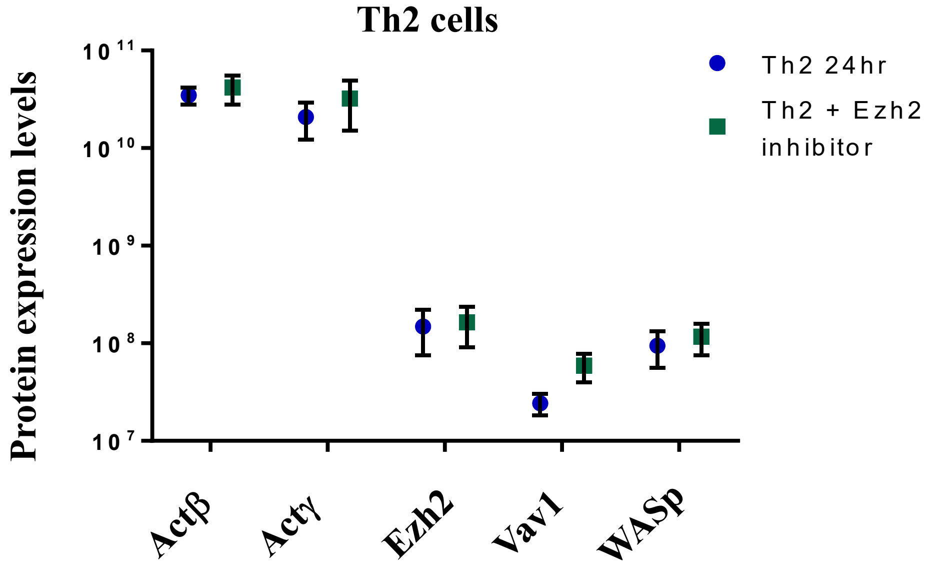
Th2 cells
Protein expression levels
10^{11}
10^{10}
10^{9}
10^{8}
10^{7}
Th2 24hr
Th2 + Ezh2 inhibitor
Actβ
Actγ
Ezh2
Vav1
WASp

d

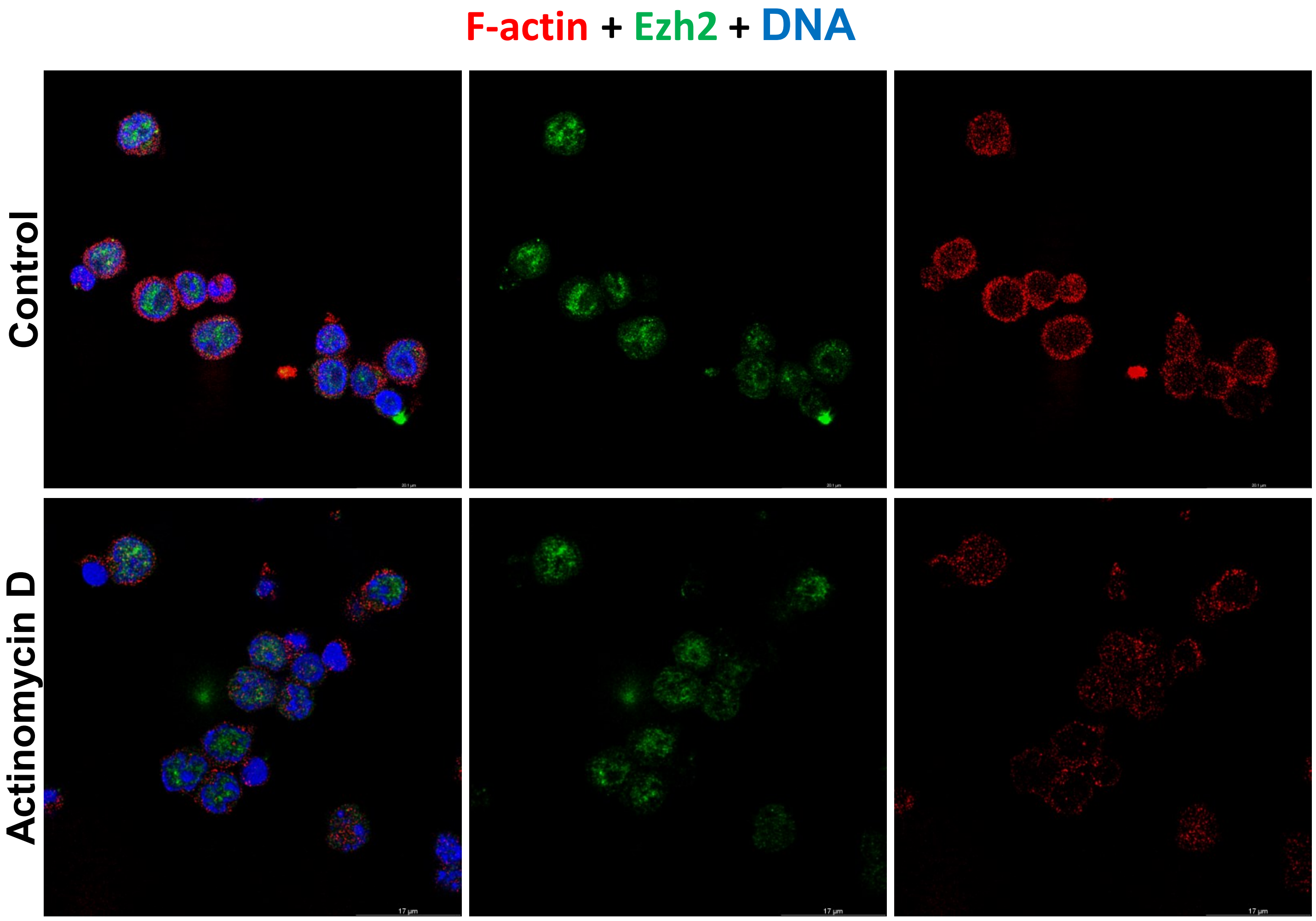
F-actin + Ezh2 + DNA
Control
Actinomycin D

Figure S6 (refers to Fig. 6): **Ezh2-dependent methyltransferase activity regulates both the transcription of actin and post-transcriptionally the assembly of F-actin**. **(a)** RNA-seq of 24hr-differentiating Th1 and Th2 cells in the presence or absence of either Ezh2 (1.75mM UNC1999) or F-actin (10.4mM cytochalasin B) inhibitors for the last 6hr of stimulation. Sequences were aligned using bowtie and were subjected to Heat Map analysis showing the differences between the presence or absence of the inhibitors. (**b)** Total mRNA counts in the 24hr-differentiated Th2 cells with or without the presence of the Ezh2 inhibitor UNC1999 for the last 6hr of stimulation, as determined by RNA-seq analysis. Two-tailed t-test was performed, P-value<0.01 *, n.s – not significant, P-value > 0.05. **(c)** The expression levels of the indicated proteins in the 24hr-differentiated Th2 cells with or without the presence of the Ezh2 inhibitor UNC1999 for the last 6hr of stimulation, as determined by nuclear proteomics. Two-tailed t-test was performed, P-value > 0.05. **(d)** Immunofluorescence staining of 24hr-differentiating Th1 cells using Phalloidin (red) with or without actinomycin D (1μM) inhibitor for the last 6hr of stimulation. The experiments were performed in two independent biological replicates with similar results. Images were acquired by SR Hyvolution microscope.

Figure S7

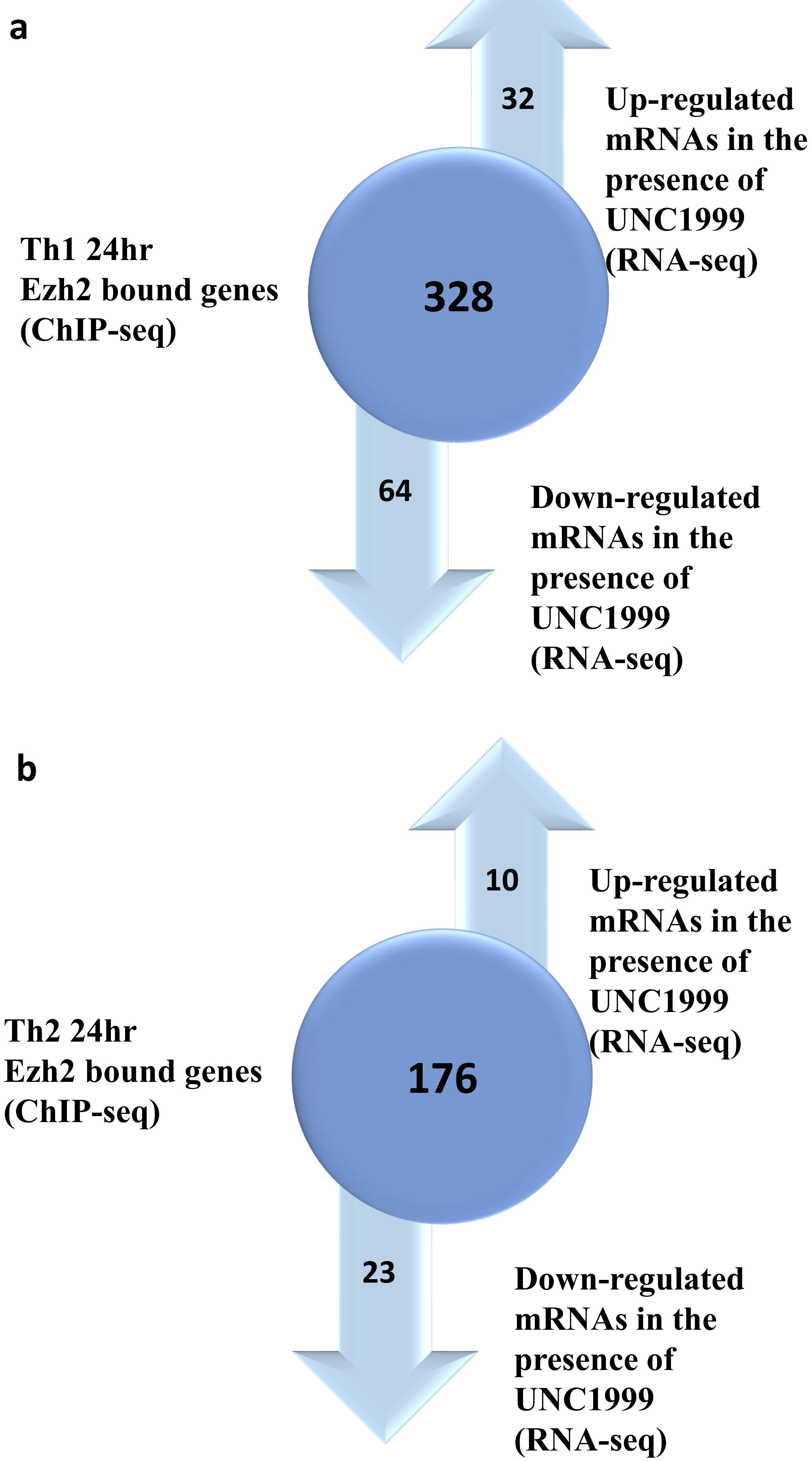
a
32
Up-regulated mRNAs in the presence of UNC1999 (RNA-seq)
Th1 24hr Ezh2 bound genes (ChIP-seq)
328
64
Down-regulated mRNAs in the presence of UNC1999 (RNA-seq)
b
10
Up-regulated mRNAs in the presence of UNC1999 (RNA-seq)
Th2 24hr Ezh2 bound genes (ChIP-seq)
176
23
Down-regulated mRNAs in the presence of UNC1999 (RNA-seq)

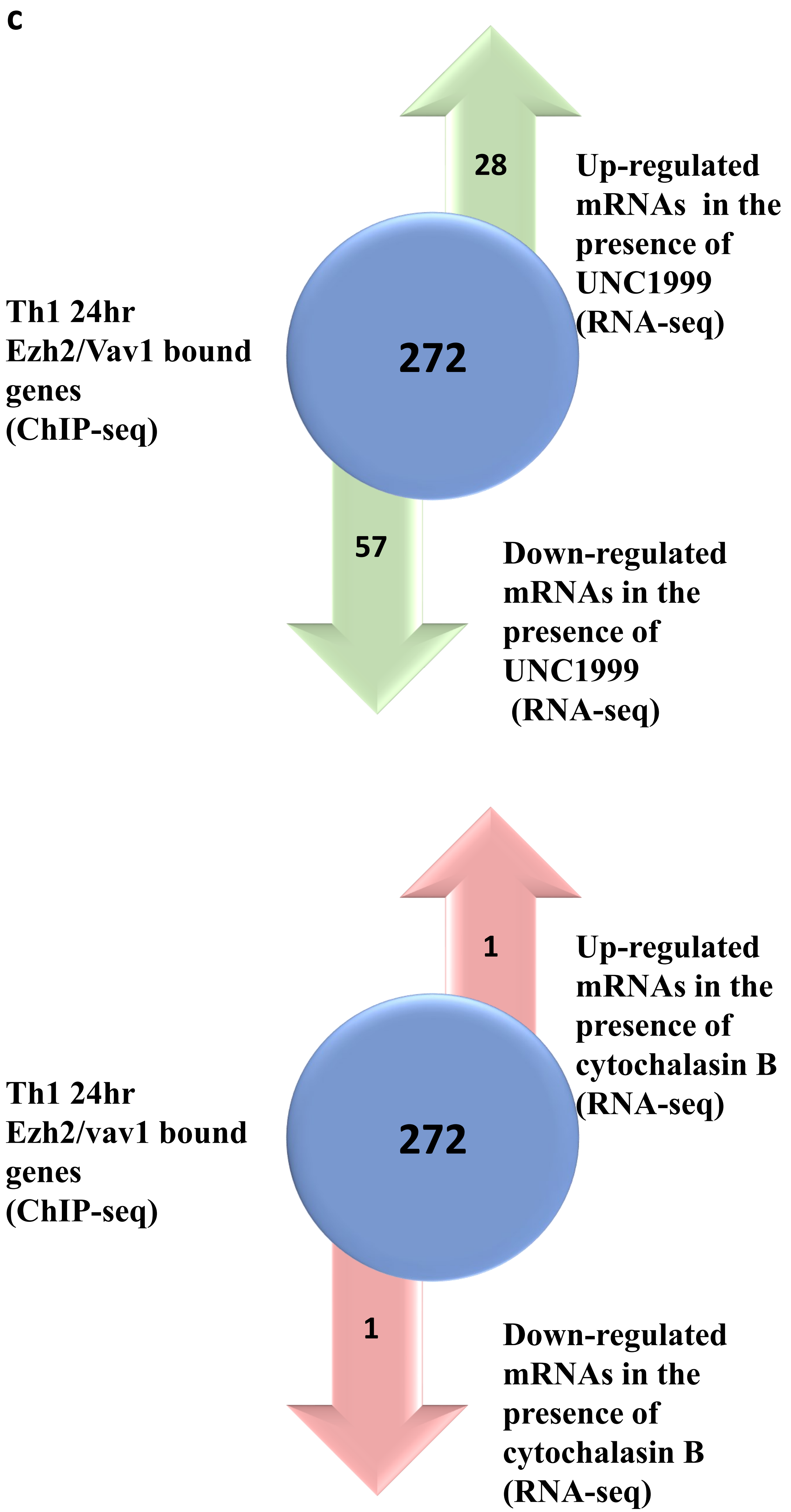
c
28
Up-regulated mRNAs in the presence of UNC1999 (RNA-seq)
Th1 24hr Ezh2/Vav1 bound genes (ChIP-seq)
272
57
Down-regulated mRNAs in the presence of UNC1999 (RNA-seq)
1
Up-regulated mRNAs in the presence of cytochalasin B (RNA-seq)
Th1 24hr Ezh2/vav1 bound genes (ChIP-seq)
272
1
Down-regulated mRNAs in the presence of cytochalasin B (RNA-seq)

d

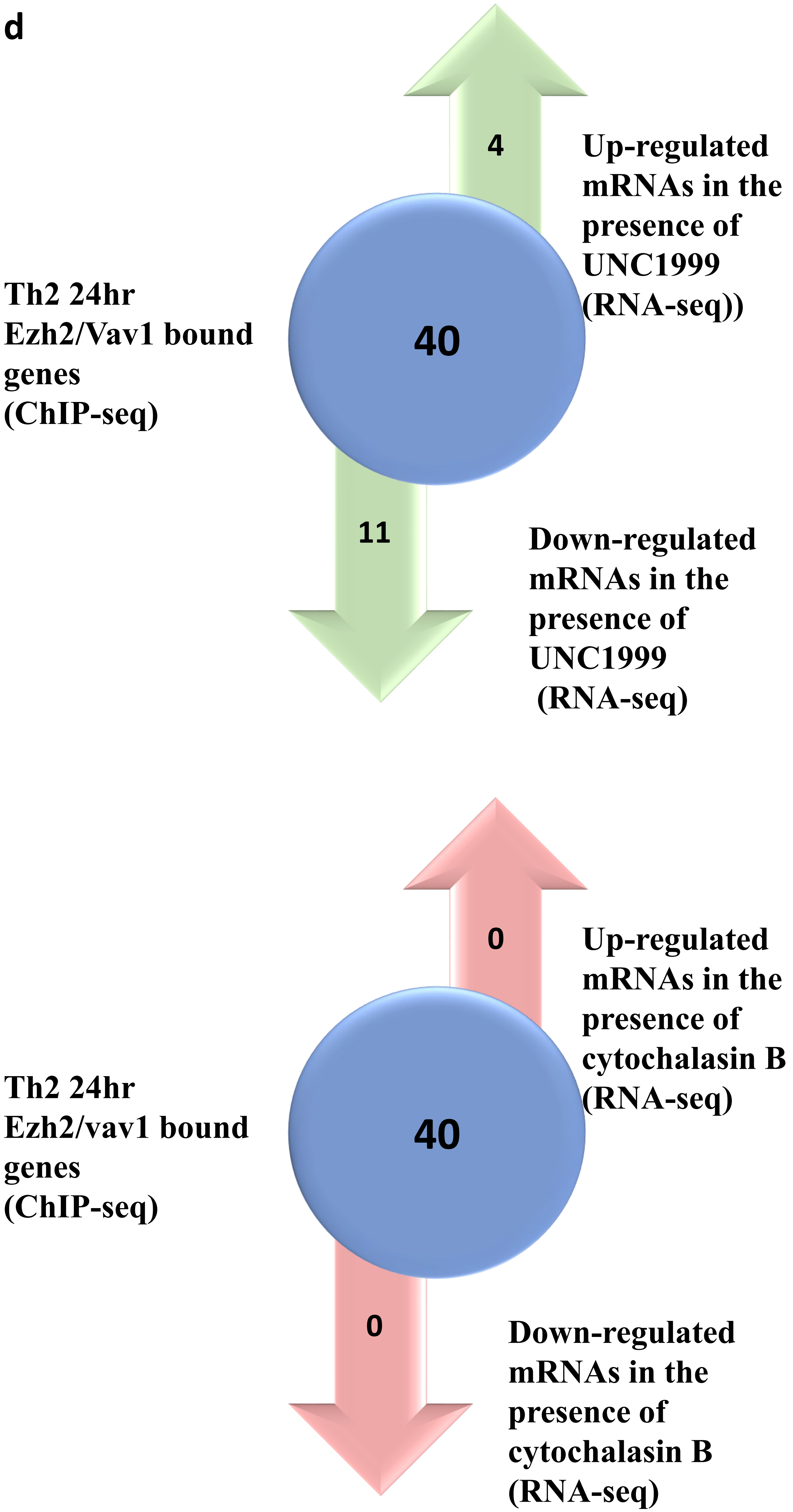
4
Up-regulated mRNAs in the presence of UNC1999 (RNA-seq))
Th2 24hr Ezh2/Vav1 bound genes (ChIP-seq)
40
11
Down-regulated mRNAs in the presence of UNC1999 (RNA-seq)
0
Up-regulated mRNAs in the presence of cytochalasin B (RNA-seq)
Th2 24hr Ezh2/vav1 bound genes (ChIP-seq)
40
0
Down-regulated mRNAs in the presence of cytochalasin B (RNA-seq)

e

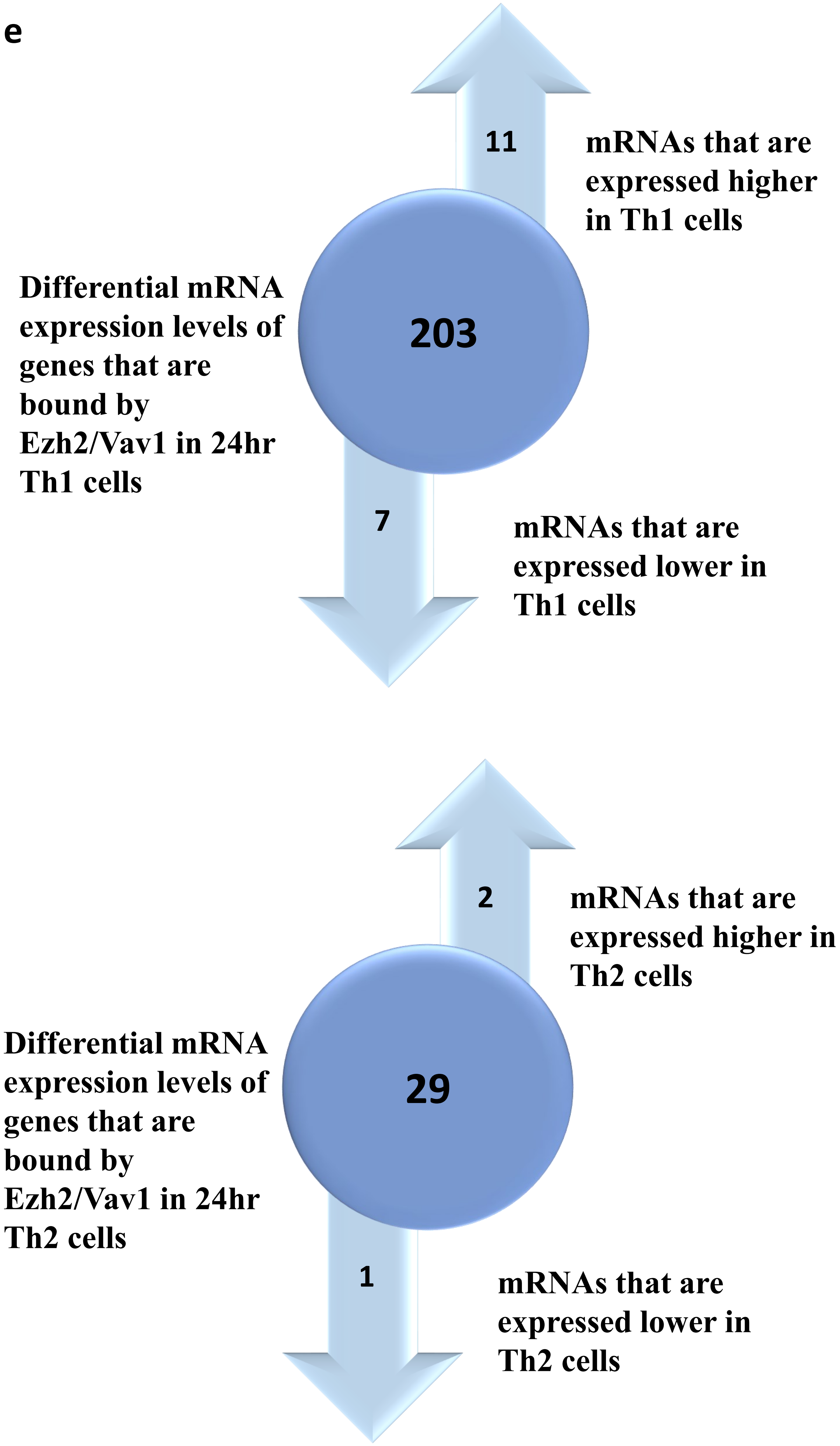
11
mRNAs that are expressed higher in Th1 cells
Differential mRNA expression levels of genes that are bound by Ezh2/Vav1 in 24hr Th1 cells
203
7
mRNAs that are expressed lower in Th1 cells
2
mRNAs that are expressed higher in Th2 cells
Differential mRNA expression levels of genes that are bound by Ezh2/Vav1 in 24hr Th2 cells
29
1
mRNAs that are expressed lower in Th2 cells

f

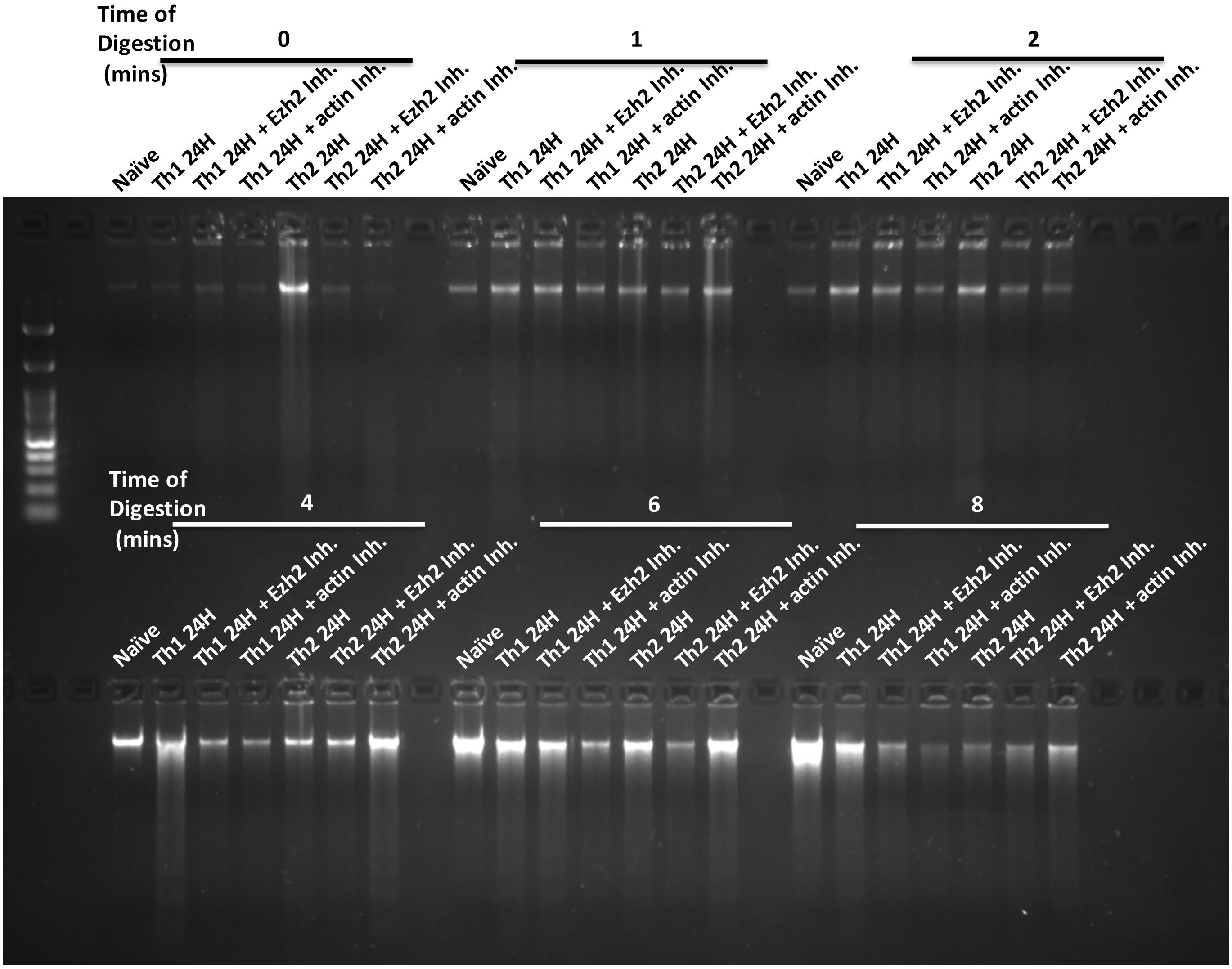
Time of Digestion (mins)
0
1
2
Naïve
Th1 24H
Th1 24H + Ezh2 Inh.
Th1 24H + actin Inh.
Th2 24H
Th2 24H + Ezh2 Inh.
Th2 24H + actin Inh.
Time of Digestion (mins)
4
6
8

Figure S7 (refers to Fig. 7): Ezh2 regulates chromatin spreading and nuclear expansion. (a-b) Integration of the Ezh2 ChIP-seq and RNA-seq data of the 24hr-differentiating Th1 **(a)** and Th2 **(b)** cells revealed that the expression levels of the Ezh2-bound genes were mostly unchanged in more than 1.5-fold in the presence of Ezh2 (UNC1999) inhibitor (Table S4a, S4b).**(c-d)** The mRNA levels of most of the Ezh2/Vav1 bound genes in either Th1 (c, and Table S5a, S5b) or Th2 (d and Table S5c, S5d) cells, were not dramatically changed (by more than 1.5-fold) in the presence of either Ezh2 or F-actin inhibitors. **(e)** The expression pattern of Ezh2/Vav1 bound genes was mostly non-differential between 24hr-differentiating Th1 and Th2 cells **(f).** Microccocal Nuclease Assay (MNase) of naïve and 24hr-differentiating Th1 and Th2 cells with or without the presence of either Ezh2 (UNC1999) or F-actin (cytochalasin B) inhibitor for the last 6hr of stimulation. The cells were permeabilized with Triton X-100 and treated with 1.71 units of MNase. The digestion was stopped at different time points (0,1,2,4,6,8 minutes). Untreated permeabilized cells were processed simultaneously (time point 0). The chromatin was isolated and run on 1.5% agarose gel. The molecular weight marker (100 bp ladder) was run on the left lane. The digestion of chromatin was only slightly delayed in the presence of actin and Ezh2 inhibitors.

Figure S8

F-actin + DNA

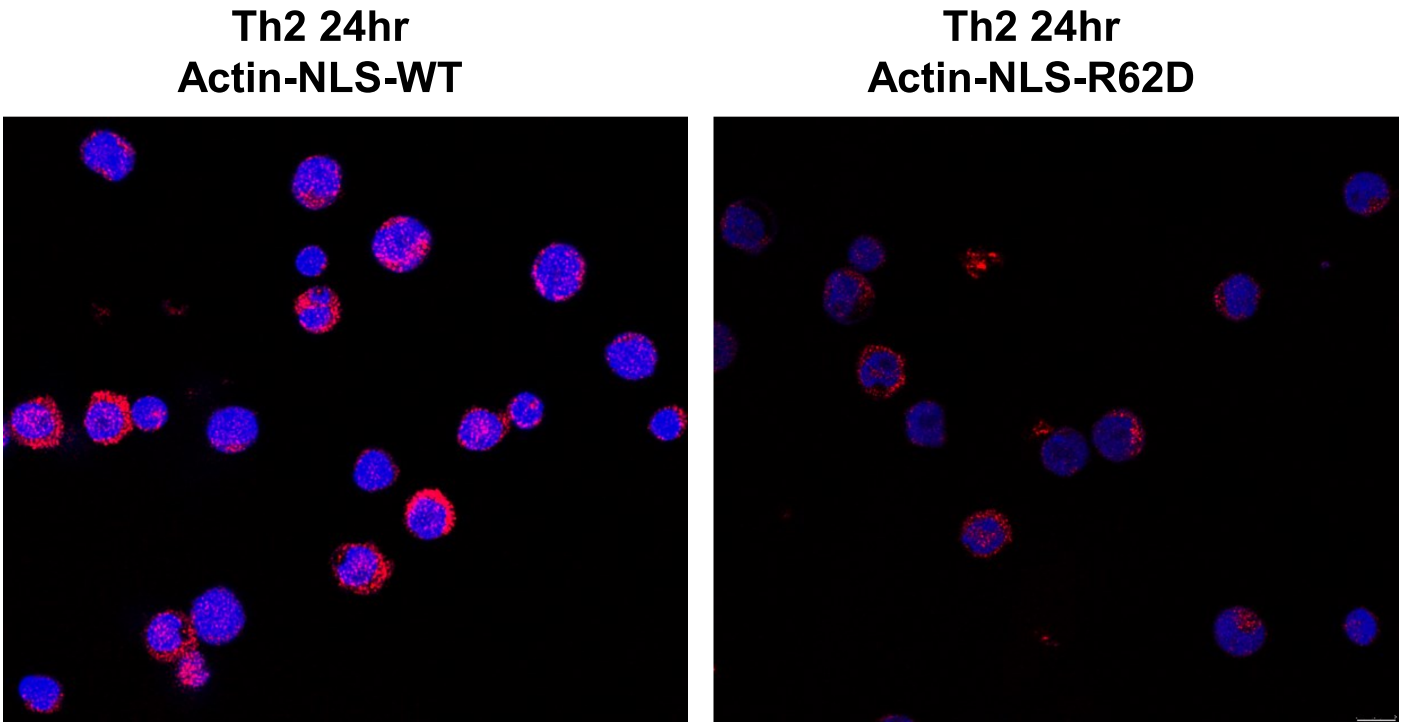

Figure S8 (refer to Fig. 8): **Ezh2 regulates chromatin spreading and nuclear expansion** 18hr differentiating Th2 cells were electroporated with the mRNA of either Actin-NLS-WT (25μg) or Actin-NLS-R62D (25μg) for the last 6hr of stimulation. The cells were stained with Phalloidin (red), and DNA was stained with Hoechst (blue). The experiments were performed in three independent biological replicates with similar results. Images were acquired by SR Hyvolution microscope.
